# Validation of participant eligibility for pre-exposure prophylaxis: Baseline data from the *PRELUDE* demonstration project

**DOI:** 10.1371/journal.pone.0185398

**Published:** 2017-09-26

**Authors:** Stefanie J. Vaccher, Andrew E. Grulich, Bridget G. Haire, Damian P. Conway, Isobel M. Poynten, Catriona Ooi, Rosalind Foster, David J. Templeton, Iryna B. Zablotska, on behalf of the PRELUDE Study Team

**Affiliations:** 1 Kirby Institute, UNSW Sydney, Kensington, New South Wales, Australia; 2 Sydney Sexual Health Centre, Sydney, New South Wales, Australia; 3 Western Sydney Sexual Health Centre, Parramatta, New South Wales, Australia; 4 Clinic 16, Northern Sydney Sexual Health, St Leonards, New South Wales, Australia; 5 RPA Sexual Health, Sydney Local Health District and Central Clinical School, The University of Sydney, Sydney, New South Wales, Australia; University of Maryland School of Medicine, UNITED STATES

## Abstract

**Background:**

In Australia, pre-exposure prophylaxis (PrEP) is targeted to individuals at high risk for HIV infection. We describe the HIV risk profile and characteristics of *PRELUDE* participants, and evaluate the population validity of the sample in representing high-risk gay and bisexual men (GBM) eligible for PrEP.

**Methods:**

*PRELUDE* is an on-going, open-label, single-arm observational study. Participants were identified in clinics and screened for eligibility using a paper-based risk assessment tool which followed the New South Wales (NSW) PrEP guidelines. Selection was validated using an independent online behavioural survey, completed by study participants upon enrolment. Demographic information was analysed using descriptive statistics, and kappa tests were used to determine agreement between reporting of high-risk practices in the risk assessment and behavioural survey.

**Results:**

During 2014–15, 471 individuals were targeted for enrolment; 341 were assessed for PrEP eligibility and 313 were enrolled. Of these, 303 (97%) identified as GBM. Overall, 85% of GBM met at least one high-risk criterion; 68% reported receptive intercourse with an HIV-positive or unknown status casual male partner, and 37% reported methamphetamine use in the three months preceding enrolment. The remaining 15% were enrolled based on medium-risk behaviours, or at the clinicians’ discretion. We found an 82% total agreement between self-reported high-risk behaviour and clinicians’ categorisation of GBM as being at high risk for HIV based on PrEP eligibility criteria.

**Conclusions:**

Behavioural eligibility criteria used by clinicians successfully identified individuals at high risk for HIV infection. This targeted approach ensures that the greatest public health and HIV prevention benefits can be derived in a setting without universal access to PrEP.

## Introduction

The efficacy of tenofovir (TDF)-based antiretroviral regimens for HIV pre-exposure prophylaxis (PrEP) has been unequivocally demonstrated in randomised controlled trials and open-label extension/demonstration projects [1]. Furthermore, PrEP was shown to be safe and effective in populations including men who have sex with men [2–4], men and women in heterosexual HIV-serodiscordant relationships [5], and people who inject drugs [6]. Two European studies, IPERGAY [3] and PROUD [4], independently reported that in high-risk homosexually-active men, PrEP reduced the risk of HIV acquisition by 86%.

In 2012, a fixed-dose combination of TDF and emtricitabine (FTC) was approved for HIV prophylaxis in the United States (US) for people at substantial risk of infection [7]. PrEP is now recommended in national and regional HIV guidelines in the US [8], South Africa [9], Australia [10], and Europe [11], as well as by the World Health Organisation (WHO) [12].

Despite having very high efficacy, particularly among homosexually-active men [13], PrEP delivery to key populations has varied considerably across jurisdictions [14–17]. In many settings, the widespread implementation of PrEP has been challenging due to lack of regulatory approvals and high medication costs [18–20]. Whilst PrEP availability remains restricted in some contexts, established access programs should be benefit-focused.

In Australia, the HIV epidemic is concentrated among gay and bisexual men (GBM), with a prevalence in this group of 14–18% over the past ten years (18% in 2015) [21]. TDF/FTC was not licensed for PrEP in Australia until May 2016 [22], and an application to Australia’s Pharmaceutical Benefits Committee for public subsidy of PrEP failed in August 2016 [23]. Given the limited access to PrEP, the New South Wales (NSW) [24] and then Australian guidelines [10] took a pragmatic approach and recommended targeting PrEP to individuals at high risk of HIV. Eligibility was based on local epidemiological data [25–28], and sought to maximise public health benefits.

The *PRELUDE* demonstration project was the first PrEP access program in NSW; Australia’s most populous state. The study was designed to assess the feasibility of PrEP delivery in NSW health services, and acceptability of PrEP among high-risk individuals. We present the baseline characteristics of our cohort of targeted PrEP users to evaluate the population validity of the sample in representing high-risk GBM eligible for PrEP based on current behavioural eligibility criteria in the state of NSW, Australia.

## Materials and methods

### Study design and population

**Fig 1:**
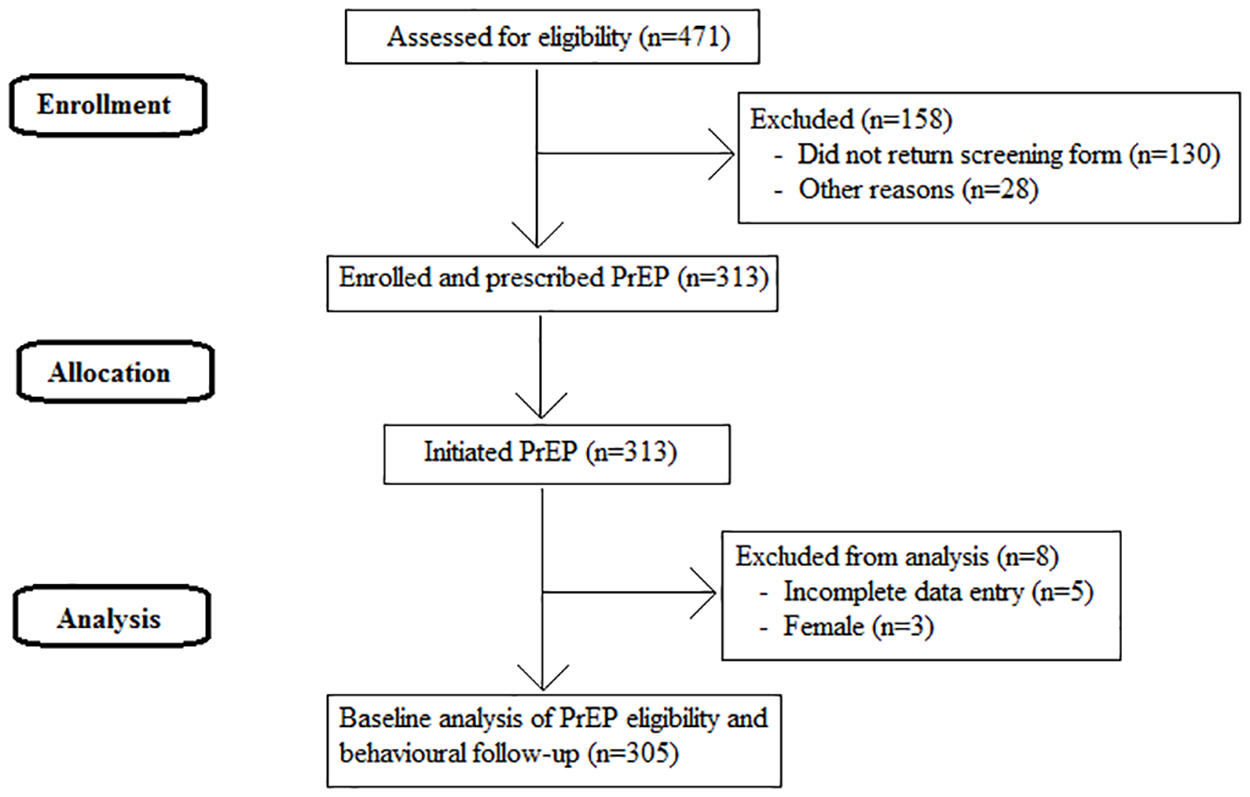
CONSORT flow diagram.

The study design and methods have been reported previously [29]. Participants’ movement through the study is tracked in Fig 1. The full study protocol (S1 File) and Trend Checklist (S2 File) are included in Supplementary Files 1 and 2.

**Fig 2:**
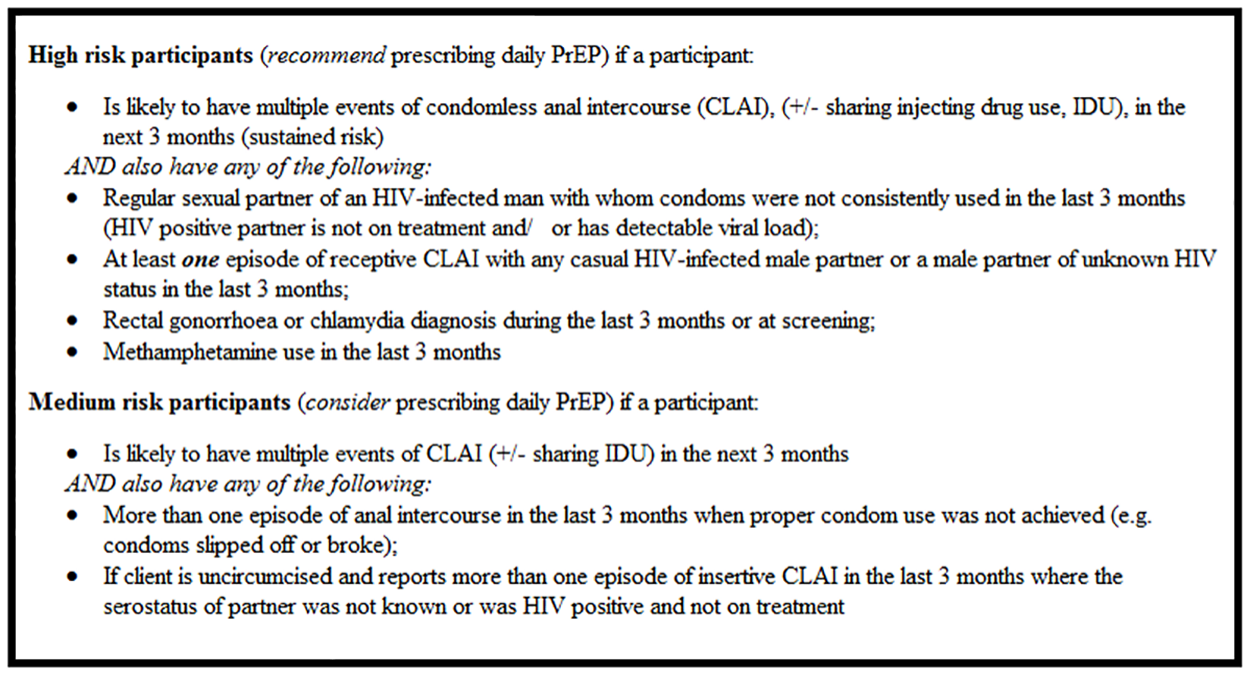
New South Wales HIV pre-exposure prophylaxis behavioural eligibility criteria for gay and bisexual men.

Briefly, *PRELUDE* is an open-label, single-arm demonstration project evaluating targeted PrEP delivery in NSW, Australia. The study design followed local PrEP guidelines [24] as to risk-based PrEP eligibility; that is, participants were enrolled if they disclosed specific behaviours indicating they were at high risk of acquiring HIV. Eligibility criteria for GBM are presented in Fig 2. Discretion could be applied by clinicians on a case-by-case basis for individuals who frequently attended services for HIV or other sexually transmissible infection (STI) testing, STI management, or post-exposure prophylaxis (PEP).

The target population was individuals at high risk of acquiring HIV, predominantly GBM. Due to the absence of regulatory approval for PrEP in Australia at the time of *PRELUDE* initiation, the study could not be publically advertised. No monetary incentives were used to encourage participation. The sample size (approximately 300 individuals) was informed by the then-available evidence, expressions of interest for PrEP, and the number of participants deemed feasible to enrol through clinics in NSW. We used convenience sampling and identified potential participants among the existing patients of the participating clinics, who had already been known to the staff to be at high risk of HIV, and among the new patients who self-referred after learning about *PRELUDE* from its website, friends, or by word of mouth. Potential participants were asked to complete the paper-based risk assessment form. Those who were found eligible for PrEP provided their medical history to clinicians, were screened for HIV, Hepatitis B, and other STIs, underwent liver and renal function tests, and had a discussion with their clinician about HIV risk-reduction strategies and adherence to PrEP. All participants were prescribed once daily oral Truvada (a combination pill of 300mg TDF and 200mg FTC, produced and donated to the study by Gilead Sciences Inc.). Participants were issued with a prescription for a 30-day medication supply, with instructions to take it at the same time every day, in accordance with their individual routines. The anticipated follow-up (up to 30 months) was aimed at evaluating primary outcomes, specifically, adherence to PrEP, behaviour, and HIV and STI infections among PrEP users. Inclusion and exclusion criteria and the full primary and secondary outcomes have been published previously [29]. Following their baseline study visit, participants were sent a personalised link to an online survey, containing questions about demographics, sexual practices, and attitudes to HIV and PrEP, to be completed in private, away from the clinic.

### Data collection

This manuscript uses data from the participant screening and enrolment visit, and the baseline behavioural survey. Eligibility screening was conducted using a paper-based risk assessment completed by participants prior to their study visit, followed by a discussion with their clinician. This consisted of basic demographics and 8 Yes/No eligibility questions about behavioural practices in the previous three months (S1 Table). Participants reporting at least one of the four high-risk practices were deemed eligible, with medium-risk participants able to be enrolled at clinicians’ discretion.

Behavioural surveys were conducted using an online platform, Survey Gizmo (Boulder, Colorado, USA). Participants were required to complete these surveys as soon as possible after their study visit, and were sent two email reminders one week apart, followed by a text or phone call if their survey remained incomplete. They were reassured that clinical staff would not have access to their survey results. Detailed data were collected on the number of sexual partners by their type and HIV status, and the number of episodes of anal intercourse with each of these partner types in the previous three months, stratified by condom use, ejaculation, and taking the insertive or receptive role during each event. Partners were classified into three categories: (1) main regular, such as a boyfriend or lover; (2) other regular, including friends (with benefits) or fuckbuddies; and (3) casual partners, with whom a person has not had sex with before. Demographic and attitudinal information was also collected in the survey. Survey questions used in this analysis are included in Supplementary File 3 (S3 File). Each participant was given a unique study identification number to enable linkage between the clinical and behavioural data collection systems.

### Statistical analysis

All statistical analyses were conducted using STATA 14.1 (StataCorp, College Station, TX, USA). Demographic information was analysed using descriptive statistics. Categorical or ordinal behavioural data were transformed into binary variables for ease of analysis, and differences between groups were assessed using χ^2^ tests. When a range of count data was given for the number of sexual events, the median was used in further analysis. Agreement between reporting methods was calculated using Cohen’s kappa statistic measure of interrater agreement [30]. Incomplete or missing data were excluded. All tests assumed a Type I Error of 5% (p<0.05).

### Ethical committee review

All participants provided written informed consent before undertaking any study procedures, in accordance with the Declaration of Helsinki. The study was approved by St Vincent’s Hospital Human Research Ethics Committee (HREC) in Sydney, NSW (protocol number HEPP 1403; identification number HREC/14/SVH/130) and registered under ClinicalTrials.gov (identifying number NCT02206555).

## Results

### Enrolment

Overall, 471 risk assessment forms were distributed, 341 were completed and 313 participants were enrolled between 20^th^ November 2014 and 31^st^ August 2015. Paper-based risk assessment data were available for 312 individuals, and completed online behavioural survey data were available for 309 individuals. Full baseline data were available for 308 participants (98%).

### Baseline demographics

Baseline demographic data are shown in Table 1. *PRELUDE* participants had a median age of 36 (range: 20–63) years and most identified as GBM (97%). The original study cohort included three women and two female-to-male transgender individuals. One-third of participants (n = 113, 36%) were born outside of Australia, most commonly in the United Kingdom (n = 21, 7%), New Zealand (n = 12, 4%), or the US (n = 7, 2%). *PRELUDE* participants were predominantly of Anglo-Australian descent (n = 209, 67%), followed by Southern European (n = 17, 5.3%), South American (n = 12, 4%) or Chinese (n = 11, 4%). Participants were highly educated, with two-thirds having obtained a university-level qualification or higher (n = 206, 66%), and the majority were employed full-time (n = 220, 70%) or part-time (n = 29, 9%). Approximately half of the men enrolled were circumcised (n = 151, 49%), with participants born in Australia significantly more likely to be circumcised than those born overseas (76% vs 53%; p<0.001). There were no demographic differences between participants meeting the high- or medium-risk criteria, or those attending public as compared to private clinics (p>0.05 for all).

**Table 1:** Demographic characteristics of *PRELUDE* participants at enrolment.

	Number (n = 313)	%^b^
**Age**		
<30	86	27%
30 to <40	114	36%
40 to <50	87	28%
≥50	26	8%
**Ethnicity**		
Anglo-Australian^a^	209	67%
Aboriginal or Torres Strait Islander	5	2%
Other	99	32%
**Born in Australia**		
Yes	200	64%
No	109	35%
Missing	4	1%
**Gender**		
Male	308	98%
Female	3	1%
Trans, Female-to-Male	2	1%
**Sexuality**		
Gay/homosexual	287	92%
Bisexual	16	5%
Other/missing	10	3%
**Employment**		
Full or part-time	249	80%
Student	16	5%
Unemployed	26	8%
Other/missing	22	7%
**Highest level of education**		
Did not complete high school	16	5%
High school or TAFE	88	28%
University or higher	206	66%
Missing	3	1%
**Circumcised**		
Yes	151	48%
No	155	50%
Other/missing	7	2%

^a^ Anglo-Australian made up of: Anglo-Celtic, British/Irish, Western European and Northern European, according to the Australian Bureau of Statistics Australian Standard Classification of Cultural and Ethnic Groups, 2011

^b^ Some values may not add to 100% due to rounding

TAFE, Technical and Further Education

### Behavioural eligibility criteria

**Fig 3:**
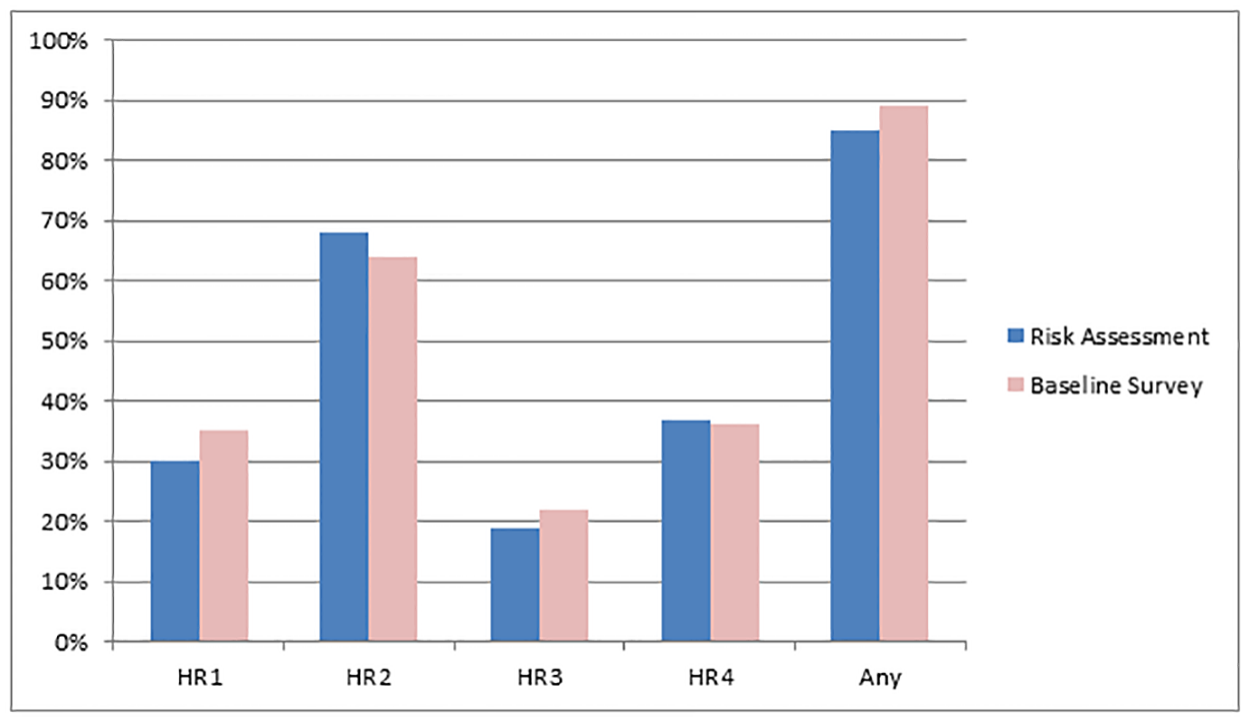
Comparison of high-risk behaviours reported in the paper-based risk assessment and online behavioural survey (n = 305). HR1: Had an HIV-positive regular partner with whom condoms were not consistently used; HR2: Had receptive intercourse with a casual male partner of HIV-positive or unknown status; HR3: Been diagnosed rectal chlamydia or gonorrhoea; HR4: Methamphetamine use.

Of the 313 enrolled participants, 263 participants (84%) reported at least one high-risk criterion on their paper-based risk assessment. All three women enrolled in the study met medium-risk criteria of seeking to conceive naturally with an HIV positive partner in the next three months, and given the small number of participants in this group, they were excluded from further analyses. Fig 3 presents the proportion of GBM with complete baseline data (n = 305) reporting high-risk practices in the paper-based risk assessment (n = 259, 85%) and online behavioural survey (n = 271, 89%). The paper-based risk assessment indicates that 46 GBM participants (15%) reported medium-risk criteria only (n = 43) or were enrolled based on the prescriber’s clinical judgement (n = 3).

Among 259 GBM who reported any high-risk criterion in the paper-based risk assessment, 95 (37%) met two or more high-risk criteria, and 13 (4%) met all four high-risk criteria. The most common high-risk criterion reported was having had receptive intercourse with a casual male partner of HIV-positive or unknown status (n = 208, 68%), followed by methamphetamine use (n = 114, 37%). Over one-quarter of participants (n = 86, 28%) reported both of these behaviours. Furthermore, 91 participants (30%) reported having condomless intercourse with a regular partner who was HIV-positive, and 58 participants (19%) reported having a rectal gonorrhoea or chlamydia diagnosis in the three months preceding baseline.

### Agreement between reporting methods

The results of the online behavioural survey, which was completed by participants in private, away from the clinic, within the first week after their baseline visit, were used to validate the responses given in the paper risk assessment forms completed together with a clinician prior to enrolment into *PRELUDE*. Overall, fair levels of agreement [31] were observed between the reporting of high-risk behaviours in the paper-based risk assessment and online behavioural survey (Table 2), ranging from 65% to 87%, with 82% total agreement (kappa = 0.23) in reporting any high risk practice. Thirty-three people (11%) who did not report any high-risk behaviour in their paper-based risk assessment reported at least one of the four high-risk criteria in their online behavioural survey. Furthermore, 21 people (7%) who reported at least one high-risk behaviour in their paper-based risk assessment did not report any of the four high-risk behaviours in their online behavioural survey. However, there was no significant difference (p = 0.69) in the mean number of high-risk criteria reported in the paper-based risk assessment or online behavioural survey.

**Table 2:** Agreement in reporting high-risk behavioural practices between paper-based risk assessment and online behavioural survey for gay and bisexual men (n = 305).

High-risk behavioural eligibility criteria ^a^	Not reported in either risk assessment or behavioural survey n. (%) ^b^	Only reported in risk assessment n. (%) ^b^	Only reported in behavioural survey n. (%) ^b^	Reported in both risk assessment and behavioural survey n. (%) ^b^	Overall agreement (%); kappa statistic
HIV-positive regular partner	170 (56%)	28 (9%)	44 (14%)	63 (21%)	67%; 0.46
Receptive intercourse with a casual partner	49 (16%)	60 (20%)	48 (16%)	147 (49%)	65%; 0.21
Rectal gonorrhoea or chlamydia diagnosis	220 (72%)	18 (6%)	27 (9%)	40 (13%)	85%; 0.55
Methamphetamine use	178 (57%)	21 (7%)	18 (6%)	93 (30%)	87%; 0.73
*Any high risk criterion*	*13 (4%)*	*21 (7%)*	*33 (11%)*	*238 (78%)*	*82%; 0*.*23*

^**a**^ All high risk criteria are assessed in the previous three months in both the paper-based risk assessment and online behavioural survey

^**b**^ Some values may not add to 100% due to rounding

### Sexual and behavioural characteristics

Table 3 presents sexual and behavioural practices reported in the online behavioural survey for the three month period preceding study enrolment, among GBM participants with complete data entry (n = 305). Almost two-fifths (n = 120, 38%) of participants reported having a main regular partner, and one-third (n = 40) of these partners were HIV-positive, although 78% (n = 31) reported that their partner had an undetectable viral load (UVL). Amongst GBM with an HIV-positive main regular partner, 95% (n = 38) reported having condomless anal intercourse (CLAI), and 73% (n = 29) reported having receptive condomless anal intercourse (rCLAI) with this partner. In the 3-month period prior to enrolment, participants reported an average of 31 anal sex events (range 0–263) with main regular partners of any HIV status, with no difference in the number of insertive and receptive events (p = 0.87), although the mean number of receptive anal sex events with ejaculation was significantly higher than mean number of withdrawal events (6.26 vs 3.98, p = 0.008).

**Table 3:** Sexual partners and practices of gay and bisexual male participants (n = 305) in the three months preceding baseline.

	Number	%
HIV+ main regular partner	40	13%
CLAI with HIV+ main regular partner	38	95%
Other regular partner	229	75%
CLAI with other regular partner	202	88%
Casual partner	271	89%
CLAI with casual partner	226	83%
Any self-reported STI	111	36%
Chlamydia	76	25%
Gonorrhoea	65	21%
Syphilis	15	5%
Used crystal meth	111	36%
Injection drug use	65	21%
Heard of post-exposure prophylaxis (PEP)	278	91%
Post-exposure prophylaxis (PEP) use	97	32%
Heard of pre-exposure prophylaxis (PrEP)	287	94%
Pre-exposure prophylaxis (PrEP) use	30	10%
Used party drugs for sex	161	53%
Had group sex (three or more men)	209	69%
Had group sex under the influence of party drugs	128	42%
HIV test in the last 3 months	269	88%

CLAI, condomless anal intercourse; PEP, post-exposure prophylaxis; PrEP, pre-exposure prophylaxis, STI, sexually transmitted infection

Three-quarters (n = 229, 75%) of participants had other regular partners, and 88% (n = 202) reported any CLAI with these partners. Overall, participants reported having had on average 3 (range 1–30) HIV-positive other regular partners, with a mean of 8 anal sex events (range 0–42), and 55% reported rCLAI with these partners. There were an average of 18 anal sex events (0–143) reported with a mean of 9 (1–150) HIV-negative other regular partners, and 72% of participants reported engaging in rCLAI with these partners. Similarly, an average of 19 anal sex events (0–143) were reported with a mean of 10 (0–150) other regular partners of unknown HIV status, and 66% of GBM reported rCLAI these partners.

Almost 90% (n = 271, 89%) of participants reported casual sexual partners, and overall, 83% (n = 226) reported engaging in CLAI with these partners. Overall, participants reported an average of 4 (range 0–35) HIV-positive casual partners, with a mean of 7 anal sex events (0–60), and 51% reported rCLAI with these partners. There were an average of 15 anal sex events (0–161) reported with a mean of 11 (1–250) HIV-negative casual partners, and 62% of participants reported engaging in rCLAI with these partners. Furthermore, an average of 17 anal sex events (0–306) were reported with a mean of 12 (0–120) casual partners of unknown HIV status, and 58% of GBM reported rCLAI with these partners.

In the three months preceding enrolment, approximately half of all participants (n = 161, 53%) reported using party drugs for sex, 36% (n = 111) reported methamphetamine use, and almost one-third of participants (n = 97, 32%) had used PEP.

### Comparison between high- and medium-risk participants

Participants enrolled in *PRELUDE* through meeting the high-risk behavioural eligibility criteria were significantly more likely than medium-risk participants to report a number of other practices that placed them at elevated risk of HIV acquisition. These included injection drug use (IDU, 24% vs 4%; p = 0.001), using party drugs for sex (58% vs 26%; p<0.001), group sex (71% vs 53%; p = 0.014), or group sex under the influence of party drugs (47% vs 17%; p<0.001). Participants who reported high-risk criteria were also significantly more likely than medium-risk participants to have used PEP in the three months prior to baseline (34% vs 19%; p = 0.004).

## Discussion

*PRELUDE* was established to provide access to PrEP and evaluate the performance of the NSW PrEP guidelines [24] in identifying individuals at high risk of HIV infection. Behavioural eligibility criteria contained in the NSW PrEP guidelines were used to screen potential participants, and their enrolment in *PRELUDE* was validated using an independent online behavioural survey conducted within a week of enrolment into the study. This study confirmed that guidelines can be used to target PrEP delivery to people who would benefit most from improved access. Furthermore, allowing clinicians to exercise their discretion when prescribing PrEP to people who did not disclose, but were suspected of engaging in, high risk practices was warranted.

Upon completion of the risk assessment, 85% of the enrolled GBM reported to clinicians that they had engaged in one or more high-HIV risk behaviours which comprise the PrEP eligibility criteria in the three months preceding baseline. This included receptive intercourse with a casual HIV-positive or unknown status male partner (68%), or methamphetamine use (37%). Furthermore, 30% of participants had a regular HIV-positive sexual partner, and 19% had been diagnosed with rectal gonorrhoea or chlamydia.

These high-HIV risk behaviours reported to clinicians via the risk assessment were validated through an independent online behavioural survey, with a high total agreement observed (82%). This assessment for consistency between the two different reporting measures was important to determine if there were any significant reporting biases when disclosing behaviours to clinicians that may be stigmatised (e.g. anal sex) or illegal (e.g. methamphetamine use). Inclusion of indirect indicators of sexual behaviour such as STI diagnoses was also necessary to ensure that any participants who did not feel comfortable in reporting other behaviours to clinicians could still be considered as eligible for PrEP based on these criteria.

Increased risk behaviours reported in the paper-based risk assessment may be indicative of the nature of PrEP provision in Australia, where potential study participants may exaggerate risk behaviours to appear eligible for PrEP through demonstration projects, given the high cost of the drug and the legal complexities involved in accessing an off-label prescription of self-importing a medication from overseas. Conversely, higher levels of risk behaviours reported in the online behavioural survey suggests that study participants may not be comfortable disclosing risk practices to clinicians. Given that none of the four risk behaviours examined were reported substantially more frequently in either the risk assessment or online behavioural survey, combined with the high percentage agreement between the two measures, this indicates that the findings, and thus the selection of the study population, were not substantially impacted by under- or over-reporting of high-risk behaviours.

Furthermore, a high percentage agreement between reporting measures can be of greater importance than a large kappa value in determining the level of consistency between two different measures. Kappa agreements are affected by sample size, expected agreement, and the unequal distribution of data across the four possible response categories [31]. On the other hand, percentage agreements are directly comparable between studies, and ‘guessing’ answers [31] is unlikely in this context, given that participants were not making a subjective judgement, but rather, objectively reporting past behaviours. This suggests that the reported rates of behaviours in *PRELUDE* are fair indicators of HIV risk, and thus, supports the targeted selection of the study cohort [30].

Despite some flexibility in the enrolment of *PRELUDE* participants, when compared with other PrEP studies and samples of Australian GBM it is evident that this cohort is at extremely high risk of HIV. Although detailed data about participants’ behaviour are lacking, previous studies have found substantially lower rates of IDU [32, 33] and methamphetamine use [33, 34], and similar proportions of STIs [33], rCLAI [33], and previous PEP use [34]. More detailed information on participant characteristics beyond basic demographics is required to gain a deeper understanding of the varied behavioural practices individuals engage in which make them suitable candidates for PrEP.

In comparison to the community-based sample of HIV-negative men who completed the 2015 Sydney Gay Community Periodic Survey [35], *PRELUDE* participants reported a range of behaviours that placed them at considerable risk of HIV. This was not limited to the practices contained in the paper-based risk assessment, but included behaviours such as IDU, group sex, or previously using PEP or PrEP. This confirms that the behavioural eligibility criteria successfully identified a cohort at high risk of HIV from amongst the broader Sydney GBM population.

The *PRELUDE* cohort is comprised of educated, gay community-connected, and highly motivated individuals who are actively engaged in their health care. As this study was conducted predominantly in urban Sydney, knowledge of new HIV prevention strategies is likely to be higher than amongst the general GBM population, due to the vast differences in geographic location, gay networks and community attachment, and knowledge across different regions of Australia. In order to ensure access and broader dissemination of PrEP, carefully considered education and targeted approaches need to be utilised.

Several aspects of the study design strengthened the findings. Although *PRELUDE* targeted individuals at high risk of HIV, some participants who did not report meeting the high-risk behavioural eligibility criteria were enrolled on an as-need basis. The inclusion of participants that meet less stringent eligibility criteria illustrates the difference between an implementation study and a clinical trial. A clinical trial may enforce high-risk eligibility criteria to maximise study power, whereas an implementation study assesses behaviours in a context closer to ‘real-world’ circumstances. However, there were some interesting differences in risk behaviours identified between participants who met the high-risk behavioural eligibility criteria, compared with those who did not. As well as reporting at least one of the high-risk criteria, this group also reported higher rates of group sex, IDU, party drug use, and PEP use in the three months preceding baseline. However, all study participants reported a range of behaviours that placed them at substantial risk of HIV, and enabling such individuals to access PrEP mimics prescribing practices that are likely to occur once PrEP becomes widely available in Australia.

There are several limitations to this study. Firstly, there was a moderate sample size and geographical recruitment area, and many of the study participants were already accessing services at the participating clinics when they enrolled in the study. Thus, this sample may not be broadly representative of GBM in Australia, or internationally, particularly those not engaged in the healthcare system. However, this study targeted a group of individuals at high-risk of HIV, in order to maximise the public health benefits of PrEP whilst availability remains restricted in Australia. Secondly, self-reported data, which informs the majority of this paper, is subject to recall and social desirability bias. Nonetheless, the online behavioural surveys, which were completed in private so clinicians were not aware of participants’ responses, had a fair level of agreement with the practices reported to clinicians in the risk assessment, validating its use in identifying individuals at high risk of HIV. Thirdly, the research team has been in close collaboration with study partners and the community sector for some time, and most participants were highly engaged with the study, suggesting a motivated and altruistic cohort, which may further impact the generalisability of the results.

There were also some inconsistencies in data collection; however these should not have impacted on the overall findings. Participants were only asked about the viral load of HIV-positive main regular partners, not other regular or casual partners, and the paper-based risk assessment did not enquire about viral load measurements when reporting condomless sex with an HIV-positive partner. At the time of study initiation, UVL was not confirmed to protect against HIV transmission, but PrEP guidelines have since been updated to reflect new findings and the very low risk of HIV transmission from a partner with UVL [36, 37]. Furthermore, some versions of the paper-based risk assessment did not specify *condomless* receptive intercourse with a casual partner as a high-risk behaviour, so the comparison to the online behavioural survey was made to any report of receptive intercourse, regardless of condom use.

To our knowledge, *PRELUDE* is the first PrEP demonstration project to target high-HIV risk participants using very specific behavioural eligibility criteria. The baseline characteristics of the enrolled sample reported through an independent online survey validated the use of these criteria in the selection of the cohort, and for general PrEP eligibility in future. Overall, the data demonstrate that clinicians were able to successfully utilise both the paper-based risk assessment and clinical judgement to identify participants who were well-suited to daily PrEP use. Furthermore, the online behavioural survey confirms the findings of the paper risk assessment- that study participants engage in a range of behaviours that placed them at considerable risk of acquiring HIV, and that GBM at high risk of HIV self-select into adopting PrEP. Overall, these findings highlight the need for improved and widespread PrEP access in Australia, where demand is growing [38] and guidelines facilitate the identification of population groups eligible for PrEP. Current NSW guidelines allow some flexibility to ensure that people who need PrEP most are able to gain timely access to it, but it is predominately high-risk GBM that present for and are provided with PrEP in Australia.

## Supporting information

S1 Table Paper-based risk assessment used for enrolment in the *PRELUDE* study. (DOCX)

S1 File *PRELUDE* study protocol. (PDF)

S2 File TREND Statement checklist. (PDF)

S3 File *PRELUDE* baseline behavioural survey. (DOCX)
