# Supplementary material for: Validation of participant eligibility for pre-exposure prophylaxis: Baseline data from the PRELUDE demonstration project
Source: PLoS One. 2017 Sep 26;12(9):e0185398. doi: 10.1371/journal.pone.0185398 (PMC5614574; doi:10.1371/journal.pone.0185398)
Supplement: S1 Table — (DOCX) [file pone.0185398.s001.docx]

**S1 Table: Paper-based risk assessment used for enrolment in the *PRELUDE* study**

1. Are you circumcised?
2. In the last three months, have you had sex with a regular sexual partner who is an HIV-infected man or woman, with whom condoms were not consistently used?
3. In the last three months, have you had any receptive sex with any casual male partner whom you believed to be HIV positive or you did not know his HIV status?
4. (Men only): In the last three months, have you had any insertive sex without a condom where the serostatus of your partner was not known or your partner was HIV positive and not on treatment?
5. In the last three months, have you been diagnosed with rectal gonorrhea or chlamydia?
6. In the last three months, have you used any methamphetamines?
7. In the last three months, have you had more than one episode of anal sex where proper condom use was not achieved (e.g. condoms slipped off or broke)?
8. In the next three months, are you likely to have multiple events of anal or vaginal sex without a condom?
