# Supplementary material for: Validation of participant eligibility for pre-exposure prophylaxis: Baseline data from the PRELUDE demonstration project
Source: PLoS One. 2017 Sep 26;12(9):e0185398. doi: 10.1371/journal.pone.0185398 (PMC5614574; doi:10.1371/journal.pone.0185398)
Supplement: S1 File — (PDF) [file pone.0185398.s002.pdf]

UNSW AUSTRALIA

Never Stand Still | Medicine | The Kirby Institute

| | |
|---|---|
| **Protocol title:** | PRELUDE: Implementation of HIV preexposure prophylaxis with antiretroviral medications among people at high risk for HIV infection: A demonstration project |
| **Protocol number:** | HEPP 1403 |
| **HREC Reference Number:** | HREC/14/SVH/130 |
| **ClinicalTrails.gov Identifier:** | NCT02206555 |
| **Protocol version:** | 4.0 |
| **Protocol date:** | 30 September 2015 |
| **Amendment date:** | 30 September 2015 |
| **Amendment version:** | Protocol amendment 3 (HREC amdt 7) |
| **Name of sponsor:** | The Kirby Institute, The University of New South Wales |

**Coordinating Principal Investigator (CPI):** Dr Iryna Zablotska

Kirby Institute

Table of Contents


1.0 Background and rationale ... 16
1.1 Rationale ... 16
1.2 Name and description of the drug therapy being investigated ... 16
1.3 Summary of relevant clinical trial data from earlier studies ... 17
1.4 Summary of the known potential risks and benefits of TRUVADA® ... 20
1.5 The route of administration, dosage, dosage regimen and treatment period ... 21
1.6 Study background ... 21
2.0 Study aim and objectives ... 24
2.1 Study aim ... 24
2.2 Primary Objective(s) ... 24
2.3 Exploratory objective(s) ... 24
3.0 Study design ... 25
3.1 Summary of study design ... 25
3.2 Number of participants ... 25
3.3 Target population ... 25
4.0 Recruitment and retention of participants ... 27
4.1 Eligibility criteria ... 27
4.2 Recruitment Process ... 28
4.3 Participant Retention ... 28
4.4 Participant Withdrawal ... 29
5.0 Study endpoints ... 30
5.1 Primary endpoints ... 30
5.2 Exploratory endpoints ... 30
6.0 Treatment of participants ... 32
6.1 Treatment ... 32
6.2 Contraindications ... 32
6.3 Warnings, Precautions and Management of Drug Side-effects and Toxicities ... 32
6.3.1 Lactic Acidosis/Severe Hepatomegaly with Steatosis ... 33
6.3.2 HBV and HCV Infection ... 33
6.3.3 New Onset or Worsening Renal Impairment ... 33
6.3.4 Bone Health ... 34
6.3.5 Co-administration with Other Products ... 35
6.3.6 Management of other events ... 35
6.4 Comprehensive Management to Reduce the Risk of Acquiring HIV-1 ... 36
6.5 Treatment interruption and discontinuation ... 36
6.6 Pregnancy and breastfeeding ... 37
6.7 Post-exposure prophylaxis (PEP) ... 38
6.8 Treatment after HIV infection ... 39

7.0 Study assessments and follow-up procedures ..... 40
7.1 Screening and enrolment ..... 40
7.1.1 Pre-Screening ..... 40
7.1.2 Informed consent process ..... 40
7.1.3 Screening visit ..... 41
7.1.4 Enrolment/Baseline visit ..... 42
7.2 Follow-up visits of participants on PrEP ..... 44
7.2.1 Follow-up visit 1 (1 month after enrolment/recommencement) ..... 44
7.2.2 Follow-up visit 2 (3 month since enrollment) ..... 45
7.2.3 Follow-up visits 3 and 4 (6 and 9 months since enrollment) ..... 46
7.2.4 Follow-up visits 5 to12 (12-30 months since enrollment) ..... 47
7.2.5 Interim Visits ..... 48
7.3 Follow-up of patients after discontinuing PrEP ..... 48
7.3.1 Follow-up visit 12 (33 months in the study) ..... 48
7.3.2 Follow-up 13 (36 months in the study) ..... 49
7.4 HIV Counseling and Testing ..... 51
7.4.1 Care for Participants Identified as HIV-Positive ..... 51
8.0 Recording and reporting Adverse Events (AEs) ..... 52
8.1 Adverse Event definitions ..... 52
8.2 Adverse Event assessments ..... 53
8.3 Adverse Event Reporting Requirements ..... 54
8.3.1 Reporting Requirements for sites ..... 54
8.3.2 Reporting requirements for the Sponsor ..... 56
9.0 Packaging, labeling, storage and accountability of clinical study supplies ..... 57
9.1 Study Drug Accountability ..... 57
9.2 Study Drug Dispensing ..... 57
9.3 Study drug return/destruction ..... 58
10.0 Biological samples ..... 59
10.1 Laboratory supplies and sample processing ..... 59
10.1.1 PBMC, plasma and serum sample collection ..... 59
11.0 Data collection, source documents and record retention ..... 61
11.1 Data collection and source documents ..... 61
11.2 Record Retention ..... 62
12.0 Confidentiality of data ..... 63
12.1 Confidentiality of participant records ..... 63
13.0 Analyses and statistical considerations ..... 64
13.1 Outcome measures ..... 64
13.2 Laboratory analyses ..... 64
13.3 Sample size ..... 64
13.4 Statistical analyses ..... 64

13.5 Interim monitoring and analysis ... 65
14.0 Ethics committee/regulatory approval and informed consent ... 66
14.1 Ethics committee/regulatory approval ... 66
14.2 Informed consent ... 66
15.0 Legal Issues ... 67
16.0 Governance ... 68
17.0 Financing and insurance ... 69
18.0 Quality Control (QC) and Quality Assurance (QA) ... 70
18.1 Quality control ... 70
18.2 Protocol deviation and Violation ... 70
19.0 Study oversight and management committees ... 71
19.1 Study Management Team ... 71
19.2 Protocol Steering Committee ... 71
19.3 Data Safety and Monitoring Board ... 71
19.4 Medical Officer ... 72
20.0 Publication Policy ... 73
21.0 References ... 75
22.0 Attachments/Appendices ... 78

## Protocol Synopsis

<table>
<tr><td>Title</td><td>The PRELUDE Study: Implementation of HIV preexposure prophylaxis among people at high risk for HIV infection: A demonstration project</td></tr>
<tr><td>Background and rationale</td><td>Significant increases in HIV diagnoses among gay and other homosexually active men, in Australia and internationally, have been observed since the late 1990s. The levels of high HIV risk sexual practices among gay men have also increased, particularly unprotected anal intercourse (UAI). Nationally, over three quarters of the new HIV infections diagnosed annually are among men who have sex with men (MSM). The proportion of heterosexual men and women among those diagnosed with HIV annually has also increased in recent years. Despite successes in some situations, HIV transmission has not been adequately reduced by the prevention methods available to those at risk, such as education, condoms, and treatment of sexually transmitted infections (STIs).<br><br>The effectiveness of daily oral antiretroviral medications (ARVs) as preexposure prophylaxis of HIV (PrEP) has been established in clinical trials and observational studies, in both heterosexual adults and homosexual men. Through its “HIV prevention strategy 2012-2015: New era,” NSW Health committed to consider how to most appropriately and efficiently implement PrEP in line with evidence. This commitment translated in the support to this PrEP demonstration project.<br><br>This demonstration project is designed to develop and evaluate the model of services providing daily combination of tenofovir disoproxil fumarate and emtricitabine (TDF/FTC, known as TRUVADA®, Gilead Sciences) as PrEP to a sample of sero-negative individuals at high risk for HIV infection in clinical settings in New South Wales, Australia. The project will inform policy development regarding primary HIV prevention with PrEP.</td></tr>
<tr><td>Study objectives</td><td>Aim: To evaluate the implementation of evidence-based provision of PrEP in health care setting in NSW to a sample of individuals at high risk for HIV.<br><br>Primary objective(s)<br><br>1. To assess the feasibility of the process of PrEP delivery in various health care settings in NSW (including eligibility screening; counselling about PrEP, condom use and risk reduction; testing for HIV; prescription and follow-up patients on PrEP).<br>2. To assess the acceptability of PrEP among adults invited to participate in the PrEP implementation project (including overall uptake of PrEP among those offered PrEP and reasons for declining PrEP, patterns of PrEP use among participants, self-reported preferences for alternative schedules and/or duration of PrEP use).<br>3. To assess factors associated with PrEP use:<br>a. adherence to PrEP among HIV negative individuals: levels (measured by behavioural measures), patterns of adherence, and factors associated with optimal and sub-optimal adherence.<br>b. Experience and perceptions of side effects associated with PrEP use (measured by self-report, clinical and laboratory diagnoses) and their impact on PrEP usage and adherence.<br>c. behavioural effects of PrEP use (measured as change in risk behaviours and risk-reduction practices associated with sexual transmission of HIV).</td></tr>
</table>

| | |
|---|---|
| | **Exploratory objective(s)**<br><br>1. To document the ***cost*** of PrEP provision in NSW health care setting; assess the feasibility, funding and potential co-payment schemes in the likelihood of PrEP provision after the completion of this demonstration project.<br>2. Assess biological markers of adherence to the study product and compare them with self-reported adherence. |
| Participant population | Homosexual men and heterosexual men and women at high risk of HIV infection |
| Study design | This is an open-label, single-arm treatment evaluation study.<br><br>**Stage I:** Initially, the study will recruit participants in four large HIV and sexual health services in NSW which currently provide ARVs as treatment and as non-occupational post-exposure prophylaxis of HIV. These will include:<br>• RPA Sexual Health (RPASH)<br>• St. Vincent's Hospital (SVH)<br>• Sydney Sexual Health Centre (SSHC)<br>• Western Sydney Sexual Health Centre (WSSH)<br><br>**Stage II:** As recruitment evolves, the involvement of other health providers (including other public clinics and s100 prescribers) in NSW will be considered. Based on the speed of study start-up and enrolment at the Stage I clinics, Stage II may begin and run concurrently with Stage I. These will include:<br>• Clinic 16 (C16)<br>• Holdsworth House Medical Practice (HH)<br>• Pacific Clinic (PC)<br>Taylor Square Private Clinic (TSPC)<br>***A***pproximately 300 participants will be enrolled and will take daily PrEP for up to 30 months (2.5 years), with subsequent post-PrEP follow-up for additional 6 months. A subset of approximately 100 participants (recruited in three study clinics Sydney Sexual Helath, St Vincent's hospital and Holdsworth House) will participate in in the blood sample collection and analyses. |
| Treatment of participants | All consenting and eligible HIV negative participants will receive a fixed-dose co-formulation of FTC and TDF (i.e., TRUVADA) prescribed for daily administration orally. Each FTC/TDF tablet contains 200 mg of FTC and 300 mg of TDF (equivalent to 245 mg of tenofovir disoproxil). The study product will be supplied by Gilead Sciences (Foster City, USA). |
| Study procedures | Summary:<br>• Clinicians in participating clinics will provide potential PrEP candidates with information on PrEP, and interested patients will have an opportunity to discuss the information about the study with a member of the study team and to ask for any clarification, either during or after this routine visit.<br>• Participants who are interested in PrEP and willing to take part in the PrEP demonstration study will be given a copy of the Participant Information Sheet and Consent Form (PICF) and a verbal explanation of the study purpose and procedures. Written informed consent will be obtained before the screening visit which will include the assessment of knowledge regarding HIV risk and risk reduction and understanding of PrEP, evaluation of client's behavioural eligibility for PrEP, standard of care procedures including confirmation of HIV negative status, testing for sexually transmitted infections (STI) and treatment if necessary, testing for hepatitis B status and vaccination if negative, and |

| | |
|---|---|
| | assessment of hepatic and renal function.<br>• The participant will be enrolled into the study after the informed consent process has been completed and the participant has met all selection criteria. The participant will receive a unique study number, and this will be documented in the participant's medical record and on all study documents. Participants who commit to taking daily PrEP and participating in follow-up visits will be given a prescription for TRUVADA® to cover the period until the next follow-up visit.<br>• At each follow-up visit, the following procedures will be conducted: clinical evaluations/ procedures (performed by a licensed clinician), testing for HIV, STIs, hepatic and renal function, assessment for adherence to the prescribed medication, side-effects, eligibility for TRUVADA® prescription until the next follow-up visit and willingness to continue on PrEP. Collection of plasma and PBMCs for TRUVADA® drug levels and collection of serum for storage will occur at one, six and twelve months after PrEP initiation in participating clinics.<br>• In November 2015, all existing participants who originally consented to take the study medication TRUVADA® for 12 months will be offered to continue taking TRUVADA® for an additional 18 months. All participants willing to take this offer will be asked to update their consent. If they consent to participate in the extension period, the same study procedures will be followed until final TRUVADA® follow-up at month 30.<br>• As the study requirement, participants will complete a self-administered attitude, behavioural and lifestyle survey (accessed online via a desktop or laptop) which will be part of enrolment and each follow-up round and will be completed in the participant's private space.<br>• Any extended prescription of TRUVADA® will require reassessment of eligibility for PrEP, willingness to continue taking it and ability to take part in the next follow-up visit. Reinforcement of the need for risk reduction and condom use will be conducted with each participant at each study visit. Participants discontinuing PrEP will undergo a discussion of risk reduction as well as testing for HIV/STI in the period following discontinuation.<br>• All participants will be asked to complete a self-administered attitude, behavioural and lifestyle survey online via a desktop or laptop at 3 and 6 months following PrEP discontinuation, and to provide consent for data linkage with the HIV registry of NSW, to maximize detection of all HIV infections linked to the use of PrEP. |
| Statistics | The analyses will be described in detail in a full Statistical Analysis Plan. Briefly, they will cover:<br><br>***The feasibility of PrEP delivery:*** Analyses will cover: 1) the acceptance and rejection rates among prospective participants who are offered to participate in the implementation study, along with the reasons for and factors associated with the acceptance of offer or its rejection; 2) eligibility for PrEP among individuals entering the screening process; 3) the proportion of participants providing their consent to participate in the study among individuals found to be eligible, and 4) the retention rate among participants enrolled in the study.<br><br>***Adherence to the study medication:*** Analyses will focus on assessing time on daily TRUVADA® until its discontinuation, number and proportion of doses taken and missed, the relationship between sexual practices and medication use, and the relationship between adherence levels and HIV-free time on the study product.<br><br>***Safety and tolerability***: Analyses will cover the incidence and severity of side effects |

| | |
|---|---|
| | associated with the daily use of TRUVADA, and the associated discontinuation of the study medication.<br><br>***The effects of PrEP use on behaviour:*** Analyses will focus on the prevalence and changes over time in the awareness and perception of HIV risk, sexual practices, condom use, and use of safer sex practices. Comparison of sexual practices over time will cover periods of three months before starting the preventative treatment, during the course of treatment and up to six months after the treatment discontinuation.<br><br>Data collected by this study will also be used to conduct statistical analyses of ***the risk of HIV seroconversion*** among individuals on PrEP with a focus on estimating the risk of seroconversion per person-time of follow-up on TRUVADA, the probability of HIV seroconversion per sexual contact among individuals taking PrEP and the effect modifying role of non-adherence to the prescribed medication schedule, STI and side-effects leading to inconsistent PrEP use. Analyses of HIV seroconversions will also include the period of up to six months after the treatment discontinuation using data linkage with the NSW HIV Registry.<br><br>Analyses will be conducted using appropriate longitudinal regression models with time-varying exposures. Data analysis will be conducted at the Kirby Institute using STATA (StataCorp, College Station, TX, USA). |

**Study Flow Chart** (for details, see table 2)

| Data collection round | Screening Visit 0 | Baseline Visit 1 | Follow-up1 Visit 2 | Follow-up2 Visit 3 | Follow-up3 Visit 4 | Follow-up4 Visit 5 | Follow-up5 Visit 6 | Follow-up 6 to 10 Visit 7-11 | Follow-up 11 Visit 12 | Post-PrEP assessment Visit 13 | Data collection Visit 14 |
|---|---|---|---|---|---|---|---|---|---|---|---|
| Timeline | Week –2 | 0 | Month 1 | Month 3 | Month 6 | Month 9 | Month 12 | Month 15 -27 | Month 30 or discontinuation | Month 33 | Month 36 |
| Informed consent | X | | | | | | X[e] | | | | |
| Review eligibility | X | X | X | X | X | X | X | X | | | |
| Medical history | X | X | | | | | | | | | |
| Interim history / adverse events | | | X | X | X | X | X | X | X | X | |
| Laboratory tests | X | X | X[a] | X | X[a] | X | X[a] | X | X | | |
| Dispense study medication | | X | X | X | X | X | X | X | X | | |
| STI testing [b] | X[c] | X | | X | X | X | X | X | X | X | |
| HIV testing | X | X | X | X | X | X | X | X | X | X[d] | X[d] |
| Attitude, behavioural & lifestyle survey | X | X | X | X | X | X | X | X | X | X | X |

Visits 0 and 1 may be combined.

[a] blood collected for plasma/PBMCs/serum [b] obtained per standard of care [c] Hepatitis B status obtained prior to baseline

[d] HIV status post-PrEP discontinuation may be obtained via data linkage [e] Participants will be re-consented to the extension part of the study at visit 6 if they want to participate in the extension part of the study.

# Protocol team roster

**Study Sponsor**

**The Kirby Institute**
Wallace Wurth Building
UNSW Australia
Sydney NSW 2052
Australia

**Coordinating Principal Investigator (CPI) and Protocol Chair**

**Dr Iryna Zablotska-Manos**
The Kirby Institute
Wallace Wurth Building
UNSW Australia
Sydney NSW 2052
Tel: 61 2 9385 0951
izablotska@kirby.unsw.edu.au

**Co-investigators**

**Prof Andrew Grulich**
The Kirby Institute
Wallace Wurth Building
UNSW Australia
Sydney NSW 2052
Australia
Tel: 61 2 9385 0900
agrulich@kirby.unsw.edu.au

**A/Prof Rebecca Guy**
The Kirby Institute
Wallace Wurth Building
UNSW Australia
Sydney NSW 2052
Australia
Tel: 61 2 9385 0900
rguy@kirby.unsw.edu.au

**Prof John Kaldor**
The Kirby Institute
Wallace Wurth Building
UNSW Australia
Sydney NSW 2052
Australia
Tel: 61 2 9385 0900
Jkaldor@kirby.unsw.edu.au

**Dr Mary Poynten**
The Kirby Institute
Wallace Wurth Building
UNSW Australia
Sydney NSW 2052
Australia
Tel: 61 2 9385 0900
mpoynten@kirby.unsw.edu.au

**A/Prof Garrett Prestage**
The Kirby Institute
Wallace Wurth Building
UNSW Australia
Sydney NSW 2052
Australia
Tel: 61 2 9385 0900
gprestage@kirby.unsw.edu.au

**Clinical Investigators:**

**A/Prof Anna McNulty**
Sydney Sexual Health Centre
Level 3, Nightingale Wing
Sydney Hospital
Macquarie Street
Sydney NSW 2000
Australia
Tel: 61 2 9382 7440
Anna.McNulty@sesiahs.health.nsw.gov.au

**Prof Andrew Carr**
St Vincent's Hospital
HIV, Immunology and Infectious Disease Unit
390 Victoria Street
Darlinghurst NSW 2010
Australia
Tel: 61 2 8382 3359
acarr@stvincents.com.au

**A/Prof David Templeton**
RPA Sexual Health
Royal Prince Alfred Hospital
16 Marsden Street
Camperdown NSW 2050
Australia
Tel: 61 2 9515 1200
david.templeton@email.cs.nsw.gov.au

**Dr Catriona Ooi**
Western Sydney Sexual Health Centre
Level 1, Jeffery House, 162 Marsden Street
Parramatta NSW 2150
Australia
Tel: 61 2 9843 3124
Catriona.ooi@health.nsw.gov.au

Dr Nathan Ryder
Newcastle Community Health Centre
Level 2, 670 Hunter Street
Newcastle NSW 2300
Australia
Tel: 61 2 4016 4536
Nathan.Ryder@hnehealth.nsw.gov.au

Dr Mark Bloch
Holdsworth House Medical Practice
Level 3, 26 College Street,
Sydney NSW 2010
Australia
Tel: 61 2 8038
Mark.Bloch@holdsworthhouse.com.au

Dr Rosalind Foster
Clinic 16
Royal North Shore Hospital , Pacific Hwy
St Leonards NSW 2065
Australia
Tel: 61 2 9901 3530
rosalind.foster@health.nsw.gov.au

Dr Cathy Pell
Taylor Square Private Clinic
393 Bourke Street
Darlinghurst, NSW 2010
Australia
Tel: 61 2 9331 6151
cpell@tspc.com.au

**Other Investigators:**

**Mr Brent Mackie**
AIDS Council of New South Wales (ACON)
414 Elizabeth Street
Sydney NSW 2010
Tel: 61 2 9206 2120
bmackie@acon.org.au

**A/Prof Martin Holt**
Centre for Social Research in Health
University of New South Wales
Room 326, John Goodsell Building
Sydney NSW 2052
Tel: 61 2 9385 6410
m.holt@unsw.edu.au

**Mr John McAllister, RN**
Immunology and Infectious Diseases Ambulatory Care Clinic
Level 4 Xavier Building
St Vincent's Hospital
Darlinghurst NSW 2010
Tel: 61 2 8382 2576
jmcallister@stvincents.com.au

**Prof Kenneth Mayer, MD**
Medical Research Director
The Fenway Institute

1340 Boylston Street
Boston, MA 02215 USA
Tel: 1-401-793-4711
Kenneth_mayer@brown.edu

**Mr Dean Murphy**
Australian Federation of AIDS Organisations
And Centre for Social Research in Health
University of New South Wales
Room 324 John Goodsell Building
Sydney NSW 2052
Tel: 61 2 9385 6946
d.murphy@unsw.edu.au

**Prof John de Wit**
Centre for Social Research in Health
University of New South Wales
Room 315, John Goodsell Building
Sydney NSW 2052
Tel: 61 2 9385 6799
j.dewit@unsw.edu.au

**A/Prof Edwina Wright**
Department of Infectious Diseases
Alfred Hospital, Monash University and Centre for Biomedical Research, Burnet Institute
2nd Floor, Burnet Institute
Commercial Road
Melbourne VIC 3000
Tel: 61 3 9276 6078
E.Wright@alfred.org.au

**Medical Officer**

**A/Prof Mark Boyd, MD**
The Kirby Institute
Wallace Wurth Building
UNSW Australia
Sydney NSW 2052
Australia
Tel: 61 2 9385 0985
mboyd@kirby.unsw.edu.au

Back-up Medical Officer

**Dr Gail Matthews**
The Kirby Institute
Wallace Wurth Building
UNSW Australia
Sydney NSW 2052
Australia
Tel: 61 2 9385 0877
gmatthews@kirby.unsw.edu.au

**Laboratories**

**Plasma and PBMC preparation; serum storage**

Kate Merlin
Supervising Scientist – Clinical Trials
St Vincent's Centre for Applied Medical Research
Lowy-Packer Building, Level 9
405 Liverpool Street
Darlinghurst, NSW 2010
Tel: 61 2 8382 4942
k.mcghie@amr.org.au

**Plasma and PBMC processing**

Craig Hendrix MD
Professor of Medicine
Johns Hopkins University Division of Clinical Pharmacology
Clinical Pharmacology Analytical Laboratory
Blalock 569
600 N. Wolfe St.
Baltimore, MD 21287 USA
Tel: 1 410 955 9707
chendrix@jhmi.edu

**Sample courier service**

Catherine Clarke
Clinical Trials Coordinator
St Vincent's Hospital
Level 6 Xavier Building
Darlinghurst NSW 2010
Tel: 61 2 8382 9125

## Common Acronyms

| Abbreviation/Acronym | Description |
|---|---|
| AE | Adverse Event |
| AIDS | Acquired Immune Deficiency Syndrome |
| ART | Antiretroviral therapy (for HIV positive individuals) |
| ARTG | Australian Register of Therapeutic Goods |
| ARV | Antiretroviral medications |
| BMD | Bone Mineral Density |
| CDC | US Centers for Disease Control and Prevention |
| CRF | Case Report Form |
| CTA | Clinical Trials Authorisation |
| DAIDS | Division of AIDS |
| DSMB | Data Safety and Monitoring Board |
| DSPH | Gilead Drug Safety and Public Health |
| eCRF | electronic Case Report Form |
| eGFR | estimated Glomerular Filtration Rate |
| FDA | U.S. Food and Drug Administration |
| FTC | emtricitabine |
| FU | Follow-Up |
| GCP | Good Clinical Practice |
| HBV | Hepatitis B Virus |
| HIV | Human Immunodeficiency Virus |
| HREC | Human Research Ethics Committee |
| ICH | International Conference on Harmonisation of Technical Requirements for Registration of Pharmaceuticals for Human Use |
| iPrEx study | The international multisite Pre-exposure Prophylaxis Initiative |
| MSM | Men who have Sex with Men |
| NSAID | non-steroidal anti-inflammatory drug |
| PBMC | Peripheral blood mononuclear cell |
| PEP | Post-exposure prophylaxis |
| PICF | Participant Information and Consent Form |
| PrEP | Preexposure prophylaxis |
| PSC | Protocol Steering Committee |
| QA | Quality Assurance |
| QC | Quality Control |
| RAI | Receptive Anal Intercourse |
| SAE | Serious Adverse Event |
| SAR | Serious Adverse Reaction |
| SOP | Standard Operating Procedure |
| STI | Sexually Transmitted Infection |
| SUSAR | Suspected Unexpected Serious Adverse Reaction |
| TDF | tenofovir disoproxil fumarate |
| UADR | unexpected adverse drug reaction |
| UAI | unprotected anal intercourse |

# 1.0 Background and rationale

## 1.1 Rationale

The effectiveness of antiretroviral medications (ARVs) as preexposure prophylaxis of HIV (PrEP) has been established by a number of clinical trials and observational studies conducted in heterosexual adults, homosexual men and people who inject drugs. [1-7] To date, TRUVADA® remains the only antiretroviral medication that has been approved by the US Food and Drug Administration (FDA) for preventative use as PrEP among individuals at high risk for HIV. It has not been licensed for this purpose in Australia yet. However, through the NSW HIV strategy 2012-15, NSW Health has committed "to consider how to most appropriately and efficiently implement PrEP in line with the evidence,"[8] and has provided support to establish and conduct the PrEP demonstration project in NSW.

This demonstration project is designed to evaluate the *off-label provision* of daily combination of tenofovir disoproxil fumarate and emtricitabine (TDF/FTC, marketed as TRUVADA® by *Gilead Sciences*) as PrEP to seronegative adults at high risk for HIV infection in NSW. In light of the NSW HIV Strategy, which commits to the evaluation of mechanisms of PrEP implementation, the project will inform policy development regarding primary HIV prevention with PrEP in NSW and Australia.

## 1.2 Name and description of the drug therapy being investigated

The combination of the nucleoside analogue HIV-1 reverse transcriptase inhibitors tenofovir disoproxil fumarate (TDF) and emtricitabine (FTC) (marketed as TRUVADA®) is produced and distributed by Gilead Sciences, Inc. (Foster City, CA, USA; Medication Guide #21-752-GS-028[9]).

TRUVADA® was initially approved by the U.S. Food and Drug Administration (FDA) in 2004 and indicated, in combination with other antiretroviral agents, for the treatment of HIV-1 infection in adults and paediatric patients 12 years of age and older. The recommended dose of TRUVADA® in adults and in paediatric patients 12 years of age and older with body weight greater than or equal to 35 kg is one tablet (containing 300 mg of TDF and 200 mg of FTC) once daily taken orally. In order to optimise the absorption of tenofovir, it is recommended that TRUVADA® should be taken with food.

Following oral administration, FTC is rapidly absorbed with peak plasma concentrations occurring at 1-2 hours post dose, and the half-life of one dose is approximately 10 hours. [9] Maximum TDF serum concentrations are achieved in 1.0 $\pm$ 0.4 hour, with half-life of approximately 17 hours. TDF is principally eliminated by the kidney, and FTC – by kidney and digestive tract. Although, TDF alone has been proven to significantly reduce the risk of HIV infection, FTC complements TDF in establishing protective levels earlier and reaching rectum faster. Therefore, a combination of TDF and FTC is more favourable, particularly for homosexual men, than TDF alone.

Based on the results of clinical trials in men who have sex with men (MSM) at high risk for HIV-1 infection and in heterosexual serodiscordant couples (See Clinical Studies in section 1.3), TRUVADA® (in combination with safer sex practices) was subsequently approved by the FDA as HIV Preexposure Prophylaxis (PrEP) in high HIV risk adults in July 2012.[10] TRUVADA® is the only agent to be approved for HIV prevention in uninfected adults.

## 1.3 Summary of relevant clinical trial data from earlier studies

In the past few years there have been major advances in the field of biomedical prevention of sexually transmitted HIV. As of September 2015, data from ten placebo-controlled randomized clinical trials (RCTs) and three observational studies confirm that TDF alone or in combination with FTC (as TRUVADA®) is effective when used as oral preexposure prophylaxis of HIV infection in men who have sex with men [1,11], heterosexual adults at high risk for HIV infection [2] and people injecting drugs [3]. Overall, the pooled risk ratio from these studies is in favour of PrEP use (pooled risk ratio (PRR)=0.49; 95% confidence interval (95% CI): 0.33-0.73 [WHO meta-analysis, under review for Cochrane Database Syst Rev). In the same pooled analysis, PrEP has been found to be effective in reducing the risk of HIV infection across gender, PrEP regimen (TDF alone or a combination of TDF and FTC (TRUVADA®), dosing (daily or intermittent), and mode of HIV acquisition (sexual versus percutaneous exposure).

TDF alone, or in combination with FTC has been the oral ARV of choice in clinical trials, primarily due to its long half-life, favourable safety profile, and effective penetration into the genital compartment. [12-14]

The international multisite Pre-exposure Prophylaxis Initiative (iPrEx study) specifically investigated safety and efficacy in high risk MSM populations [1] and demonstrated that once-daily TDF/FTC decreased the likelihood of HIV infection by 42% among 2499 high-risk MSM. Adherence to study medications was crucially linked to efficacy: those participants who took at least 90% of study drug had a greater than 70% level of protection and men with detectable levels of TDF or FTC in plasma and peripheral blood mononuclear cells experienced a greater than 90% protective effect. [15]

More recently, evidence from the iPERGAY and PROUD studies suggests that in high risk individuals PrEP may be more effective than previously thought. Both studies enrolled participants with very high expected HIV incidence and, despite differences in TRUVADA® dosing, showed the same 86% reduction in HIV risk due to PrEP. [6,7]

In heterosexual men and women, in two studies daily oral TDF/FTC use has been shown to be safe in reducing the risk for heterosexual HIV acquisition when consistently used. The Partners PrEP trial reported 75% efficacy for TDF/FTC and 67% efficacy for TDF alone. The TDF2 trial found 62% efficacy in men and women, with 84% medication adherence by returned pill count. However, two other studies with heterosexual women (FEM-PrEP trial and the VOICE trial) did not find PrEP to be effective and reported very low levels of medication adherence. The conflicting trial results for efficacy of TDF/FTC in heterosexual men and women can be explained in part by the poor medication adherence.

Adherence appears to pose a major issue for effective implementation of PrEP on population level. [16] Published evidence from clinical trials clearly suggests a wide gap between the average reduction in the HIV risk provided by ARVs and their adherence-adjusted efficacy. [17] This evidence is consistent throughout different population groups, including MSM and transgender people, heterosexual men and women, and people who inject drugs (see Table 1 and Figure 1). On average, TRUVADA® efficacy levels were moderate and ranged from 42% among homosexual men [1] to 72% among heterosexuals.[18] In the same studies, adherence-adjusted efficacy measured by TDF detection in blood rose significantly to 92% in homosexual men and 84% in heterosexuals. On the other hand, the FEM-PrEP[19] and VOICE[20] trials, where participating women were not able to adhere to the same medication, were stopped for futility. The observed gap between average and adherence-adjusted levels of protection appears to vary not only across studies, but also across countries and research

sites. This is best illustrated by the iPrEx study, which found better adherence to PrEP among homosexual men in the US as opposed to the study sites in other countries. [1]

Studies have also found lower adherence rates in study groups taking intermittent compared to daily PrEP, despite many participants expressing a preference for intermittent dosing. [21-23] It appears that daily PrEP schedule may facilitate the development of pill taking skills by otherwise healthy individuals, make decision-making about taking pills easier (as it is not dependent on the need to predict a risk event), may be easier to manage in cases of missed pills, and may also simplify the clinician-client communication about risk events and PrEP use.

Adherence to daily PrEP was shown to be higher in individuals who are well informed about HIV risk and PrEP and with higher attained education level (iPrEx study). [24] Users at high risk for HIV infection and motivated to stay negative are also more likely to adhere to PrEP (iPrEx-OLE study). [5]

.

Higher levels of adherence can be expected among self-selected, motivated PrEP users and among those who are better informed about PrEP and HIV prevention, as recorded by iPrEx study and its open-label extension.[25]

**Table 1: Results from randomized, placebo-controlled, clinical trials of the efficacy of daily oral antiretroviral preexposure prophylaxis (PrEP) for preventing human immunodeficiency virus (HIV) infection** [17]

| Clinical trial | Participants | Type of medication | mITT efficacy* | | Adherence-adjusted efficacy based on TDF detection in blood | |
|---|---|---|---|---|---|---|
| | | | % | (95% CI) | % | (95% CI) |
| **Bangkok Tenofovir Study** | Injecting drug users | TDF | 49 | (10–72) | 70 | (2–91) |
| **Partners PrEP** | HIV discordant couples | TDF | 67 | (44–81) | 86 | (67–94) |
| | | TDF/FTC | 75 | (55–87) | 90 | (58–98) |
| **TDF2** | Heterosexually active men and women | TDF/FTC | 62 | (22–83) | 84 | NS |
| **iPrEx** | Men who have sex with men | TDF/FTC | 42 | (18–60) | 92 | (40–99) |
| **Fem-PrEP** | Heterosexually active women | TDF/FTC | NS | — | NA | — |
| **VOICE** | Heterosexually active women | TDF | NS | — | NA | — |
| | | TDF/FTC | NS | — | NA | — |

**Table 1 abbreviations:**
CI, confidence interval
FTC, emtricitabine
mITT, modified intent to treat analysis, excluding persons determined to have had HIV infection at enrolment
NA, data not available
NS, not statistically significant
TDF, tenofovir disoproxil fumarate
* % reduction in acquisition of HIV infection

**Figure 1: Efficacy of daily TRUVADA® for prevention of HIV infection: WHO meta-analysis of published evidence. [under review in Cochrane Database Syst Rev]**

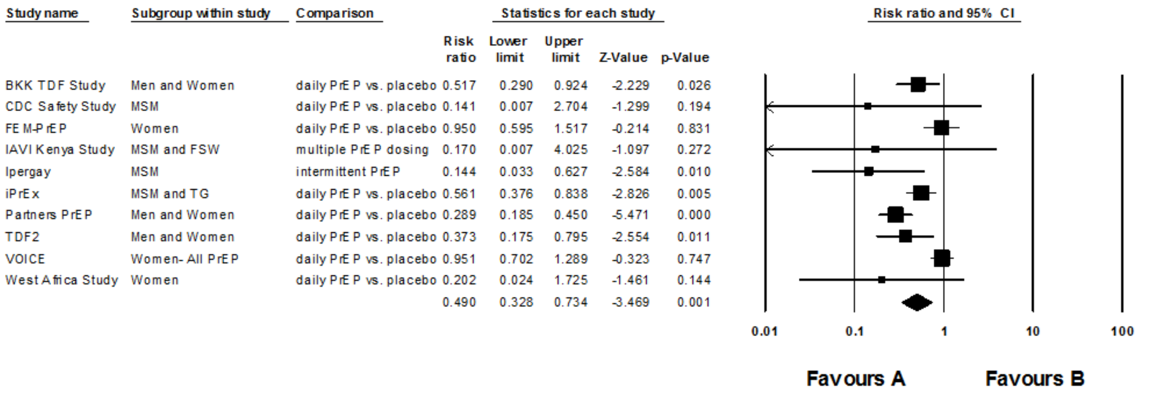


Studies of pharmacokinetics and statistical models of the relationship between drug adherence levels and TDF efficacy in reducing the risk of HIV infection found that adherence of seven doses per

week corresponded to 99% efficacy, four doses per week to 96% and two doses per week to 76% efficacy. [15] However, there are currently no criteria established as to how many missed doses can be considered safe.

Based on the review of available literature on medication adherence, the US CDC does not provide guidance on discontinuing PrEP for reasons of non-adherence, but recommends that healthcare providers work together with patients eligible for and willing to take PrEP to influence and reinforce adherence instructions.

The levels of adherence also vary depending on the adherence measures used. [26] For example, in the iPrEx study daily TRUVADA® use was self-reported by 95% of participants, pill counts adjusted adherence down to 86%, while TDF was detected in only 51% of blood samples from HIV negative participants and 9% of seroconverters. [1]

As to the safety profile of daily TRUVADA, although the regimen was generally well tolerated, participants taking TDF/FTC were more likely to report mild nausea and experience unintentional weight loss in the first few weeks than participants on placebo. There was an upward trend in reversible changes in renal function (2% vs 1%, p=0.08) among participants on TDF/FTC. [1] These clinical findings occurred uncommonly and were either self-limited or responded to temporarily stopping the medication.

As to sexual behaviour, all previous clinical trials promoted condom use and safe sex practices. Overall indices of behavioural risk declined or remained stable during follow-up among men who have sex with men in the iPrEx Study and in its Open Label Extension. Similarly, trials of heterosexual men and women receiving antiretroviral preexposure prophylaxis for HIV prevention found that sexual behaviour remained stable. Among studies which reported the relationship between PrEP use and sexual behaviour, the Partners PrEP Study found a very small but significant effect. In this study, HIV negative participants who were taking PrEP were significantly more likely to have unprotected anal intercourse with outside partners after unmasking but did not change the frequency of unprotected anal intercourse with their HIV positive regular partners. Based on the result, the study investigators concluded that PrEP, provided as part of a comprehensive prevention package, might not result in substantial changes in risk-taking sexual behaviour by heterosexual couples. [27] Only one study among homosexual men has so far reported that decrease in condom use among MSM on PrEP. [28] Given that the effect of PrEP on risk behaviour outside of randomised clinical trials remains unclear, one of the goals of the PRELUDE Study is to investigate the effect of PrEP on risk behaviour during and after the course of PrEP.

In summary, available results from the PrEP studies already strongly suggest that HIV prophylaxis with ARVs is a feasible strategy that can and should be utilised. [15] It should complement existent prevention strategies. However, all evidence strongly suggests that to be effective, PrEP must be taken consistently. PrEP implementation should focus on assessing and supporting adherence as well as identifying and eliminating barriers to consistent PrEP use. Understanding of PrEP implementation barriers, particularly among people taking PrEP outside clinical trials and for longer time than was observed in clinical trials, will be crucial for successful PrEP implementation in the future.

## 1.4 Summary of the known potential risks and benefits of TRUVADA®

For full information on prescribing TRUVADA, see the TRUVADA® Product Information in Appendix I (23 October 2013).

TRUVADA® is indicated:

- In combination with other antiretroviral agents, for the treatment of HIV-1 infection in adults and pediatric patients 12 years of age and older;
- In combination with safer sex practices, for PrEP to reduce the risk of sexually acquired HIV-1 in adults at high risk of getting infected.

TRUVADA® should not be used for PrEP in individuals with unknown or positive HIV-1 status. In HIV-infected patients, it should be used only in combination with other antiretroviral agents.

Adverse reactions among HIV-1 uninfected individuals that were reported by more than 2% of TRUVADA® subjects and more frequently than by placebo subjects were: headache, abdominal pain and weight loss.

Warnings and precautions for HIV-uninfected adults include:

- New onset or worsening renal impairment
- Lactic acidosis and severe hepatomegaly with steatosis, including fatal cases, have been reported with the use of nucleoside analogues
- Decreases in bone mineral density (BMD)
- TRUVADA® should not be co-administration with other products containing emtricitabine or tenofovir disoproxil fumarate including ATRIPLA, EVIPLERA, EMTRIVA, STRIBILD, VIREAD; with drugs containing lamivudine; or with HEPSERA.
- Redistribution/accumulation of body fat
- Severe acute exacerbations of hepatitis B in patients who have discontinued TRUVADA.
- Drug-resistant HIV-1 variants have been identified with the use of TRUVADA® for a PrEP indication following undetected acute HIV-1 infection.
- Comprehensive management including other prevention measures is recommended to reduce the risk of acquiring HIV-1.

## 1.5 The route of administration, dosage, dosage regimen and treatment period

For PrEP purposes, the dose of TRUVADA® in HIV-1 uninfected adults is one tablet (containing 200 mg of emtricitabine and 300 mg of tenofovir disoproxil fumarate) once daily. . In order to optimise the absorption of tenofovir, it is recommended that TRUVADA® should be taken with food. (see the TRUVADA® Product Information in Appendix I ).

The TRUVADA® Product Information does not specify the maximum treatment period, but it would be the period of time during which the person is at high risk of HIV infection.

## 1.6 Study background

***HIV epidemic in Australia:*** Significant increases in HIV diagnoses among gay and other homosexually active men, in Australia and internationally, have been observed since the late 1990s. [2;3] Similar increases were recorded in high HIV risk sexual practices, particularly unprotected anal intercourse (UAI). [4;5] Nationally, over three quarters of the new HIV infections diagnosed annually are among men who have sex with men (MSM). [6] Several factors have been associated with increased HIV

incidence among Australian gay men including: UAI with HIV positive regular partners, receptive UAI with casual partners of HIV positive or unknown status (the risk is also increased during insertive UAI for uncircumcised men), concurrent sexually transmitted infections (STI), particularly rectal infections with gonorrhea or chlamydia, and recent use of methamphetamines (see Appendix II for details). The proportion of heterosexual men and women among those diagnosed with HIV annually has also increased in recent years. [6] Despite successes in some situations, HIV transmission has not been adequately reduced by the prevention methods available to those at risk, such as education, condoms, treatment of STIs and antiretroviral therapy for HIV positive individuals (ART). [7] Therefore, the introduction of new prevention approaches is necessary.

***The emerging use of PrEP in Australia***: Before 2011, there was no reported use of PrEP in Australia. [29] However, in 2011 (immediately following the publication of iPrEx results in late 2010), 2.5% of sexually active HIV negative men recruited by Australian Gay Community Periodic Surveys reported using ARVs as PrEP [30], and similar low levels were observed in 2012 (Zablotska et al, data not published). As to PrEP awareness and willingness to use, by 2007, 43% of the Health in Men (HIM) study respondents in NSW were willing to participate in trials using antiretroviral drugs (ARVs) to prevent HIV infection. [29] In 2011, PrEP acceptability levels were assessed by the Australian PrEPARE survey, which reported that 28% were willing to use PrEP.[31] It appears that while there are no PrEP education and information campaigns in Australia, there is already interest, at least in gay communities, in PrEP as an HIV prevention strategy. [32]

***The role of PrEP in HIV prevention strategy:*** Australia maintains high commitment to reducing rates of HIV infections and recognizes that new technological developments should be considered for HIV prevention. [33] The Melbourne Declaration has pledged to halve infections in Australia by 2015 and embraced a progressive approach of making HIV PrEP available. The NSW HIV strategy aims to work towards the virtual elimination of HIV transmission by 2020, focusing on reducing the HIV transmission among gay and other homosexually active men by 60% by 2015 (80% by 2020) and heterosexual transmission of HIV by 50% by 2015. [8] During the period of this strategy (2012-2015), NSW Health agreed, for the first time, the need to evaluate the mechanisms to most appropriately and efficiently implement PrEP in line with evidence. [8] This study of PrEP implementation in NSW health care settings is designed to establish and evaluate a PrEP implementation model in line with the NSW HIV strategy.

***Policy environment***: At the time when this study was conceived, there were no guidelines for prescribing PrEP in NSW and Australia, beyond a commitment in the NSW HIV Strategy to consider the mechanisms to most appropriately and efficiently implement PrEP in line with evidence. This project was designed to inform PrEP policy development in NSW and Australia. It has contributed to the development of the Interim NSW PrEP guidelines [34] and the National PrEP guidelines in Australia [35]. Both endorse the US Centers for Disease Control and Prevention (CDC) Interim Guidance on Preexposure Prophylaxis for the Prevention of HIV Infection [16] (see Appendix III) and adopt and adapt it for implementation in Australia, among high HIV risk groups (MSM and high risk heterosexual men and women).

A NSW PrEP advisory group has been established to guide the development of the NSW PrEP guidelines. This group has assumed responsibility for developing clinical guidance for the use of HIV PrEP in NSW, including the detailed eligibility criteria, educational materials and counselling considerations and prescription guidance for implementation of PrEP in NSW. The interaction of this project and the advisory body is dual: the project has provided local evidence for policy development and the PrEP advisory group oversees PrEP policy evaluation in NSW.

***Clinical governance:*** TRUVADA® is on the Australian Register of Therapeutic Goods (ARTG) for the treatment of HIV-infected adults in combination with other antiretroviral agents. It has not been

licensed as PrEP in Australia so far, although it has been widely used as post-exposure prophylaxis (PEP) against HIV, with state-based funding for the medication. This study is evaluating an off-label use of TRUVADA, and each participating hospital or clinical practice will have their respective Drug and Therapeutics Committees and the Director of Pharmacy evaluate and approve its use before prescribing TRUVADA.

This study will be conducted in compliance with the protocol, International Conference on Harmonisation of Technical Requirements for Registration of Pharmaceuticals for Human Use (ICH) Good Clinical Practices (GCP) guidelines (including TGA annotated Note for Guidance on Good Clinical Practice), and applicable regulatory requirements.

# 2.0 Study aim and objectives

## 2.1 Study aim

To evaluate the implementation of evidence-based provision of PrEP in health care setting in NSW to a sample of individuals at high risk for HIV.

## 2.2 Primary Objective(s)

1. To assess the ***feasibility of the process*** of PrEP delivery in various health care settings in NSW (including eligibility screening; counselling about PrEP, condom use and risk reduction; testing for HIV; preventive ARV prescription, and follow-up of PrEP patients).

2. To assess the ***acceptability*** of PrEP among adults invited to participate in the PrEP implementation study (including overall uptake of PrEP among those offered PrEP and reasons for declining PrEP, patterns of PrEP use among participants, self-reported preferences for alternative schedules and/or duration of PrEP use).

3. To assess factors associated with PrEP use:
   a) ***adherence*** to PrEP among HIV negative individuals: levels (measured by behavioural measures), patterns of adherence, and factors associated with optimal and sub-optimal adherence.
   b) Experience and perceptions of ***side effects*** associated with PrEP use (measured by self-report, clinical and laboratory diagnoses) and their impact on PrEP usage and adherence.
   c) ***behavioural effects*** of PrEP use (measured as change in risk behaviours and risk-reduction practices associated with sexual transmission of HIV).

## 2.3 Exploratory objective(s)

1. To document the ***cost*** of PrEP provision in NSW health care setting; assess the feasibility, funding and potential co-payment schemes in the likelihood of PrEP provision after the completion of this demonstration project.

2. Assess biological markers of adherence to the study product and compare them with self-reported adherence.

# 3.0 Study design

## 3.1 Summary of study design

This is an open-label, single-arm treatment protocol with prospective data collection.

**Stage I:** During Stage I, the study will recruit participants in four large HIV and sexual health services in NSW which currently provide ARVs as treatment and as non-occupational post-exposure prophylaxis of HIV. These will include:

- St. Vincent's Hospital (SVH)
- Sydney Sexual Health Centre (SSHC)
- RPA Sexual Health (RPASH)
- Western Sydney Sexual Health Centre (WSSH)

**Stage II:** As recruitment evolves, the involvement of other health providers (including other public clinics and s100 prescribers) will be considered. These will include:

- Holdsworth House Medical Practice (HH)
- Taylor Square Private Clinic (TSPC)
- Clinic 16 (C16)
- Pacific Clinic (PC)

Enrolment of participants in Stage I and Stage II clinics may overlap in time.

In June 2015, the PRELUDE investigators team has received an approval from Gilead Sciences for an extended supply of TRUVADA® for additional 18 months (that is a total supply for up to 2.5 years).

All eight clinics, and study participants enrolled in the study in these clinics, will be invited to participate in the study for up to 4.5 years, with total medication follow-up up to 2.5 years. The updated study timeline is presented in Figure 2 below. The number of participants in the study and the study procedures are described in the following sections.

Figure 2 Study timeline

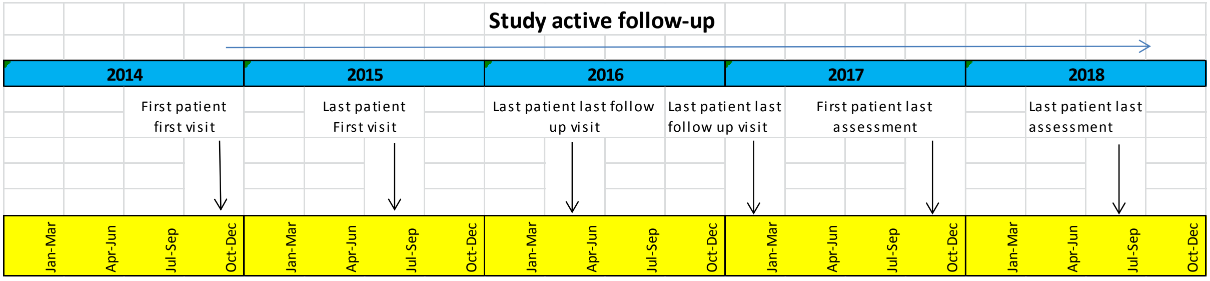


## 3.2 Number of participants

The total number of participants will be approximately 300. It is anticipated that at least 100 participants will be recruited in the Stage-I clinics.

## 3.3 Target population

The *target population* for this demonstration project is *high-risk gay men and high-risk heterosexual men and women*. Sexual transmission of HIV among gay men has been the predominant mode of HIV infection in NSW. [6] Although heterosexual HIV transmission has played a smaller part in the local HIV epidemic, it is important to evaluate PrEP use among heterosexual participants, in particular those in serodiscordant relationships. Thus, heterosexual participants are also included in this project.

# 4.0 Recruitment and retention of participants

## 4.1 Eligibility criteria

This study follows the Interim PrEP guidelines in NSW [34] which endorsedthe US CDC Interim Guidance on Pre-exposure Prophylaxis for the Prevention of HIV Infection [16] and adapted it for identifying Australian individuals at high risk for HIV infection eligible to participate in this study.

The reasons for declining participation among those eligible will be recorded.

**Inclusion criteria:**

- HIV negative at enrollment, with a negative HIV test result documented within seven days of initiating PrEP
- At high and ongoing risk for acquiring HIV infection through sexual exposure (use Algorithm presented in Boxes A and B in Appendix III)
- Aged 18 years or over
- Resident of NSW (or elsewhere in Australia if they visit NSW with sufficient frequency to allow participation)
- Medicare eligible (to have Medicare coverage for the standard-of-care services)
- Willing and able to provide informed consent
- Willing and able to take part in all required study procedures
- Proficiency in written and spoken English (necessary to complete attitude, behavioural and lifestyle surveys)

**Exclusion criteria:**

- HIV-1 infected or has symptoms consistent with acute viral infection (If HIV positive status is not confirmed by testing, delay starting PrEP for at least one month and reconfirm negative HIV-1 status).
- Having an estimated creatinine clearance (glomerular filtration rate [GFR]) <60ml/min
- Having or developing clinical symptoms suggestive of lactic acidosis or pronounced hepatotoxicity (including nausea, vomiting, unusual or unexpected stomach discomfort, and weakness)
- Concurrently taking a nephrotoxic agent (e.g., high-dose non-steroidal anti-inflammatory drugs / NSAIDs)
- Allergic to tenofovir disoproxil fumarate and/or emtricitabine (based on self-report or recorded)
- Concurrently taking prescribed products containing emtricitabine or tenofovir disoproxil fumarate including ATRIPLA®, COMPLERA®, EMTRIVA, STRIBILD®, VIREAD; other drugs containing lamivudine; HEPSERA
- Mental health issues, memory loss or other cognitive impairment or intellectual disability that may compromise participant safety and/or regimen adherence
- Factors or conditions that may compromise a participant's retention in the study (incarceration, planned relocation or potential absence from NSW for a period of 3 months or longer during the course of the study)
- Unwilling to adhere to any of the required study procedures
- Currently breastfeeding

*__Note__: Safety for infants exposed to TRUVADA® during pregnancy is not fully assessed but no harm has been reported. Therefore, planning to become pregnant or currently being pregnant is not an exclusion criterion for this study. However, women who are pregnant should learn about the risks and benefits of TRUVADA® to reduce the risk of acquiring HIV during their pregnancy.*

*Site investigators will review the risks and benefits of TRUVADA® and of potential HIV infection with pregnant women and women who plan to become pregnant.*

## 4.2 Recruitment Process

Prospective participants of this study include high-risk HIV negative adults attending the participating clinics, others referred by the NSW network of s100 providers and those who are self-referred.

Participants of previous studies conducted in NSW who had reported previous experience and/or willingness to take PrEP and expressed interest to participate in PrEP-related research may also be eligible for recruitment and will be referred to clinical sites by the study clinical coordinator.

All relevant clinic staff will be trained to identify and/or refer potentially eligible participants.

Study promotion materials, fliers, recruitment scripts and other locally acceptable methods for recruitment approved by the study's Human Research Ethics Committee may be used at NSW clinics providing sexual health and HIV testing and post-exposure prophylaxis, and elsewhere as needed for targeted recruitment.

## 4.3 Participant Retention

Study site staff, together with the project management team, is responsible for ensuring that high retention rates are met. Components of such strategies may include:

1. Thorough explanation of the study visit schedule and procedural requirements during the informed consent process, and re-emphasis at each study visit
2. Collection of detailed locator information at the screening visit, and active review and updating of this information at each subsequent visit
3. Use of appropriate and timely visit reminder mechanisms
4. Immediate and multifaceted follow-up on missed visits
5. Regular communication with the study participants at large about the progress of the study, awareness about the local HIV epidemic, the purpose of HIV prevention research (including research on biomedical HIV prevention), the importance of HIV risk reduction (including condom use and correct use of PrEP) and the importance of completing research study visits.

It is vital that each site recognize the diversity of the participants in this study, including gay, bisexual and other men who have sex with men and heterosexual adults who are at high risk for HIV infection, and also recognize the importance of interacting with each group in a culturally competent manner.

## 4.4 Participant Withdrawal

Participants may voluntarily withdraw from the study at any time and for any reason. The site investigators also may withdraw participants from the study in order to protect their safety and/or if the participants are unwilling or unable to comply with required study procedures. Participants, who discontinue PrEP for any reason but have had a high risk exposure event in the 72 hours before withdrawing, may require post-exposure prophylaxis (PEP) of HIV. If indicated, they should be transitioned to the standard-of-care PEP course (see Section 6.7 for details).

The study participation of the entire cohort or its part/s may also be terminated if the study sponsor, government or regulatory authorities, the study reviewing ethics committee/s or the site's governance office terminate the study prior to its planned end date.

Every reasonable effort will be made to complete a final evaluation (as described in Section 7.3 below) of those participants who terminate PrEP prior to the anticipated end of their study follow-up. The study staff will record the reason(s) for all withdrawals from the study in participants' study records.

## 5.0 Study endpoints

### 5.1 Primary endpoints

***Feasibility of the process*** of PrEP delivery in health care setting in NSW:

- Accrual of 300 person-years of PrEP
- Seroconversion-free time on PrEP
- Retention rate among participants enrolled in the study

***Acceptability:***

- Among the eligible individuals invited to take part in the PrEP demonstration study, proportion who accept the offer
- Eligibility for PrEP among individuals entering the screening process
- Among individuals found to be eligible to participate in the study, the proportion who provide their consent to participate

A***dherence:***

- Time to TRUVADA® discontinuation
- Number, proportion and patterns of prescribed doses taken and missed (estimated from self-report and dispensing records.

***Safety and side effects****:*

- Incidence of HIV seroconversion among study participants during the course of their study participation and in six months following PrEP discontinuation
- Serious adverse reactions (see Section 8.1 for details)
- Any adverse events, and in particular those leading to interruption or discontinuation of the study product (TRUVADA)

B***ehavioural effects*** of PrEP use:

- Levels of awareness and perception of HIV risk
- Participant self-reported frequency of sexual activity and condom use
- Participant self-reported likelihood of condom use after discontinuation of TRUVADA
- New rectal or vaginal gonorrhoea and chlamydia infections
- Pregnancy

### 5.2 Exploratory endpoints

***Feasibility:***

- feasibility of systems to identify those potentially eligible for PrEP

***Acceptability:***

- Among the eligible individuals invited to take part in the PrEP demonstration study, reasons for not consenting to PrEP, not initiating PrEP or ceasing PrEP despite remaining at high risk
- Participants' self-discontinuation of PrEP due to the issues related to its ease of use and satisfaction; product experiences, and likelihood of willing to access and use PrEP in the future, after the study completion.
- Participant self-reported likelihood of using TRUVADA, dosages and their means of delivery as PrEP after the discontinuation of the demonstration study

***Adherence:***

- Facilitators and barriers to adherence
- Tenofovir and emtricitabine concentrations in blood plasma
- Tenofovir-diphosphate and emtricitabine-triphosphate concentrations in peripheral blood mononuclear cells (PBMCs)
- Correlation between PK and adherence: To assess correlation of PK with adherence measures

***Cost of PrEP provision in NSW health care setting:***

- Cost characterization regarding services, staff, and time needs for administering PrEP
- Cost characterization regarding funding and co-payment needs for administering PrEP after the completion of this demonstration study

  All study end-points will be evaluated during the short-term (12 months) and extended follow-up periods.

## 6.0 Treatment of participants

### 6.1 Treatment

Emtricitabine/tenofovir disoproxil fumarate (FTC/TDF) is a fixed-dose co-formulation of FTC and TDF prescribed for daily administration. Each FTC/TDF tablet contains of 200 mg of FTC and 300 mg of TDF (equivalent to 245 mg of tenofovir disoproxil). The study product will be supplied by Gilead Sciences (Foster City, Ca, USA). More detail about TRUVADA® is provided in the Full Prescribing Information (Appendix I).

Participants will be directed to commence one TRUVADA® tablet orally once daily for at least 30 days. In order to optimise the absorption of tenofovir, it is recommended that TRUVADA® should be taken with food .

If eligibility for and willingness to continue taking PrEP is confirmed at the 30-day follow-up visit, the prescription of the same product can be reissued for two months till Month 3 and up to three months for each subsequent follow-up visit until discontinuation or end of the study. However, only one month's worth of medication will be dispensed at a time. The extension of the medication prescription at any time before the last follow-up visit is subject to continued eligibility for product use based on the study eligibility criteria.

The treatment of the study participants will be considered completed when all follow-up procedures related to treatment are completed and all remaining participants have completed their treatment discontinuation visit.

As per the interim NSW PrEP guidance, access to treatment for participants after the study completion will be discussed with all participants at the treatment discontinuation or last study visit. All HIV negative participants will undergo a discussion of their risk reduction plan at their treatment discontinuation visit or the end of the study.

### 6.2 Contraindications

TRUVADA® is contraindicated for preexposure prophylaxis in individuals with unknown or positive HIV-1 status. TRUVADA® should be used in HIV-infected patients only in combination with other antiretroviral agents. [9]

### 6.3 Warnings, Precautions and Management of Drug Side-effects and Toxicities

Laboratory monitoring and symptom-directed physical exams will be used to detect serious adverse events (SAEs), and any potential reasons for treatment interruption or discontinuation. The site investigator must provide the study participants with medical care that is necessary as a result of any adverse events experienced during or following the study that are related to the study.

A list of expected adverse events and toxicities associated with TRUVADA® is included in Appendix IV. The Full Product/Prescribing Information's Warnings and Precautions along with the management of each per protocol follow.

### 6.3.1 Lactic Acidosis/Severe Hepatomegaly with Steatosis

Lactic acidosis and severe hepatomegaly with steatosis, including fatal cases, have been reported with the use of nucleoside analogs, including VIREAD, a component of TRUVADA, in combination with other antiretrovirals. A majority of these cases have been in women. Obesity and prolonged nucleoside exposure may be risk factors. Particular caution should be exercised when administering nucleoside analogs to any patient or uninfected individual with known risk factors for liver disease; however, cases have also been reported in HIV-1 infected patients with no known risk factors.

***Management per protocol:*** Treatment with TRUVADA® will be suspended in any participant who develops clinical or laboratory findings suggestive of lactic acidosis or pronounced hepatotoxicity (which may include hepatomegaly and steatosis even in the absence of marked transaminase elevations).

### 6.3.2 HBV and HCV Infection

It is recommended that all individuals be tested for the presence of chronic hepatitis B virus (HBV) before initiating TRUVADA. TRUVADA® is not approved for the treatment of chronic HBV infection and the safety and efficacy of TRUVADA® have not been established in patients infected with HBV. Severe acute exacerbations of hepatitis B have been reported in patients who are co-infected with HBV and HIV-1 and have discontinued TRUVADA. In some patients infected with HBV and treated with EMTRIVA, the exacerbations of hepatitis B were associated with liver decompensation and liver failure. It is also recommended that individuals be tested for hepatitis C virus (HCV) depending on risk factors.

***Management per protocol:*** All participants presenting for PrEP will be assessed for HBV infection. At screening, this will include standard-of-care testing and documentation of HBV status before study enrolment, and an assessment of HBV exposure risk over the preceding three months. HBV-uninfected but susceptible individuals should be offered vaccination if not immune, unless they have experienced previous failure to complete a course of vaccination.

Participants who test positive for hepatitis B surface antigen (HBsAg) before enrolment or once enrolled will be closely monitored by either the study clinician or a specialist. In the latter case, the study clinician will be responsible to establish communication with the specialist, and in any case will ensure continuity of treatment for hepatitis B, with both clinical and laboratory follow-up for at least several months after stopping treatment with TRUVADA. These participants will be tested for HBV DNA by the use of a quantitative assay to determine the level of HBV replication at either the six and/or the 12 month visit.

No wash out period is required to transition participants to TRUVADA® from any hepatitis medication listed in the exclusion criterion (e.g. containing emtricitabine or tenofovir disoproxil fumarate including ATRIPLA®, COMPLERA®, EMTRIVA, STRIBILD®, VIREAD; other drugs containing lamivudine; HEPSERA).

Participants will be tested for HCV infection depending on risk factors, and any HCV-positive status must be documented before PrEP initiation.

### 6.3.3 New Onset or Worsening Renal Impairment

Emtricitabine and tenofovir are principally eliminated by the kidney. Renal impairment, including cases of acute renal failure and Fanconi syndrome (renal tubular injury with severe hypophosphatemia), has been reported among HIV-infected individuals with the use of VIREAD; thus, the warning per the Prescribing Information relates to people who have HIV infection. It is recommended that creatinine clearance be calculated in all individuals prior to initiating therapy and as clinically appropriate during therapy with TRUVADA. In patients at risk of renal dysfunction, including patients who have previously experienced renal events while receiving HEPSERA®, it is recommended that estimated creatinine clearance, serum phosphorus, urine glucose, and urine protein be assessed prior to initiation of TRUVADA, and periodically during TRUVADA® therapy.

TRUVADA® should be avoided with concurrent or recent use of a nephrotoxic agent (e.g., high-dose or multiple non-steroidal anti-inflammatory drugs (NSAIDs)). Cases of acute renal failure after initiation of high dose or multiple NSAIDs have been reported in HIV-infected patients with risk factors for renal dysfunction who appeared stable on tenofovir DF. Some patients required hospitalization and renal replacement therapy. Alternatives to NSAIDs should be considered, if needed, in patients at risk for renal dysfunction.

Persistent or worsening bone pain, pain in extremities, fractures and/or muscular pain or weakness may be manifestations of proximal renal tubulopathy and should prompt an evaluation of renal function in at-risk patients.

***Management per protocol:*** Renal function will be assessed using clinical standard of care practices. Creatinine clearance will be calculated using the CKD-Epi in all individuals prior to initiating TRUVADA, at 3, 12 and 30 months of follow-up and as clinically appropriate during therapy with TRUVADA.

Participants may require more frequent monitoring or additional tests (e.g., urinalysis for proteinuria) if other threats to renal safety are present (e.g., hypertension, diabetes).

Patients cannot be enrolled and TRUVADA® will not be used if creatinine clearance is less than 60 mL/min. If a decrease in creatinine clearance is observed after enrolment, the site investigator will evaluate potential causes and re-assess potential risks and benefits of continued use. A rise in serum creatinine is not a reason to withhold treatment if eCrCl remains ≥60 ml/min. TRUVADA® must be interrupted if the participant's renal function is confirmed to be abnormal, pending further investigation or specialist referral. TRUVADA® must be discontinued if the participant requires concurrent treatment with a nephrotoxic agent (e.g., high-dose non-steroidal anti-inflammatory drugs / NSAIDs).

#### 6.3.4 Bone Health

Per the US Public Health Service/Centers for Disease Control Preexposure prophylaxis for the Prevention of HIV Infection in the United States – 2014 Clinical Practice Guideline [36], three to four percent decreases in bone mineral density (BMD) have been observed in people being treated for HIV with combination antiretroviral therapy including tenofovir. [37] However, data published from PrEP studies that assessed BMD to date (iPrEx; CDC PrEP safety trial in MSM) found no increase in fragility (atraumatic) fractures over the one to two years of observation compared to placebo. In these studies, a small (about 1%) decline in BMD which was observed during the first few months of PrEP either stabilized or returned to normal. [38]

Based on this evidence the US Clinical Practice Guideline does not recommend DEXA scans or other assessments of bone health before the initiation of PrEP or for the monitoring of persons while taking PrEP. However, participants with a history of pathologic or fragility bone fractures or who have significant risk factors for osteoporosis will be asked to inform their doctors at the time of enrolment, so that they receive appropriate advice and management of their condition.

***Management per protocol:*** Participants will be assessed for a history of pathologic or fragility bone fractures and for significant risk factors for osteoporosis. Those who report this history will be referred for appropriate consultation and management.

#### 6.3.5 Co-administration with Other Products

TRUVADA® may not be co-administered with the following medications:

- concurrent or recent use of a nephrotoxic agent (e.g., high-dose non-steroidal anti-inflammatory drugs / NSAIDs)
- other drugs containing emtricitabine and/or tenofovir disoproxil fumarate (e.g., ATRIPLA, COMPLERA, EMTRIVA, STRIBILD, or VIREAD)
- other drugs containing lamivudine (due to similarities between emtricitabine and lamivudine)
- adefovir (HEPSERA)

Caution should be exercised when co-administering medications that are eliminated by active tubular secretion as they may increase concentrations of emtricitabine and/or tenofovir.

If a participant reports or requires the use of a prohibited medication, he or she must be discontinued from the study.

#### 6.3.6 Management of other events

In addition, the site investigator has the discretion to stop treatment at any time if she or he feels that further treatment would be harmful to the participant or would interfere with treatment that is deemed clinically necessary. The suspension or discontinuation of treatment will be considered for any persistent Grade II event, new Grade III event, or any Grade IV events measured as described in section 8, Recording and reporting Adverse Events (AEs).

When the study treatment is stopped, clinical staff will document the clinical and/or laboratory abnormalities that require follow up and schedule a follow-up visit for the repeat laboratory testing. All participants reporting an adverse event will be followed clinically until the occurrence resolves (returns to baseline grade, defined as grade at enrolment) or stabilizes. According to the judgement of the site investigator, if specialist care is required, a referral to appropriate specialist health care providers is accomplished.

TRUVADA® can be reintroduced within the next six months at the discretion of the clinician if the participant meets eligibility criteria and is willing to continue taking PrEP and after clinician's review of the reasons for previous discontinuation of PrEP and confirmation of eligibility for re-enrolment with the Protocol Chair.

## 6.4 Comprehensive Management to Reduce the Risk of Acquiring HIV-1

TRUVADA® will be used for PrEP only as part of a comprehensive prevention strategy that includes other prevention measures, such as safer sex practices, because TRUVADA® is not always effective in preventing the acquisition of HIV-1.

- Participants will be counselled about safer sex practices to include consistent and correct use of condoms, knowledge of their HIV-1 status and that of their partner(s), and regular testing for other sexually transmitted infections that can facilitate HIV-1 transmission (such as syphilis and gonorrhea).
- Participants will be informed about and supported in their efforts in reducing sexual risk behaviour.

HIV-1 resistance substitutions may emerge in individuals with undetected HIV-1 infection who are taking only TRUVADA, because TRUVADA® alone does not constitute a complete treatment regimen for HIV-1 treatment.

Many HIV-1 tests, such as rapid tests, detect anti-HIV antibodies and may not identify HIV-1 during the acute stage of infection. Prior to study enrolment and during the study, the site investigator will evaluate seronegative individuals for current or recent signs or symptoms consistent with acute viral infections (e.g., fever, fatigue, myalgia, skin rash, etc.) and will ask about potential exposure events (e.g., unprotected, or condom broke during sex with an HIV-1 infected partner) that may have occurred since the last visit.

If clinical symptoms consistent with acute viral infection are present **at screening** and recent (<30 days) exposures are suspected, the site investigator must delay starting PrEP for up to 30 days, and reconfirm HIV-1 status or use an approved test as an aid in the diagnosis of HIV-1 infection, including acute or primary HIV-1 infection.

Participants will receive HIV-1 testing in accordance with the follow-up schedule. If clinical symptoms consistent with acute HIV-1 infection are present after baseline following a potential exposure event which happened in the preceding three months, PrEP should be discontinued until negative infection status is confirmed using a test approved as an aid in the diagnosis of HIV-1, including acute or primary HIV-1 infection. Note this may require more than one episode of testing. During this time, the participant will be advised to eliminate all HIV risk behaviours until HIV status determined.

Participants will be counseled to strictly adhere to the recommended TRUVADA® dosing schedule.

## 6.5 Treatment interruption and discontinuation

In addition to the events above and the details under section 6.7 post-exposure prophylaxis (PEP), treatment will be stopped early for any of the following reasons:

- HIV infection (positive HIV test result or symptoms of seroconversion illness)
- Other unacceptable toxicity or adverse events
- Any change in the participant's sexual behaviour or circumstances that makes participant no longer eligible for PrEP (see eligibility criteria in Section 4.1)
- Participant need for a medication listed as prohibited for co-administration

- On the recommendation of the study's Data Safety and Monitoring Board (DSMB) (termination of the study by the Sponsor)

Adherence will be assessed at each visit upon discussion with the participant. Suboptimal adherence will be based on the site investigator's clinical judgement, in light of available evidence.[15] This includes participants who present for repeated requests for PEP in accordance with section 6.7 post-exposure prophylaxis (PEP).

Participants who otherwise declare to the site investigator non-adherence, but are willing and eligible to continue on PrEP, should undergo reinforced adherence education. Those who are due for a new PrEP prescription should be given a one month prescription, and should be scheduled for a visit one month later to reassess adherence before providing further prescriptions for PrEP.

If the site investigator believes that adherence is sufficiently suboptimal as to compromise PrEP efficacy and participant safety, the investigator will discontinue the participant from the study. Discussions about suboptimal adherence will be documented in the source documents. The third warning justifies the clinician's discontinuation of treatment.

The participant is free to interrupt or discontinue TRUVADA® but is required to inform his or her clinician about it. Participants will be discouraged from discontinuing TRUVADA® if they continue to meet criteria for high risk of HIV infection, but the prescription of TRUVADA® will stop if a participant is not fulfilling eligibility criteria any more. Even if participants are no longer taking TRUVADA, every attempt should be made to maintain them in the study for follow-up for up to six months after discontinuing the study medication.

If a participant does not wish to remain on the study follow-up, every effort will be made to establish an acceptable means to ascertain his or her HIV status at discontinuation and up to 6 months after stopping TRUVADA. However, should the participant withdraw from the study completely, she or he is free to do so and the appropriate case record should be made.

If a participant changes his or her mind about stopping TRUVADA® and the study follow-up, she or he can re-enter the study within the next 6 months after stopping the study medication if she or he satisfies all eligibility criteria for TRUVADA. However, any re-entry into the study will be stopped in the last 4 months of prescribing period of the PRELUDE study, or if the 300 person-year supply of study drug has already been allocated. Participants who stop the study follow-up before their Follow-up visit 3 (month 6) can be replaced by new participants in consultation with the sponsor (subject to the supply of the study medication).

## 6.6 Pregnancy and breastfeeding

TRUVADA® has been evaluated in a limited number of women during pregnancy and postpartum and there are no adequate and well-controlled trials in pregnant women. Because the studies in humans cannot rule out the possibility of harm, TRUVADA® should be used during pregnancy only if clearly needed. Careful consideration should be given to whether use of TRUVADA® should be continued during pregnancy.

The US Public Health Service Clinical Providers' Supplement "Preexposure Prophylaxis for the Prevention of HIV Infection in the United States" outlines recommendations for PrEP use for HIV-negative men and HIV-negative women planning pregnancy with an HIV-positive partner. In accordance with these guidelines, PrEP can be used as one of the options for HIV negative partners

in serodiscordant relationships who are planning to conceive. Any participants who are taking PrEP in conjunction with conception must take daily doses of TRUVADA® beginning one month before a conception attempt and continuing for one month after a conception attempt. Where relevant, fertility should be monitored at each follow-up.

In all cases when women conceive while on TRUVADA, the responsible clinician should discuss with the participant the risks and benefits of remaining on TRUVADA® for PrEP to reduce the risk of acquiring HIV during their pregnancy. The decision to continue should be made jointly by the participant and the clinician based on their discussion of risks and benefits. This discussion along with the decision whether or not to continue must be documented in the source documents.

The Participant Information and Consent Form (PICF) includes a provision for women of child-bearing potential to grant permission for the study team to access data on post-discontinuation HIV status, including postpartum status and infant status.

Studies in humans have shown that both tenofovir and emtricitabine are excreted in human milk. Additionally, the Centers for Disease Control and Prevention recommend that HIV-1 infected mothers not breast-feed their infants to avoid risking postnatal transmission of HIV-1. Because the risks of low level exposure to emtricitabine and tenofovir to infants are unknown, and because breastfeeding infants whose mothers are being treated with emtricitabine may be at risk for developing viral resistance to emtricitabine, women will not be allowed to start or continue TRUVADA® if they are breastfeeding.

## 6.7 Post-exposure prophylaxis (PEP)

If at any time after the baseline visit, a study participant seeks care within 72 hours after a high-risk sexual exposure and he or she has not yet started PrEP, or has missed the daily medication within 24 hours before and after the event, the following steps should be undertaken in conjunction with the national guidelines for post-exposure prophylaxis after non-occupational and occupational exposure to HIV:

- Assess HIV status with follow-up confirmation of the HIV negative test result as per standard of care specified by the national guidelines for post-exposure prophylaxis after non-occupational and occupational exposure to HIV. Copies of the results of these tests will be collected for the study.
- Prescribe a 28-day course of PEP as per standard of care and ask the participant to resume taking the study medication immediately upon the completion of PEP. **The labelled study medication cannot be used as part of a PEP regimen.**
- Schedule a follow-up assessment at four weeks from the exposure. At this standard of care PEP follow-up visit provide PrEP medication education and adherence enhancement; **do not prescribe more than one month of the study medication if the patient is out of supply.**
- Conduct the national PEP guidelines recommended three month follow-up visit one month earlier, at month two from PEP initiation. In addition to standard-of-care PEP assessments, assess adherence to PrEP during the preceding month (that is, the month following PrEP re-initiation), and assess risk behaviour and eligibility to continue with PrEP as per the study follow-up schedule.
- Data from these HIV tests will be collected for the PRELUDE Study.

**<u>IMPORTANT:</u>** ***PRELUDE Study investigational product must not be used for PEP.*** Conversions from PrEP to PEP must be notified to the sponsor via telephone, email or eCRF (electronic Case Report Form) completion within seven calendar days.

Second and subsequent reports to the study doctor that necessitate PEP (non-adherence during the time of a risk event) will be reviewed and documented by the site investigator. The decision as to whether or not to continue the participant on PrEP will be made by the investigator and the PRELUDE Study medical officer jointly on a case by case basis.

## 6.8 Treatment after HIV infection

Participants who acquire HIV infection will stop TRUVADA® and will be managed according to national treatment guidelines. [39,40] HIV-1 resistance substitutions may emerge in individuals with undetected HIV-1 infection who are taking only TRUVADA, because TRUVADA® alone does not constitute a complete HIV-1 treatment regimen. Resistance testing is strongly recommended per the Prescribing Information (e.g. M184V/I and/or K65R amino acid substitutions in the viral RT), and results will be collected if available.

These participants will also be offered to complete the PrEP study exit survey. Seroconversion will also be documented on a seroconversion eCRF and in the source documentation, and photocopies of HIV confirmatory tests will be collected for all suspected seroconversions. The site must report all HIV seroconversions to the sponsor immediately by telephone or email within 24 calendar hours of site awareness.

## 7.0 Study assessments and follow-up procedures

A study process flowchart is presented in Appendix V, and an overview of study visits is presented in table 2. The description of the assessments and procedures that will be carried out at each visit follows. These will include:

### 7.1 Screening and enrolment

#### 7.1.1 Pre-Screening

Once a patient presents for possible PrEP, investigators at participating clinics will provide potential candidates with information on PrEP as well as PEP. Interested patients will be provided with the pre-screening Patient Information and Consent Form (PICF, Appendix VI), which allows for the collection of non-identifiable pre-screening information in conjunction with study objective 2.

The site investigator or equivalent trained designee will be responsible for ensuring that the potential participant understands all aspects of *pre-screening* prior to obtaining consent. This consent will not be subject to any coercion, or to any inducement or influence that could impair its voluntary character. The participant will be given time to consider pre-screening and to ask any questions. The participant will be given a copy of the signed and dated pre-screening PICF. Copies of the pre-screening consent forms will be kept on file at the clinic, separate from the non-identifiable pre-screening data.

Upon completion of the pre-screening consent, the site investigator or designee will record on a paper case report form (CRF) (Pre-screening Questionnaire) the answers to the risk criteria in Appendix III Boxes A and B, algorithm for identifying individuals at risk for acquiring HIV infection. The potential participant's age, gender and sexual orientation, and the date of the visit, will also be recorded. The CRF will contain a pre-printed unique identification number which can be used to link data if the potential participant progresses to the PRELUDE study. If any changes are made after the participant discussion with the doctor, the changes should be marked, initialled and dated by the participant followed by the doctor. This will ensure that the most accurate data is reported on the pre-screening form.

The potential participant will be also be given a tear-off from the paper-CRF, which will include a unique identification number and a link to a website to complete an attitude, behavioural and lifestyle survey. The survey works best and should be completed on a desktop or laptop computer. The attitude, behavioural and lifestyle survey, which will be de-identified and only linked by the unique identification number, will include questions on reasons for interest, and reasons for declining participation, if applicable.

If the patient's risk assessment qualifies him or her for the study, the patient will progress to the informed consent process for PRELUDE.

#### 7.1.2 Informed consent process

At this stage, participants will be given a copy of the PRELUDE PICF (appendix VII) and a verbal explanation of the study purpose and procedures. Patients will have an opportunity to discuss the

information about the study and eligibility criteria screening with a member of the study participating clinical team and to ask for any clarification.

After receiving detailed information about the study goals and procedures, each prospective participant will provide individual written consent to:

- Participate in the study.
- Provide routinely collected clinical data including HIV and STI testing. (Participants will visit clinics up to 6 times per year as per study protocol).
- Complete a computer-based survey regarding risk behaviour and attitudes about PrEP, PEP and HIV risk reduction in general.
- Allow the study investigators from the Kirby Institute to access data from the HIV Registry for linkage purposes. This will allow information to be obtained about HIV seroconversion in the case of participants' loss to follow-up before seroconversion.
- Grant permission to link the non-identifiable pre-screening data on the CRF and from the attitude, behavioural and lifestyle survey, back to the PRELUDE study data.
- Be willing and able to have blood collected at the scheduled follow-up visits.

The site investigator or equivalent trained designee will be responsible for ensuring that the participant understands all aspects of the study prior to obtaining consent. The consent of a person to participate will not be subject to any coercion, or to any inducement or influence that could impair its voluntary character. The participant will be given time to consider the research project and to ask any questions prior to entry into the study.

The participant will be given a copy of the signed and dated PRELUDE PICF. Copies of the consent forms will be kept on file at the clinic. No locally authorized representative option will be available for this study.

The participant will be enrolled into the study after the informed consent process has been completed and the participant has met all eligibility criteria. The participant's unique study number from pre-screening will be assigned upon completion of the enrolment eCRF, and this will be documented in the participant's medical record and on all study documents.

Procedures that are to be performed as part of the standard clinical practice and which would be done whether or not study entry was contemplated, such as for diagnosis or treatment of a disease or medical condition, may be used for determining study eligibility without first obtaining consent. Otherwise, informed consent must be obtained prior to initiation of any clinical screening procedures that are performed solely for the purpose of determining eligibility for research.

### 7.1.3 Screening visit

Prospective participants will undergo the following investigations as recommended by the STI testing guidelines:

- HIV (note HIV negative status must be obtained within seven days of PrEP initiation).
- Rectal and/or vaginal chlamydia (nucleic acid amplification test)
- Rectal and/or vaginal gonorrhoea (nucleic acid amplification test)
- Syphilis testing per standard of care

Hepatitis B status and exposure risk over preceding three months will be checked, and immunisation against hepatitis B promoted and delivered.

In addition, the site investigator will solicit a history of nonspecific signs or symptoms of viral infection during the preceding 30 days.

Results from any screening tests that were completed as standard of care before consent was obtained need not be repeated as long as the HIV test has been conducted within seven days of starting PrEP and there were no high risk events of exposure to HIV during this time, and the other tests have been conducted within 14 days of the screening visit. This excludes prior positive Hepatitis B serology and syphilis IA results, which may have been conducted prior to 14 days of screening.

All other screening visit procedures must be conducted within two weeks of the study enrolment/baseline visit. If there are no reasons for exclusion based on the available information, and the client wishes to proceed, an appointment will be made for an enrolment/baseline visit, which may happen on the same day as all test results are available and no longer than seven days after the last HIV negative test results were obtained.

Potential participants who complete the screening process but either do not qualify for the study or decide not to enroll will be asked to complete the online survey of behaviour and lifestyle which will also assess reasons for not qualifying for or deciding not to enroll in the study.

### 7.1.4 Enrolment/Baseline visit

The enrolment/baseline visit will be considered finalized when all of the following procedures are completed. Some of these procedures may be completed in conjunction with the Screening Visit.

<u>Administrative Procedures and Eligibility Evaluations (performed by clinical staff)</u>

- Informed consent as outlined above
- Locator information
- HIV/STI behavioural counselling (pre- and post-test), including risk-reduction counselling
- PrEP discussion
- HIV prevention resources (including condoms) offered
- Completion of eligibility criteria source documentation

*Note: In general, sites must refer to the NSW Interim guidance for PrEP administration. More details will be provided in site specific SOPs.*

<u>Clinical Evaluations/ Procedures (performed by a licensed clinician)</u>

- Complete medical history including but not limited to sociodemographic Information, sexual risk history and any known risks related to sexual partners, history of ARV use; concomitant medications; medication adherence needs assessment
- Symptom-driven physical exam
- Standard-of-care STI testing and, if indicated, STI treatment

<u>Laboratory Evaluations/ Procedures (samples collected by licensed provider)</u>

- HIV testing (negative HIV test result documented within seven days of initiating PrEP).
- For patients exhibiting clinical signs/symptoms of acute seroconversion illness, conduct the standard-of-care confirmatory algorithm.
- Renal function tests per standard of care, Creatinine and estimated glomerular filtration rate (eGFR)
- Standard of care liver function tests (protein, albumin, bilirubin, ALP, ALT, AST, GGT)

- Hepatitis B testing per standard of care, as guided by the current National Hepatitis B Testing Policy.
- STI testing: rectal ± vaginal gonorrhoea (GC) and chlamydia (CT), and urine for men – NAAT
- Pregnancy test for all women of child-bearing potential
- Syphilis testing per standard of care

Note: Participants who do not have evidence of immunity to HBV will be offered HBV vaccination.

Behavioural and lifestyle data collection

- Self-administered online attitude, behavioural and lifestyle survey, completed ideally within two and no more than seven calendar days of the clinic visit.

PrEP initiation / recommencement

Day 0 represents the first day participants receive the first prescription for PrEP, and may occur on the same day as the enrolment/baseline visit. For participants who have discontinued and are subsequently re-initiating PrEP under the protocol, procedures at a recommencement visit will be the same as procedures at enrolment/baseline.

- Assess participant's initial readiness for PrEP adherence
- Assess participant's potential exposure to a high risk even since the pre-screening visit. **If a participant reports a high-risk sexual HIV exposure before initiation of PrEP, delay PrEP initiation and begin a course of PEP in accordance with section 6.7 Post-exposure prophylaxis (PEP).**
- Give TRUVADA® prescription for 30 days. (One month. Adjust accordingly for those recommencing PrEP who may have left-over medication.)
- Provide supportive adherence counselling and set PrEP adherence goals
- Ensure HIV test was conducted within seven days of medication start. If the HIV test was conducted more than seven days before the participant is due to start taking PrEP, negative HIV status must be re-confirmed within the seven-day window.

The study staff will explain how to complete the attitude, behavioural and lifestyle survey and make sure that participants fully understand the process of behavioural data collection. Participants will then be asked to complete the survey online, in private. If the participant does not complete the behavioural survey within the preferred timeframe and neither after he or she has received two online reminders, the Kirby Institute researchers may ask the study staff to contact the participant to contact you to provide another reminder. If the participant does not respond to the reminders, the study team will consider the participant as not willing to take the computer-based survey. The participant will therefore no longer meet the study criteria and will not be able to receive PrEP through the study. These participants can however, obtain access PrEP outside the study if required. Therefore, to qualify for the study, the participants must be willing to and complete the computer–based survey online, among meeting other criteria in the study.

The behavioural and lifestyle information provided by the individual and available in the clinic record will inform the discussion about HIV/STI risk reduction and adherence to the daily TRUVADA® schedule. However, the information provided in the online attitude, behavioural and lifestyle survey will be kept separate from the clinical data and will not inform these discussions.

The participant will be provided with a visit follow-up schedule. The research nurses or designees will coordinate the clinical follow-up process.

## 7.2 Follow-up visits of participants on PrEP

All follow-up visits are calculated from Day 0 (baseline visit for either the first PrEP prescription, or a subsequent re-initiation of a PrEP prescription). Table 2 lists the procedures to be conducted at each visit and allowable visit windows, with further details provided below. Any visits conducted outside of the visit window will be considered protocol deviations, as will any missed required visit elements.

A PrEP treatment discontinuation visit will be conducted for all participants who cease study medication prior to the 30 month visit. Such participants will continue to be followed up with attitude, lifestyle and behavioural survey data collection according to the protocol study plan. Participants may revoke consent for follow-up without jeopardizing their relationship with either their doctor or the sponsor. If a participant revokes consent then, if possible, all assessments scheduled for the final Follow-up visit should be completed.

### 7.2.1 Follow-up visit 1 (1 month after enrolment/recommencement)

*Conduct a visit consisting of Follow-up 1 procedures one month after initiating PrEP, or one month after a participant re-initiates PrEP.*

Administrative and Behavioural Evaluations/ Procedures

- Review and update locator information
- Review and confirm eligibility for PrEP
- HIV/STI behavioural counselling, including risk-reduction counselling
- PrEP discussion
- Offer condoms and other prevention supplies
- Assess participant adherence to PrEP in the period since enrolment

Clinical Evaluations/ Procedures

- Assess interim medical history including concomitant medications and TRUVADA® specific side-effects

Laboratory Evaluations/ Procedures

- HIV testing
- For patients exhibiting clinical signs/symptoms of acute seroconversion illness, conduct the standard-of-care confirmatory algorithm.
- Pregnancy test for all women of child-bearing potential
- Collection of plasma and PBMCs for TRUVADA® drug levels (if participating)
- Collection of serum for storage (if participating)

Behavioural and lifestyle data collection

- Self-administered online attitude, behavioural and lifestyle survey, completed ideally within two and no more than seven calendar days of the clinic visit (invitation triggered by date of the start of the follow-up).

PrEP prescription

- Assess participant adherence to PrEP in the period since enrolment

- If the participant is reporting a known or suspected exposure to HIV within 72 hours and has missed the daily medication within 24 hours before and after the event, discontinue PrEP and recommend to initiate PEP according to national PEP guidelines. [41]
- If eligibility criteria are met, and participant is willing to continue taking PrEP, give TRUVADA® prescription for 30 days
- Provide supportive adherence counselling
- If it is known that the participant is entering the last period on study medication, inform participant that this is the last prescription of TRUVADA® given by the study, and emphasize the need for the final follow-up visit to develop a plan for HIV risk reduction following the exit from the study.

### 7.2.2 Follow-up visit 2 (3 month since enrollment)

*Conduct a visit consisting of Follow-up 2 procedures three months after initiating PrEP, or two months after the Follow-up visit 1.*

Administrative and Behavioural Evaluations/ Procedures

- Review and update locator information
- Review and confirm eligibility for PrEP
- HIV/STI behavioural counselling, including risk-reduction counselling
- PrEP discussion
- Offer risk reduction information (including condoms)
- Assess participant adherence to PrEP in the period since enrolment

Clinical Evaluations/ Procedures

- Assess interim medical history including concomitant medications and TRUVADA® specific side-effects
- Collect samples for standard of care laboratory evaluations

Laboratory Evaluations/ Procedures

- HIV testing
- For patients exhibiting clinical signs/symptoms of acute seroconversion illness, conduct the standard-of-care confirmatory algorithm.
- Renal function tests per standard of care, Creatinine and estimated glomerular filtration rate (eGFR)
- Liver function tests (protein, albumin, bilirubin, ALP, ALT, AST, GGT)
- STI testing: syphilis testing per standard of care, rectal ± vaginal gonorrhoea (GC) and chlamydia (CT) –NAAT and/or culture
- Pregnancy test for all women of child-bearing potential

Behavioural and lifestyle data collection

- Self-administered online attitude, behavioural and lifestyle survey completed ideally within two and no more than seven calendar days of the clinic visit (invitation triggered by the completion of clinical visit record)

PrEP prescription

- Assess participant adherence to PrEP in the period since the last follow-up visit

- If the participant is reporting a known or suspected exposure to HIV within 72 hours and has missed the daily medication within 24 hours before and after the event, discontinue PrEP and recommend to initiate PEP according to national PEP guidelines.[41]
- If eligibility criteria are met, and participant is willing to continue taking PrEP, give TRUVADA® prescription for 30 days with two refill prescriptions for 30 more days each
- Provide supportive adherence counselling and set PrEP adherence goals for the following period till next follow-up visit
- If it is known that the participant is entering the last period on study medication, inform participant that this is the last prescription of TRUVADA® given by the study, and emphasize the need for the final follow-up visit to develop a plan for HIV risk reduction following the exit from the study.

### 7.2.3 Follow-up visits 3 and 4 (6 and 9 months since enrollment)

*Conduct a visit consisting of Follow-up 3 procedures six months after initiating PrEP (or three months after the Follow-up visit 2) and Follow-up 4 procedures - nine months after initiating PrEP (or three months after the Follow-up visit 3.*

<u>Administrative and Behavioural Evaluations/ Procedures</u>

- Review and update locator information
- Review and confirm eligibility for PrEP
- HIV/STI behavioural counselling, including risk-reduction counselling
- PrEP discussion
- Offer risk reduction information (including condoms)
- Assess participant adherence to PrEP in the period since last follow-up visit

<u>Clinical Evaluations/ Procedures</u>

- Assess interim medical history including concomitant medications and TRUVADA® specific side-effects
- Collect samples for standard of care laboratory evaluations

<u>Laboratory Evaluations/ Procedures</u>

- HIV testing
- For patients exhibiting clinical signs/symptoms of acute seroconversion illness, conduct the standard-of-care confirmatory algorithm
- If the participant tested positive for hepatitis B surface antigen (HBsAg) at screening, test for HBV DNA by the use of a quantitative assay month 6 and every six months
- Liver function tests (protein, albumin, bilirubin, ALP, ALT, AST, GGT)
- STI testing: syphilis testing per standard of care, rectal gonorrhea (GC) and chlamydia (CT) – NAAT and/or culture
- Pregnancy test for all women of child-bearing potential
- Collection of plasma and PBMCs for TRUVADA® drug levels, six month follow-up only (if participating)
- Collection of serum for storage, six month follow-up only (if participating)

<u>Behavioural and lifestyle data collection</u>

- Self-administered online attitude, behavioural and lifestyle survey, completed ideally within two and no more than seven calendar days of the clinic visit (invitation triggered by the completion of clinical visit record)

PrEP prescription

- Assess participant adherence to PrEP in the period since the last follow-up visit
- If the participant is reporting a known or suspected exposure to HIV within 72 hours and has missed the daily medication within 24 hours before and after the event, discontinue PrEP and recommend to initiate PEP according to national PEP guidelines. [41]
- If eligibility criteria are met, and participant is willing to continue taking PrEP, give TRUVADA® prescription for 30 days with two refill prescriptions for 30 more days each
- Provide supportive adherence counselling and set PrEP adherence goals for the following period till next follow-up visit
- If it is known that the participant is entering the last period on study medication, inform participant that this is the last prescription of TRUVADA® given by the study, and emphasize the need for the final follow-up visit to develop a plan for HIV risk reduction following the exit from the study.

### 7.2.4 Follow-up visits 5 to12 (12-30 months since enrollment)

*Conduct a visit consisting of Follow-up 5 procedures at 12 months after initiating PrEP and every three months thereafter (up until month 30) OR at the exit from the study*

Administrative and Behavioural Evaluations/ Procedures

- Provide HIV/STI behavioural counselling, including risk-reduction counselling and develop a plan for patients risk reduction after discontinuing TRUVADA® provided by the study
- Discuss patients experience on PrEP
- Offer risk reduction information (including condoms)
- Assess participant adherence to PrEP in the period since last follow-up visit

Clinical Evaluations/ Procedures

- Assess interim medical history including concomitant medications and TRUVADA® specific side-effects
- Collect samples for standard of care laboratory evaluations

Laboratory Evaluations/ Procedures

- HIV testing
- For patients exhibiting clinical signs/symptoms of acute seroconversion illness, conduct the standard-of-care confirmatory algorithm.
- If the participant tested positive for hepatitis B surface antigen (HBsAg) at screening, test for HBV DNA by the use of a quantitative assay every six months after PrEP initiation.
- Renal function tests per standard of care, Creatinine and estimated glomerular filtration rate (eGFR)
- Liver function tests (protein, albumin, bilirubin, ALP, ALT, AST, GGT)
- STI testing: syphilis testing per standard of care, rectal gonorrhea (GC) and chlamydia (CT) – NAAT and/or culture
- Pregnancy test for all women of child-bearing potential
- Collection of plasma and PBMCs for TRUVADA® drug levels (if participating)
- Collection of serum for storage (if participating)

Behavioural and lifestyle data collection

- Self-administered online attitude, behavioural and lifestyle survey, completed ideally within two and no more than seven calendar days of the clinic visit (invitation triggered by the completion of clinical visit record). This will include data on attitudes to PrEP, willingness to take it in the future, satisfaction with PrEP and services provided within the study

PrEP prescription

- Assess participant adherence to PrEP in the period since the last follow-up visit
- If the participant is reporting a known or suspected exposure to HIV within 72 hours and has missed the daily medication within 24 hours before and after the event, discontinue PrEP and recommend to initiate PEP according to national PEP guidelines. [41]
- If eligibility criteria are met, and participant is willing to continue taking PrEP, give TRUVADA® prescription for 30 days with two refill prescriptions for 30 more days each
- Provide supportive adherence counselling and set PrEP adherence goals for the following period till next follow-up visit.
- If it is known that the participant is entering the last period on study medication, inform participant that this is the last prescription of TRUVADA® given by the study, and emphasize the need for the final follow-up visit to develop a plan for HIV risk reduction following the exit from the study.

PrEP discontinuation

- Assess participant adherence to PrEP since last follow-up visit
- If the participant is reporting a known or suspected exposure to HIV within 72 hours and has missed the daily medication within 24 hours before and after the event, recommend to initiate PEP according to national PEP guidelines. [41]
- Discuss short-term (next 30 days) and long term HIV risk reduction for the period following discontinuation of PrEP

### 7.2.5 Interim Visits

Interim visits may occur at any time during the study. If a participant presents for a visit outside of a visit window (e.g. because of a missed visit, to discuss problems with adherence or an adverse event), the interim visit will be documented on an interim visit case report form (CRF) and in the source documentation.

## 7.3 Follow-up of patients after discontinuing PrEP

Participants may choose to discontinue from taking PrEP but remain on the study. In that case they can still complete the discontinuation and post-discontinuation follow-up visits. All study participants who discontinue PrEP, irrespective of whether they reached the final medication Follow-up visit 11 (30 months on PrEP), will be scheduled to have a study visit three months after their last follow-up visit on PrEP. The assessment will include:

### 7.3.1 Follow-up visit 12 (33 months in the study)

*Conduct a visit consisting of Follow-up 12 as a standard of care assessment procedures at335 months after initiating PrEP or 3 months after discontinuing PrEP.*

Laboratory Evaluations/ Procedures

- HIV testing including serology tests as per standard of care with Western Blot for confirmation.
- (if conducted by the clinic) proviral DNA for suspected seroconverters
- STI testing: syphilis (early infection – EIA, RPR, TPPA, FTA-ABS), rectal ± vaginal gonorrhea (GC) and chlamydia (CT) – NAAT and/or culture

Behavioural and lifestyle data collection

- Self-administered online attitude, behavioural and lifestyle survey (invitation triggered by the completion of clinical visit record), which will include attitudes to PrEP, willingness to take it in the future

PrEP discontinuation

- Assess participant adherence to PrEP since last follow-up visit
- If the participant is reporting a known or suspected exposure to HIV within 72 hours and has missed the daily medication within 24 hours before and after the event, recommend to initiate PEP according to national PEP guidelines. [41]
- Discuss short-term (next 30 days) and long term HIV risk reduction for the period following discontinuation of PrEP

### 7.3.2 Follow-up 13 (36 months in the study)

*This is not a clinical visit, but a follow-up survey. Conduct data collection consisting of Follow-up 13 procedures at 36 months after initiating PrEP or 6 months after discontinuing PrEP.*

Behavioural and lifestyle data collection

- Self-administered online behavioural, lifestyle and attitudes survey (invitation scheduled at the end of the completion of clinical Follow-up visit 12 )

**Table 2: Follow Up Visit Procedures and Visit Windows**

| **Visit/data collection point** | **Visit 2 Month 1** | **Visit 3 Month 3** | **Visit 4 Month 6** | **Visit 5- Month 9** | **Visit 6-11 Month 12-27** | **Visit 12 Month 30 or Treatment D/c visit** | **Visit 13 Month 33 or 3 months post-D/C** | **Data collection only 14 Mo 36 or 6 mos post-D/C** |
|---|---|---|---|---|---|---|---|---|
| Visit window | (+/- 7 days) | (+/- 14 days) | (+/- 14 days) | (+/- 14 days) | (+/- 14 days) | (+/- 14 days) | (+/- 14 days) | (+/- 14 days) |
| Administrative procedures | X | X | X | X | X | X[a] | X | |
| Interim history, concomitant medication, adverse events | X | X | X | X | X | X | | |

| | | | | | | | | |
|---|---|---|---|---|---|---|---|---|
| HIV testing | X | X | X | X | X | X | X[b] | X[b] |
| STI testing | | X | X | X | X | X | X | |
| Renal function, calculated creatinine clearance | | X* | | X* | X* | X* | | |
| Hematology, LFTs | | X | X[c] | X[c] | X | X[c] | | |
| Pregnancy test if applicable | X[d] | X[d] | X[d] | X[d] | X[d] | X[d] | X[d] | |
| Adherence assessment | X | X | X | X | X | X | | |
| Collection of plasma, PBMCs and serum | X[f] | | X[f] | X[f] | X[f] | | | |
| Study medication prescribed | X | X | X | X | X | | | |
| Attitude, behavioral and lifestyle survey | X[e] | X[e] | X[e] | X[e] | X[e] | X[e] | X | X |

D/C = discontinuation

X[a] including study exit procedures and exit survey

X[b] HIV status post-PrEP discontinuation may be obtained via data linkage

X[c] LFTs only

X[d] for all women of child-bearing potential

X[e] completed ideally within three but no more than seven days of the clinic visit.

X[f] Only applicable to participants who provided consent for blood sample collection. Blood collected at month 1, month 6 and month 12.

X* Renal function test should be assessed every 6 months on alternative visits, i.e visit 7, 9 and 11.

## 7.4 HIV Counseling and Testing

HIV test-related counselling is available to all potential study participants who consent to undergo HIV screening to determine their eligibility for this study, and to all enrolled participants throughout the duration of the study. Testing will be performed in accordance with the study follow-up visit schedule. Counselling will additionally emphasize the current evidence about the efficacy of the daily TRUVADA® as PrEP, the association between medication adherence and its effectiveness and any new evidence about PrEP in preventing HIV infection.

Condoms will be provided to participants throughout the duration of their participation.

### 7.4.1 Care for Participants Identified as HIV-Positive

Participants identified as HIV positive prior to enrolment will be managed or referred for HIV management according to the current standard of care.

Should a study participant test positive for HIV after enrolment, follow-up procedures will be performed as per Section 6.5. A M1841/V and K65R mutation test results obtained per standard of care will be collected for the PRELUDE Study with participant consent.

# 8.0 Recording and reporting Adverse Events (AEs)

The site investigator has a responsibility to ensure that the monitoring of safety and reporting of adverse outcomes complies with the study protocol. The site investigator is responsible for the initial evaluation and reporting of safety information for study participants. Each site is required to have written procedures for toxicity management and AE reporting.

Adverse events may occur at any time after study enrollment throughout the follow-up period. These events may also occur in screened participants after informed consent is obtained as a result of protocol-specified interventions. All such events will be recorded at each study visit on an adverse event case report form.

## 8.1 Adverse Event definitions

The following adverse event definitions apply to this study.

**Adverse event (AE)**: An adverse event is any untoward medical occurrence in a patient or clinical investigation subject administered a pharmaceutical product ***and which does not necessarily have a causal relationship with this treatment***. An adverse event can therefore be any unfavourable and unintended sign, symptom, or disease temporally associated with the use of a medicinal (investigational) product, whether or not related to the medicinal (investigational) product.

For this study, any overdose of TRUVADA, whether or not accompanied by any signs or symptoms, will also be considered an adverse event.

Adverse Events do not include:

- Medical or surgical procedures. The condition that leads to the procedure is the adverse event.
- Hospitalisations where no untoward or unintended response has occurred, eg, elective cosmetic surgery, social admissions.
- Pre-existing conditions or diseases that exist at or prior to baseline/enrollment (e.g. seasonal allergies, asthma or recurrent headaches), unless they worsen in frequency or severity after administration of study drug.

**Serious Adverse Event (SAE):** A serious adverse event is any untoward medical occurrence that at any dose:

- results in death;
- is life-threatening*;
- requires in-patient hospitalisation or prolongation of existing hospitalisation$^{+}$;
- results in persistent or significant disability/incapacity; OR
- is a congenital anomaly/birth defect.

*The term “life-threatening” in the definition of “serious” refers to an event/reaction in which the participant was at risk of death at the time of event/reaction. It does not refer to an event/reaction which hypothetically might have caused death if it were more severe, for example, a silent myocardial infarction.

+Hospitalization is defined as an inpatient admission, regardless of length of stay, even if the hospitalisation is a precautionary measure for continued observation. Hospitalisations for a pre-existing condition (including elective procedures that have not worsened) do not constitute an SAE.

Medical and scientific judgment should be exercised in deciding whether other situations should be considered serious e.g. important medical events that may not be immediately life-threatening or result in death or hospitalisation, but may jeopardise the participant or may require intervention to prevent one of the outcomes listed in the definition above. Examples of such events include a medical even that leads to the discontinuation of TRUVADA, intensive treatment in an emergency room or at home for allergic bronchospasm, blood dyscrasias or convulsions that do not result in hospitalization, or development of drug dependency or drug abuse.

Note: pregnancy, overdose and cancer are classified as Serious for data collection purposes.

**Suspected Unexpected Serious Adverse Reaction (SUSAR):** A serious adverse event which is both suspected as being related to the drug (i.e. has a reasonable suspected causal relationship) and is unexpected, e.g. the nature and severity is not consistent with known information (e.g. the Product Information) about the drug in question.

## 8.2 Adverse Event assessments

Site investigators, the project team, the medical officer and the Data Safety Monitoring Board (DSMB) will use the following AE assessment scales for this study.

***Causality assessment:***

Causality is the likelihood that a particular treatment is the cause of an observed adverse event. The site investigator shall assign causality to all serious adverse events only.

The following binary causality assessment will be used for this study:

- **Related** – There is a reasonable possibility that the AE may be related to the study agent(s).
- **Not Related** – There is not a reasonable possibility that the AE is related to the study agent(s).

A reasonable possibility means there is evidence to suggest a causal relationship between the investigational product and the adverse event. Facts (evidence) or arguments that may support "a reasonable possibility" may include:

- a temporal relationship (i.e., AE is reasonably related in time to the use of the study drug)
- a pharmacologically or biologically plausible event

Presence of confounding factors, such as concomitant medication, concurrent illness, or relevant medical history, should also be considered.

When an SAE is assessed as "not related" to study agent(s), an alternative etiology, diagnosis, or explanation for the SAE should be provided.

For the purposes of reporting to the lead HREC, "not related" per the protocol will be reported to the HREC as "unrelated." "Related" per the protocol may be reported to the HREC per site investigator assessment as either "possibly related," "probably related" or "definitely related."

***Expectedness assessment:***

If the event is considered related, the site investigator must assess the event's expectedness. An unexpected adverse drug reaction (UADR) is defined as an adverse reaction, the nature or severity of which is not consistent with the applicable scientific information (e.g. Investigator's Brochure or Product Information (PI)/package insert/summary of product characteristics. It can also be an unexpected adverse reaction that is more frequent or more severe than previously reported.

Appendix IV lists expected toxicities associated with the study drug.

***Severity Assessment:***

In the classification of adverse events, the term "severe" is not the same as "serious." Severity is an indication of the intensity of a specific event (as in mild, moderate, or severe chest pain). The term "serious" relates to a participant/event outcome or action criteria per the SAE definition.

All AEs will be graded for severity using the DAIDS AE Grading Table, Version 1.0, December 2004 (Clarification dated August 2009) and the DAIDS Addendum 2 - Male Genital Grading Table, which are available at http://rsc.tech-res.com/safetyandpharmacovigilance/. In cases where a genital AE is covered in both tables, the Rectal Grading Table for Use in Topical Microbicide Studies will be the grading scale utilized.

For any AE where the outcome is death, the severity of the AE is classified as Grade 5.

Grade 1 creatinine toxicity will be defined as: greater than 50 is mild; 30-49 is moderate; less than 30 is severe.

## 8.3 Adverse Event Reporting Requirements

### 8.3.1 Reporting Requirements for sites

***Adverse Events***

Adverse events and/or laboratory abnormalities identified in the protocol as critical to safety evaluations should be reported to the sponsor according to reporting requirements and within the time periods specified in Table 2 Follow Up Visit Procedures and Visit Windows. Because most reported events related to TRUVADA® have been mild, information regarding all AEs regardless of seriousness, relatedness and expectedness will be recorded in the participant's source documents and entered into the eCRF.

All critical laboratory values will be reported as applicable per the site's SOPs.

With appropriate permission of the participant, and whenever possible, records from non-study medical providers related to untoward medical occurrences will be requested and required data elements will be recorded on study CRFs and/or in the participant's medical chart.

All AEs regardless of severity will be followed clinically, until the AE resolves or stabilizes as per the appropriate toxicity algorithm.

***Serious Adverse Events (Including Suspected Unexpected Serious Adverse Reactions or SUSARs)***

The site investigator should determine at each visit whether or not a Serious Adverse Event has occurred since the previous visit.

All serious adverse events (SAEs) must be reported to the sponsor by telephone or email immediately, and no later than within 1 business day of site awareness of the event. During public holidays that last more than one business day, all SAEs must be reported to the sponsor with 48 hours of site awareness of the event.

The appropriate Serious Event eCRF shall be used. This reporting requirement pertains to all SAEs that occur from baseline/enrolment through 3 months after the last protocol treatment administration, regardless of expectedness or relatedness per SUSAR requirements. The KI Project Team in collaboration with the KI Medical Officer will review and identify all serious events which fit the criteria of a SUSAR and requiring additional expedited reporting to relevant parties.

The site Investigator must immediately and no later than 72 hours after occurrence of the event report all SAEs and SUSARs occurring at the site directly to the St. Vincent's Hospital HREC via email using its Serious Adverse Event and Suspected Unexpected Serious Adverse Event Form. In addition, one hard copy of all documents, with original signatures, must be submitted to the Research Office.

The relevant site investigator will also provide a copy of these reports with the HREC acknowledgement of the report to his or her relevant Research Governance Officer.

Immediate reports should be followed promptly by detailed, written follow-up reports when all information is not included in the initial report. For deaths, the site investigator will supply the sponsor and the HREC with any additional requested information (e.g. death certificate, autopsy reports and medical reports).

For participants who discontinue and then re-enroll in the study, any AEs that happened during the period not on follow-up should be reported within the required time periods from the moment they were detected by the clinical study personnel.

***Other events requiring expedited reporting:***

The site must also report:

- All HIV seroconversions to the sponsor immediately by telephone or email, and no later than within 24 hours of site awareness.
- All participant conversions from PrEP to PEP to the sponsor by telephone, email or eCRF submission, within seven calendar days.

The site investigator must also report to the lead HREC in a prompt manner, any:

- Information which materially impacts the continued ethical acceptability of the trial.
- Information that requires, or indicates the need for, a change to the study protocol, including changed safety monitoring in the view of the sponsor.

### 8.3.2 Reporting requirements for the Sponsor

For this study, the Kirby Institute has assumed the duties of the study sponsor and is thus responsible for the reporting of SUSARs to investigators, the HREC, the TGA, and Gilead Sciences, Inc. as required.

Kirby Institute medically-qualified staff and/or a medically-qualified delegate will review all SAE reports received. The causality assessment given by the site investigator cannot be overruled; in the case of disagreement, both opinions will be recorded and provided in any subsequent reports.

The Kirby Institute Project Team in collaboration with the study's Medical Officer will review and identify all serious events which fit the criteria of a SUSAR. Fatal and life-threatening SUSARs will be reported to the TGA within 7 calendar days of the sponsor becoming aware of the event. Other SUSARs will be reported to the TGA within 15 calendar days of awareness.

The study Principal Investigator will also keep all site investigators informed of any safety issues that arise during the course of the trial.

***Gilead Reporting:***

The sponsor will alert Gilead Drug Safety and Public Health (DSPH) of any potential safety issues arising in the study or any protocol amendments or changes to the informed consent arising from a safety concern associated with the Drug within 15 days of awareness.

All SAEs including any deaths, product complaints with an associated SAE or reports of pregnancy, medication error or overdose regardless of an associated SAE occurring during the study will be reported by the sponsor to Gilead DSPH within 15 calendar days of site awareness and in accordance with applicable laws, rules, regulations and guidance.

Upon Gilead's request the site investigator will provide any additional information related to any SAEs including any deaths, product complaints with an associated SAE or reports of pregnancy, medication error or overdose regardless of an associated SAE occurring during the study.

# 9.0 Packaging, labeling, storage and accountability of clinical study supplies

The blue, capsule-shaped, film-coated, tablets contain 200 mg of emtricitabine and 300 mg of tenofovir disoproxil fumarate (which is equivalent to 245 mg of tenofovir disoproxil), are debossed with "GILEAD" on one side and with "701" on the other side, and are available in unit of use bottles of 30 tablets (NDC 61958-0701-1).

TRUVADA® will be provided in bulk by Gilead Sciences, and redistributed for study use by Pharmaceutical Packaging Professionals (PPP) Pty Ltd, Suite 5, Lot 1, 40–46 West Thebarton Rd, Thebarton South Australia 5031.

TRUVADA® (FTC/TDF) study tablets must be stored below 25°C (as per TRUVADA® Product Information v.13 23 October 2013). FTC/TDF tablets must be stored in the original container. Each container is packaged with a child-resistant screw cap and contains a silica gel to protect the product from humidity.

Responsibility for investigational product accountability at the study site rests with the site Investigator/ institution. A designated person at the study site must receive clinical trial supplies. That person must check that the supplies are in good condition and are complete as per the shipping records. Drugs must be stored in a secure location with limited access. Clinical trial supplies must only be dispensed according to the protocol and records must be kept detailing supplies received, dispensed to the participant, returned from the participant and returned to the sponsor or destroyed at site, as applicable.

Study staff responsible for accountability and dispensing must not open and count clinical supplies prior to dispensing.

## 9.1 Study Drug Accountability

The site investigator and the site pharmacist are required to maintain records of the product's delivery to the site, the inventory at the site, the use by each participant, and the return to the sponsor/designee. These records will include expiration dates. Site investigators will maintain records that document adequately that the subjects were provided the appropriate prescriptions. Clinical pharmacists will maintain records that document adequately that the subjects were provided the appropriate medication, and reconcile all investigational product received from the sponsor. The site investigator must ensure that the investigational product is only prescribed in accordance with the approved protocol.

The pharmacist at the study site will receive the study agent and store it in the pharmacy. Access will be restricted to pharmacy personnel authorized by the pharmacist of record.

## 9.2 Study Drug Dispensing

The clinical site pharmacy will be responsible for dispensing the drug to each study participant upon presentation of a valid prescription. At enrolment and follow-up visits the pharmacist will receive the prescription that includes the participant's ID number. For each bottle dispensed, the pharmacist will enter the bottle label information and date on the Drug Accountability Log. Dispensation of all

study products to study staff and distribution to participants will follow the institution's SOP on Study Product Chain of Custody.

## 9.3 Study drug return/destruction

At the end of the study, the pharmacist will perform the final drug accounting of unused study material on the proper log documents. Unused study agent will be returned or disposed of as instructed by the Sponsor.

# 10.0 Biological samples

## 10.1 Laboratory supplies and sample processing

Collection and processing of biological samples for the standard of care testing procedures will be conducted according to the currently approved testing practices, and no laboratory supplies will be provided by the investigator or the sponsor for these tests.

Each study site will adhere to standards of good clinical laboratory practice, and local standard operating procedures for specimen management including proper collection, processing, labelling, transport, and storage of specimens.

### 10.1.1 PBMC, plasma and serum sample collection

Blood samples will be collected from approximately 100 participants at three time points (a total of approximately 300 collections). Additional participants may be invited to participate in blood collection if any of the participants included in the blood collection discontinue early from the study. These participants will primarily be the first 100 participants enrolled in in Sydney Sexual Health Centre, St Vincent's hospital and Holdsworth House clinic.

For participating clinics, blood will be collected for PBMCs and plasma at follow up visits 1, 3 and 5 (months 1, 6 and 12 from the baseline visit) as specified in the protocol. Approximately 16 millilitres of PBMCs and 9ml of plasma per participant follow-up visit will be collected and stored at the AMR laboratory, and bulk shipped for analysis to the Clinical Pharmacology Analytical Lab (CPAL) at the Johns Hopkins University School of Medicine, Baltimore Maryland, USA, either at the midpoint and/or end of the study follow-up period. Processing and analysis of the PBMCs and plasma will be conducted per Johns Hopkins University School of Medicine CPAL Standard Operating Procedures.

Separate informed consent will be obtained for optional serum collection and banking, to be collected at follow-up visits 1, 3 and 5 (months 1, 6 and 12 from the baseline visit) as well (see Appendix VIII). Approximately 6 millilitres of serum will be collected at each of these timepoints, and will be stored indefinitely at the St Vincent's Centre for Applied Medical Research Biobank, Sydney. Additional testing may be performed on stored samples at a later date for this, or other related research, including tests for sexually transmitted infections, viral resistance to antiretroviral drugs, and genetic factors in humans that might be associated with greater or reduced risk of getting HIV. As not all potential beneficial future research is known, the need for future research is determined by the ongoing development in the field.

For those who will not participate in the optional serum collection and banking, blood will be collected for PBMCs and plasma at the designated visits (in participating clinics only).

Due to potential variations in the follow-up schedule in cases wherein participants temporarily discontinue from the study, or discontinue and re-enrol in the study, the blood draw schedule for PBMCs, plasma and optional serum collection may similarly change to reflect these variations. Other variations include the need for post-exposure prophylaxis, additional visits to assess adherence, temporary or permanent discontinuation, pregnancy, and suspected HIV seroconversion. However, no one individual will undergo more than three research blood collection time-points during the study.

Written procedures for labelling, collection, processing, packaging and shipping will be provided to the study sites by the sponsor with support from SydPath Central Laboratory. SydPath will also provide courier services, laboratory supplies and collection kits to the sites. Both date of birth and participant name code (2 x2 code comprising first two letters of participant's first name and first two letters of participant's last name) will be captured on the blood collection tubes and requisition forms. Once the samples are processed in AMR, these additional identifiers will be removed before the samples are shipped to the CPAL at the Johns Hopkins University.

Samples will be promptly couriered per sponsor instructions to the St Vincent's Centre for Applied Medical Research (AMR) laboratory for any further processing, and for storage.

# 11.0 Data collection, source documents and record retention

## 11.1 Data collection and source documents

Attitude, behavioural and lifestyle survey data will be collected on SurveyGizmo (Boulder, Colorado, USA) via secure online forms. Data entered into the online forms will use the participants' study codes and not identifying details. The data entered into the online forms will be initially stored on SurveyGizmo's secure servers. SurveyGizmo will store the preferred email address of the participant so that the system can send participant invitation to complete the surveys and send automatic reminders if the participant does not complete a survey within the scheduled timeframe. The email address is stored separately from the participant's clinical information and his or her name is not stored in any database. Furthermore the email address will be erased from the system as soon as the participant complete his or her final follow-up survey; or when the participant withdraws his or her consent to participate in the study, whichever comes first. SurveyGizmo has detailed policies on data privacy, backup/redundancy, and disaster recovery. It is compliant and certified under both the HIPAA (US guidelines for the handling of medical information) and Safe Harbor (European Union privacy protection standards) programs. At regular intervals, these data will be downloaded from SurveyGizmo to a secure computer at the Kirby Institute. All electronic databases will be protected by password and UNSW firewalls.

Clinical study data will be collected on study specific electronic case report forms (eCRFs) in OpenClinica Enterprise. OpenClinica Enterprise complies with U.S. 21 Code of Federal Regulations Part 11, Electronic Records; Electronic Signatures. Roles and access are controlled, electronic signatures are enabled, protected health information is non-identifiable, and the database is SSL encrypted.

The site Investigator is responsible for ensuring the clinical data collected are complete, accurate and recorded in a timely manner.

Following each completed or missed participant visit the designated site staff will complete the visit specific eCRF. Any detected eCRF discrepancies will be addressed with the site staff for clarification. eCRFs must be completed, and must be signed by a site investigator in a timely manner. **Serious adverse event forms must be signed within seven calendar days of the event. All other CRFs must be signed within 14 calendar days** of the most recent completed or missed visit.

The site investigator is responsible for ensuring the completion of accurate source documentation to support the CRFs. Source documents include all recordings of observations or notations of clinical activities and all reports and records necessary for the evaluation and reconstruction of the trial. These include, but are not limited to; participant electronic and/or paper medical records, copies or transcriptions certified after verification as being accurate copies, laboratory reports, participant progress notes, pharmacy records and any other reports or records of procedures performed in accordance with the protocol or to document a serious adverse event.

Any document that acts as a source document (the point of the initial recording of a piece of data) should be signed and dated by the person recording or reviewing the data for issues of medical significance (for example the review of laboratory reports). Persons signing the source documents

must be listed as a site staff member working on the study or a staff member delegated in the delegated log.

The site investigator is responsible for retaining all essential documents listed in ICH Good Clinical Practice guidelines. These must be organised in a comprehensive filing system that is accessible to study monitors and other relevant personnel. Study records include administrative documentation, documentation of participants screened for as well as participants enrolled in the study, and all other source documents. Documents generated by a study that are not required by GCP but are required for the conduct and management of the study will also be filed.

## 11.2 Record Retention

According to the TGA requirements the sponsor will retain all study documents, including essential documents and participant files, for at least 15 years after the completion of the trial.

The sponsor will notify the sites when it is appropriate to archive study files. The site Investigator remains responsible for and should retain control of the documentation contained in the site archives, unless otherwise arranged with the sponsor and should allow the sponsor access to this documentation as required. The site will provide the sponsor with written details of a responsible contact person and the location of archived study files for ease of retrieval.

The sponsor will also inform the site investigator(s)/institution(s) in writing when the study related records are no longer needed.

## 12.0 Confidentiality of data

### 12.1 Confidentiality of participant records

By signing the Clinical Trial Agreement, the site investigator agrees that the sponsor, HREC and/or regulatory authorities may consult and/or copy study documents to verify information in the case report form. By signing the consent form the participant agrees to these processes.

Participant confidentiality in the electronic CRFs will be maintained at all times. Attitude, behavioral and lifestyle data will be collected by the sponsor using a separate electronic data collection system. No clinical documents containing the participant's name or other identifying information will be collected by the sponsor. Linkage of clinical and behavioural databases will be done using the unique study identifying numbers generated at enrolment.

It may be necessary for the sponsor's representatives, the HREC and regulatory authority representatives (e.g. TGA) to have direct access to the participant's medical records. If study documents need to be photocopied during the process of verifying case report form data, the participant will be identified by a unique code only; full names and other identifying information will be masked.

Sites are responsible for handling study data in accordance with the NSW Health Records and Information Privacy Act (HRIP Act) Statutory Guidelines on Research. All study-related information will be stored securely at the study site. All case report forms and adverse event reports will be identified by a coded number to maintain participant confidentiality. All records that contain names or other personal identifiers, such as locator forms and informed consent forms, will be stored with restricted access according to local SOPs.

Forms, lists, logbooks, appointment books, and any other listings that link participants' ID numbers to identifying information will be stored in a separate, locked file in an area with limited access.
By signing the Clinical Trial Agreement, the site investigator affirms to the sponsor that information provided to them by the sponsor will be maintained in confidence and divulged only as necessary to the ethics committee and institution employees directly involved in the study. Both ethics committee members and employees must also understand the confidentiality requirements for any information divulged to them. The data generated by this study will be considered confidential, except where it is included in a publication as agreed in the publication policy of this protocol.

# 13.0 Analyses and statistical considerations

This is a one-arm open-label treatment trial, that is no randomization and blinding procedures are used and need to be accounted for in analyses.

## 13.1 Outcome measures

Main outcome measures will be:

- time to accrual of 300 person years of follow-up on TRUVADA
- Seroconversion-free time on PrEP
- Time to discontinuation
- Percentage of prescribed doses taken orally in the prescribed period
- Incidents of HIV
- Incidents of rectal Gonorrhea and Chlamydia
- Serious adverse reactions
- Any adverse events leading to interruption or discontinuation of the study product (TRUVADA)
- All study endpoints are described in Section 5.0.

## 13.2 Laboratory analyses

The following analyses will be conducted as a standard of care for population groups at high risk of HIV in Australia:

- Laboratory analyses to confirm new HIV infection will allow the detection of the main study endpoint (i.e. seroconversion in PrEP users).
- Laboratory analyses to identify incident cases of gonorrhea and chlamydia. Both are known to be STI co-factors in HIV epidemic in Australia [42] and are also markers of risk, and thus a biological measure of behavioural disinhibition
- Laboratory analyses of liver and renal functions
- Laboratory analyses of STI testing, and of Hepatitis B and C infection
- Pregnancy testing for all women of child-bearing potential

## 13.3 Sample size

The study target is to recruit at least 300 participants *and* accrue 300 person-years of follow-up on TRUVADA. This is an implementation project; the sample size is not based on power calculations but is a pragmatic choice to evaluate the process and outcomes of limited off-label TRUVADA® use for purposes of primary HIV prevention. This sample size is based on the evidence of PrEP use and expression of interest to use PrEP in community-based observational studies conducted in Australia in 2012-2013 and on the number of participants that is feasible to recruit through the participating public clinics in NSW, Australia.

## 13.4 Statistical analyses

The analyses will be described in detail in a full Statistical Analysis Plan. Briefly:

***Statistical analyses of sexual practices*** will include an examination of current awareness of HIV risk factors and prevention strategies, sexual practices, condom use, TRUVADA® use and adherence to daily medication schedule. Analyses will also include comparison of sexual practices over time during the course of treatment and up to six months after treatment discontinuation (at three and six months after completion of the treatment follow-up).

***Statistical analyses of adherence to the study medication*** will focus on assessing time of TRUVADA, number and proportion of missed doses, the relationship between sexual practices and medication use, and the relationship between adherence levels and HIV-free time on the study product. A sub-study may be submitted as a future amendment to include the collection and assessment of blood plasma levels of TDF/FTC.

***Statistical analyses of the risk of HIV seroconversion*** among individuals on PrEP will focus on estimating the risk of seroconversion per person-time of follow-up on TRUVADA, the probability of HIV seroconversion per sexual contact among individuals taking PrEP (Quinn et al 2000; Jin, Jansson et al 2010), and the effect modifying role of non-adherence to the prescribed medication schedule, STI and side-effects leading to inconsistent PrEP use. Analyses of HIV seroconversions will also include the period of three months after completion of the study treatment using data linkage with the NSW HIV registry.

Analyses will be conducted using appropriate longitudinal regression models with time-varying exposures. Data analysis will be conducted by a research officer at the Kirby Institute, under the leadership of the Principal and Investigator and Co-Investigators, using STATA (StataCorp, College Station, TX, USA). The research team has extensive expertise in the analyses of longitudinal data.

## 13.5 Interim monitoring and analysis

Members of the Study Management Team (SMT) will conduct an interim analysis of the results when first 100 participants are recruited. Analyses will focus on safety and adherence to the study medication. The results of the interim analysis will be reported to the Protocol Steering Committee (PSC) and Data Safety and Monitoring Board (DSMB). The DSMB will review medication adherence data at this time, to determine whether there is a need to develop further approaches to medication adherence interventions. Interim results may also be reported to the New South Wales Ministry of Health and back to community organisations, participants and the community more generally.

# 14.0 Ethics committee/regulatory approval and informed consent

## 14.1 Ethics committee/regulatory approval

This study will be conducted under the TGA Clinical Trial Notification (CTN) scheme. The sponsor will arrange regulatory notification for the study prior to its start.

Under the CTN scheme, the Approving Authority is the institution that grants the final approval for the conduct of the study. Each site's investigator is responsible for obtaining HREC approval for the protocol and PICF in compliance with local regulatory requirements prior to participant enrollment.

The site investigator may not implement any deviation from, or changes to the protocol without agreement by the sponsor and prior review and documented approval/favourable opinion from the HREC of an amendment, except where necessary to eliminate an immediate hazard(s) to study participants, or when the change(s) involves only logistical or administrative aspects of the study (e.g. change in monitor(s), change of telephone number(s)). The site investigator must also obtain approval for any amendments to the participant information and informed consent form (PICF) documents. The site investigator must comply with all HREC reporting requirements for all adverse events, periodic updates and end of study reports and must agree to abide by any HREC conditions of approval.

## 14.2 Informed consent

The site investigator (or equivalent trained designee) is responsible for ensuring freely-given written informed consent is obtained from each potential participant prior to the conduct of any protocol-specific study procedures. The site investigator may delegate the task of obtaining consent to appropriately qualified Sub-investigator(s). The participant must be informed in a timely manner of any new information that becomes available during the course of the study that may affect his or her willingness to continue study participation. Additional informed consent requirements are outlined in section 7.1.2 Informed consent process.

This study shall be conducted in accordance with the ethical principles laid out in the World Medical Association Declaration of Helsinki (most current issued version), the CPMP/ICH *Note for Guidance on Good Clinical Practice* (CPMP/ICH/135/95), the National Statement on Ethical Conduct in Research Involving Humans (2007), the requirements of the Therapeutic Goods Administration as outlined in this document; and any requirements of relevant Commonwealth and/or State laws.

## 15.0 Legal Issues

The sponsor will consult throughout the study with the University of New South Wales Australia legal team when necessary to address any arising legal issues related to the study implementation and will deal with such issues in an appropriate and timely manner, in accordance with the local and regulatory requirements.

The sponsor has requested and obtained legal advice from the NSW HIV/AIDS Legal Centre (HALC) about risk to participants in this study and has incorporated the legal advice in the design and procedures of this PrEP implementation study.

# 16.0 Governance

This research protocol is funded by NSW Department of Health. The study medication (TRUVADA) will be provided by Gilead Inc. The study is based at the University of New South Wales (UNSW) and coordinated through the Kirby Institute for Infection and Immunity in Society. The Kirby Institute has established governance and implementation structures which use resources efficiently to deliver program objectives on schedule.

Each participating clinical site within a public health organisation will obtain a site-specific assessment (SSA) for this study. HREC approval is required prior to SSA submission.

This study will be registered in the Australian New Zealand Clinical Trials Registry and the U.S. National Library of Medicine clinicaltrials.gov registry.

## 17.0 Financing and insurance

In the event of study-related injury, study participants and staff will be covered under the Certificate of Currency – Clinical Trials Insurance policy for UNSW including Kirby Institute, which meets the required indemnity limits.

## 18.0 Quality Control (QC) and Quality Assurance (QA)

### 18.1 Quality control

The sponsor is responsible for implementing and maintaining quality control and quality assurance systems with written standard operating procedures to ensure the study is conducted and data are generated, documented and reported in compliance with the protocol, Good Clinical Practice standards and all applicable local laws and regulations relating to the conduct of a clinical trial.

The study will be conducted in full compliance with the protocol. The protocol will not be amended without prior written approval by the sponsor. All protocol amendments must be submitted to and approved by the HREC prior to their implementation.

On behalf of the sponsor, study monitors will visit each site periodically to ensure the study is conducted, recorded, and reported in accordance with the protocol, Standard Operating Procedures, GCPs, and applicable regulatory requirements. Monitors will also confirm the quality and accuracy of study data and documentation, and verify proper storage, dispensing, and accountability of the investigational study product.

The frequency and number of monitoring visits will depend upon the recruitment rate as defined in the monitoring plan. The monitor will conduct a monitoring visit within two weeks of each SUSAR or HIV conversion.

### 18.2 Protocol deviation and Violation

The following definitions and reporting requirements apply.

- A **protocol deviation** occurs when, without significant consequences, the activities on a study diverge from the approved protocol, e.g., missing a visit window because the subject is traveling. A protocol deviation presented in advance of the event may be considered acceptable by the sponsor and the HREC.

- A **protocol violation** is a divergence from the protocol that reduces the quality or completeness of the data, makes the participant information sheet/informed consent form inaccurate, or impacts a subject's safety, rights, or welfare. Examples of protocol violations may include the following:
    - Inclusion/exclusion criteria not met
    - Unreported serious adverse events
    - Use of prohibited medication
    - Intentional deviation from protocol, Good Clinical Practice, or regulations by study personnel
    - Subject repeated non-compliance with study requirements

Protocol deviations and violations will be detailed in the monitoring reports. Sites should report to the sponsor **within two working days** of discovery, any protocol violations wherein inclusion/ exclusion criteria were not met, serious adverse events were not reported, or the subject's safety, rights or welfare were affected.

# 19.0 Study oversight and management committees

## 19.1 Study Management Team

The Prelude Study Management Team is responsible for ongoing study management, coordination and implementation. It includes the study investigators and personnel based at the HIV Epidemiology and Prevention Program at The Kirby Institute.

## 19.2 Protocol Steering Committee

The Protocol Steering Committee (PSC) is responsible for prioritizing research topics, developing protocols, facilitating and monitoring the conduct of the research, and reporting study results in a timely manner. The PSC conducts its business through periodic meetings (at least annually). The PSC includes a subset of investigators as well as representatives of key community organisations. Other investigators and site coordinators may also participate in these meetings.

## 19.3 Data Safety and Monitoring Board

The Data Safety and Monitoring Board (DSMB) is responsible for advising the project team on the conduct and progress of the study, the quality of data generated, and issues regarding participant safety. The DSMB may recommend study modification, suspension or termination, and will consider any severe unanticipated problems with any aspect of the study, including concerns for patient welfare or scientific integrity. The study team and the Protocol Steering Committee (PSC) will implement the recommendations of the DSMB.

On-going review during the trial will occur after completed follow-up of the first 100 patients and at completion of the study. HIV seroconversion rates, STI rates, safety events and risk behaviour will be examined. At each review, the DSMB will recommend to the PSC one of the following courses of action:

- Continue the study without modification
- Pause enrolment pending either resolution of specific issues or amendment of the protocol as specified, or
- Terminate the study.

The DSMB will give serious consideration to recommending termination of the study in the event of:

- An increase in the number of Serious Adverse Events (including HIV seroconversions and positive STI test results) to a level considered unacceptable by the DSMB
- Any increase in the adverse events raising concerns of participant safety.

There will be at least five members on the DSMB. The specific members are nominated by the PSC. Membership will include, at a minimum:

- A representative of the HIV-positive community
- A statistician
- An expert in clinical trial design and conduct and/or in human research ethics
- Two physicians who are experts in HIV clinical care

The DSMB meets at least annually for the duration of the study in accordance with the DSMB charter. All data presented at the DSMB meeting are confidential.

## 19.4 Medical Officer

The PRELUDE study medical officer is responsible for safety monitoring and review. He or she will ensure appropriate serious event review and reporting procedures. The Medical Officer will review all serious adverse events for assessments of relatedness and expectedness, and will advise the protocol team whether the event meets the requirements for expedited reporting and/or further action to other parties.

## 20.0 Publication Policy

Authorship of publications arising from PRELUDE Study must conform to the standards of the meeting or journal where the research findings will be reported.

All investigators named on the study protocol will be invited to be an author on each paper, as will any other investigators/individuals that make a substantial contribution. The invited investigators/individuals are free to make their own decisions whether to become authors on any paper arising from the study. However, as recommended in the authorship considerations proposed by the International Committee of Medical Journal Editors, those who make a written statement that they meet *each* of the following conditions will be included as a co-author:

i) Substantial contributions to conception and design, or acquisition of data, or analysis and interpretation of data; ***and***
ii) Drafting the article or revising it critically for important intellectual content; ***and***
iii) Final approval of the version to be published.

All prospective authors of all publications will be notified of publication plans in sufficient time to participate fully in authorship or otherwise to have input into the content and review of the manuscript.

Authorship order will depend on the relative contribution of the individual authors. Procedures for deciding order of authorship should be developed by consensus of the authors at the earliest appropriate time in the development of the manuscript or presentation. In general, the first named author will be the individual who writes the manuscript/presentation. In general, the senior (last) author will be a senior investigator who has expertise in the subject matter of the manuscript/presentation, and who has closely supervised the writing of the manuscript/presentation.

Acquisition of funding, collection of data or general supervision of the research group alone does not constitute authorship.

If the number of people meeting the journal's or meeting's criteria for authorship is greater than the journal or meeting standards allow, a collective authorship designation may be used if allowed by the journal or meeting. The specific designation will be decided by consensus of the authors and study investigators. If a collective authorship is used, the persons responsible for the publication or presentation (i.e. those who otherwise would have been individually named as authors) will be specified in a manner agreed to by the journal or meeting.

At an appropriate place in the publication or presentation, as consistent with the standards of the journal or meeting, one or more statements should specify:

i) Acknowledgment of contributions that do not justify authorship, including technical help and financial or material support; and
ii) Financial relationships that may constitute a perceived conflict of interest.

Each person acknowledged by name should give permission in writing or by email to be acknowledged. Exceptions may be made if the person is deceased or cannot be contacted.

This policy applies regardless of the organisation or institution of the investigator responsible for drafting the publication or presentation.

A copy of this policy will be included in all PRELUDE Study sub-agreements, sub-contracts, sub-grants, or sub-study agreements.

This policy also encompasses the publication and presentation of data from sub-studies.

## 21.0 References


1. Grant RM, Lama JR, Anderson PL, et al. Preexposure chemoprophylaxis for HIV prevention in men who have sex with men. *NEnglJMed* 2010; **363**(27): 2587-99.
2. Baeten JM, Donnell D, Ndase P, et al. Antiretroviral Prophylaxis for HIV Prevention in Heterosexual Men and Women. *New Engl J Med* 2012; **367**(5): 399-410.
3. Choopanya K, Martin M, Suntharasamai P, et al. Antiretroviral prophylaxis for HIV infection in injecting drug users in Bangkok, Thailand (the Bangkok Tenofovir Study): a randomised, double-blind, placebo-controlled phase 3 trial. *Lancet* 2013; **381**(9883): 2083-90.
4. Thigpen MC, Kebaabetswe PM, Paxton LA, et al. Antiretroviral Preexposure Prophylaxis for Heterosexual HIV Transmission in Botswana. *New Engl J Med* 2012; **367**(5): 423-34.
5. Liu AY, Hessol NA, Vittinghoff E, et al. Medication adherence among men who have sex with men at risk for HIV infection in the United States: implications for pre-exposure prophylaxis implementation. *AIDS patient care and STDs* 2014; **28**(12): 622-7.
6. McCormack S, Dunn DT, Desai M, et al. Pre-exposure prophylaxis to prevent the acquisition of HIV-1 infection (PROUD): effectiveness results from the pilot phase of a pragmatic open-label randomised trial. *Lancet* 2015.
7. Molina JM and Delfraissy JF. On Demand PrEP With Oral TDF-FTC in MSM: Results of the ANRS Ipergay Trial. CROI 2015. Oral late breaker abstract 23LB. Available at: http://www.croiconference.org/sessions/demand-prep-oral-tdf-ftc-msm-results-anrs-ipergay-trial.
8. NSW Government MoH. NSW HIV strategy 2012-2015: A new era. *Sydney, NSW, 2012* 2012.
9. Gilead. Truvada: Medication Guide #21-752-GS-026. https://www.gilead.com/~/media/Files/pdfs/medicines/hiv/truvada/truvada_pi.pdf; June 2013.
10. Administration USFaD. FDA approves firstdrug for reducing the risk of sexually acquired HIV infection. 2013.
11. Grant RM, Anderson PL, McMahan V, et al. Uptake of pre-exposure prophylaxis, sexual practices, and HIV incidence in men and transgender women who have sex with men: a cohort study. *Lancet Infect Dis* 2014; **14**(9): 820-9.
12. Gallant JE, Dejesus E, Arribas JR, et al. Tenofovir DF, emtricitabine, and efavirenz vs. zidovudine, lamivudine, and efavirenz for HIV. *NEnglJMed* 2006; **354**(3): 251-60.
13. Hawkins T, Veikley W, St Claire RL, Guyer B, Clark N, Kearney BP. Intracellular pharmacokinetics of tenofovir diphosphate, carbovir triphosphate, and lamivudine triphosphate in patients receiving triple-nucleoside regimens. *JAcquirImmuneDeficSyndr* 2005; **39**(4): 406-11.
14. Vourvahis M, Tappouni HL, Patterson KB, et al. The pharmacokinetics and viral activity of tenofovir in the male genital tract. *JAcquirImmuneDeficSyndr* 2008; **47**(3): 329-33.
15. Anderson PL, Glidden DV, Liu A, et al. Emtricitabine-tenofovir concentrations and pre-exposure prophylaxis efficacy in men who have sex with men. *Sci Transl Med* 2012; **4**(151): 151ra25.
16. Amico KR. The key role of adherence for the effectiveness of antiretroviral-based prevention: State of the science and implications for Asia Pacific. *Sexual Health* 2014.
17. Prevention CfDCa. Update to interim guidance for pre-exposure prophylaxis (PrEP) for the prevention of HIV infection: PrEP for injecting drug users. *Morb Mortal Wkly Rep* 2013; **62**.
18. Baeten JM, Donnell D, Ndase P, et al. Antiretroviral prophylaxis for HIV prevention in heterosexual men and women. *The New England journal of medicine* 2012; **367**(5): 399-410.
19. Van Damme L, Corneli A, Ahmed K, et al. Preexposure prophylaxis for HIV infection among African women. *The New England journal of medicine* 2012; **367**(5): 411-22.

20. Marrazzo J, Ramjee, G, Nair, G, Palanee, T, Mikhize, B, Nakabiito, C, Taljaard, M, Piper, J, Gomez Feliciano, K. Pre-exposure prophylaxis for HIV in women: Daily oral tenofovir, oralntenofovir/emtricitabine, or vaginal tenofovir gel in the VOICE study (MTN 003) (Papern #26LB). Oral abstract presentation. CROI 2013. 2014.
21. Kibengo FM, Ruzagira E, Katende D, et al. Safety, adherence and acceptability of intermittent tenofovir/emtricitabine as HIV pre-exposure prophylaxis (PrEP) among HIV-uninfected Ugandan volunteers living in HIV-serodiscordant relationships: a randomized, clinical trial. *Plos One* 2013; **8**(9): e74314.
22. Mugo PM, Sanders EJ, Mutua G, et al. Understanding Adherence to Daily and Intermittent Regimens of Oral HIV Pre-exposure Prophylaxis Among Men Who Have Sex with Men in Kenya. *AIDS and behavior* 2015; **19**(5): 794-801.
23. Mutua G, Sanders E, Mugo P, et al. Safety and Adherence to Intermittent Pre-Exposure Prophylaxis (PrEP) for HIV-1 in African Men Who Have Sex with Men and Female Sex Workers. *Plos One* 2012; **7**(4).
24. Liu A, Glidden DV, Anderson PL, et al. Patterns and correlates of PrEP drug detection among MSM and transgender women in the Global iPrEx Study. *Journal of acquired immune deficiency syndromes* 2014; **67**(5): 528-37.
25. World Health Organization. Guidance on oral pre-exposure prophylaxis (PrEP) for serodiscordant couples, men and transgender women who have sex with men at high risk of HIV. July 2012. Available online at: http://www.who.int/hiv/pub/guidance_prep/en/.
26. Anderson PL, Kiser JJ, Gardner EM, Rower JE, Meditz A, Grant RM. Pharmacological considerations for tenofovir and emtricitabine to prevent HIV infection. *JAntimicrobChemother* 2010.
27. Mugwanya KK, Donnell D, Celum C, et al. Sexual behaviour of heterosexual men and women receiving antiretroviral pre-exposure prophylaxis for HIV prevention: a longitudinal analysis. *The Lancet infectious diseases* 2013; **13**(12): 1021-8.
28. Volk JE, Marcus JL, Phengrasamy T, et al. No New HIV Infections With Increasing Use of HIV Preexposure Prophylaxis in a Clinical Practice Setting. *Clin Infect Dis* 2015.
29. Poynten IM, Jin F, Prestage GP, Kaldor JM, Imrie J, Grulich AE. Attitudes towards new HIV biomedical prevention technologies among a cohort of HIV-negative gay men in Sydney, Australia. *HIVMed* 2010; **11**(4): 282-8.
30. Zablotska I, GP P, de Wit JB, Mao L, AE G, Holt M. The informal use of antiretrovirals for pre-exposure prophylaxis of HIV among Australian gay men. *J AcquirImmuneDeficSyndr* 2012; **22 November 2012**({Epub Ehead of print]).
31. Holt M, Murphy DA, Callander D, et al. Willingness to use HIV pre-exposure prophylaxis and the likelihood of decreased condom use are both associated with unprotected anal intercourse and the perceived likelihood of becoming HIV positive among Australian gay and bisexual men. *Sex TransmInfect* 2012; **88**(4): 258-63.
32. Holt M. HIV pre-exposure prophylaxis and treatment as prevention: a review of awareness and acceptability among men who have sex with men in the Asia-Pacific region and the Americas. *Sexual Health* 2014; **11**(2): 166-70.
33. Aging AGDoHa. 6th National HIV Strategy (2010-2013).
34. Zablotska I (on behalf of the NSW PrEP guidelines and clinical delivery group). Pre-Exposure Prophylaxis of HIV with antiretroviral medications: Interim NSW guidelines. 10 October 2014. Availalbe online: https://preludestudy.files.wordpress.com/2015/02/nsw-guidelines-on-prep1.pdf.
35. ASHM. Australian natinal PrEP guidelines - 2015. Available online:http://arv.ashm.org.au/arv-guidelines/prep-resources-for-clinicians. Last accessed on 2 March 2015.
36. D'Anna LH, Margolis AD, Warner L, et al. Condom use problems during anal sex among men who have sex with men (MSM): findings from the Safe in the City study. *AIDS Care* 2012; **24**(8): 1028-38.
37. Yin MT, Overton ET. Increasing Clarity on Bone Loss Associated With Antiretroviral Initiation. *J Infect Dis* 2011; **203**(12): 1705-7.

38. Liu AY, Vittinghoff E, Sellmeyer DE, et al. Bone mineral density in HIV-negative men participating in a tenofovir pre-exposure prophylaxis randomized clinical trial in San Francisco. *PLoSOne* 2011; **6**(8): e23688.
39. Panel_on_Antiretroviral_Guidelines_for_Adults_and_Adolescents. Guidelines for the use of antiretroviral agents in HIV-1-infected adults and adolescents. *Department of Health and Human Services Available at http://aidsinfonihgov/ContentFiles/AdultandAdolescentGLpdf Last accessed on 9 April 2014*.
40. ASHM. 2013 Antiretroviral Guidelines with Australian Commentary. *Available online at http://arvashmorgau/ Last accessed on 9 April 2014*.
41. ASHM. National guidelines for post-exposure prophylaxis after non-occupational exposure to HIV. *Australian Government Department of Health and Aging*, 2007, 2007. http://www.ashm.org.au/images/publications/guidelines/2007nationalnpepguidelines2.pdf (accessed.
42. Jin F, Prestage GP, Zablotska I, et al. High rates of sexually transmitted infections in HIV positive homosexual men: data from two community based cohorts. *Sex TransmInfect* 2007; **83**(5): 397-9.

## 22.0 *Attachments/Appendices

Appendix I TRUVADA Product Information, v.13 (23 October 2013)

Appendix II HIV incidence associated with different risk practices in homosexual men

Appendix III

Box A: Algorithm for identifying homosexual men at risk for acquiring HIV infection

Box B: Algorithm for identifying heterosexual women at risk for acquiring HIV infection

Appendix IV Expected toxicities/adverse events associated with TRUVADA

Appendix V Study process flowchart

Appendix VI Template Pre-screening Participant Information and Consent Form

Appendix VIIA Template PRELUDE Participant Information and Consent Form (with blood collection)

Appendix VIIB Template PRELUDE Participant Information and Consent Form (without blood collection)

Appendix VIII Template PRELUDE Blood Banking Participant Information and Consent Form

***Disclaimer:** *Templates attached to this document are usually a 'Master Template" of the preferred format, which can be adapted by clinical sites, as appropriate, for site-specific versions of these documents. The final version of any document requires approval by the ethics committee prior to being implemented. Exceptions to this would include an official document template (such as a specific version of a questionnaire) being used, for which the requirements have been revised and a new version has been issued – then an amendment to the protocol must be submitted to the ethics committee for review and approval.*

Appendix I: TRUVADA Product Information, v.13 (23 October 2013)

https://www.ebs.tga.gov.au/ebs/picmi/picmirepository.nsf/PICMI?OpenForm&t=&q=emtricitabine

Appendix II: HIV incidence associated with different risk practices in homosexual men

*Note: Data for this table were obtained from the Health in Men (HIM) study conducted during 2001-2007. Data were collected for six month intervals. Due to the specifics of data collection for this study, not all indicators were available to support each individual eligibility criterion, and some indicators were collected in somewhat different form, have a different denominator or reference period.*

| **Risk factor** | **Unadjusted HIV incidence** |
|---|---|
| All patients regardless of practices | 0.78 per 100 PY<br>95% CI (0.59-1.02) |
| **A. Very high risk: recommend prescribing daily PrEP** | |
| A regular sexual partner of or having at least ***one*** episode of unprotected sex with an HIV-infected man with whom condoms were not consistently used in the last six months[1] | 5.36 per 100 PY<br>95% CI (2.78-10.25)* |
| At least ***one*** episode of receptive unprotected anal intercourse (UAI) with any casual HIV-infected male partner or a male partner of unknown HIV status during the last six months | 2.31 per 100PY<br>95% CI (1.48-3.63) |
| Rectal gonorrhoea diagnosis in last six months | 7.01 per 100PY<br>95% CI(2.26-21.74) |
| Rectal chlamydia diagnosis in last six months | 3.57 per 100PY<br>95% CI (1.34-9.52) |
| Methamphetamine use in last six months | 1.89 per 100PY<br>95% CI (1.25-2.84) |
| **B. Medium to high risk: consider prescribing daily PrEP** | |
| More than one episode of anal intercourse during the last six months when proper condom use was not achieved (e.g., condoms slipped off or broke) | 1.30 per 100 PY<br>95% CI (0.95-1.77) |
| A regular sexual partner of or having at least ***one*** episode of insertive UAI where the serostatus of partner was not known or was HIV positive in the last six months[2] | 0.94 per 100 PY<br>95% CI (0.35-2.52)* |
| - In circumcised men | 0.65 per 100PY<br>95% CI (0.16-2.61) |
| - In uncircumcised men | 1.73 per 100PY<br>95% CI (0.43-6.90) |

[1] Data used to generate this estimate did not include the treatment and viral load status of the HIV positive regular partner as this information was not available
[2] The estimates produced by the HIM study cannot account for the treatment and/or viral load status of the HIV positive regular partner as this information was not collected

Appendix III: Algorithms for determining eligibility for study participation

**BEHAVIOURAL ELIGIBILITY CRITERIA FOR PREP FOR HOMOSEXUAL MEN**

**A. - High risk - Recommend prescribing daily PrEP if the client acknowledges:**

being likely to have multiple events of unprotected anal intercourse (UAI), with or without sharing IDU in the next 3 months (indicating sustained risk)

AND

***Having any of the following:***

- Regular sexual partner of an HIV-infected man with whom condoms were not consistently used in the last 3 months (HIV positive partner is not on treatment and/or has detectable viral load);
- At least ***one*** episode of receptive UAI with any casual HIV-infected male partner or a male partner of unknown HIV status during the last 3 months;
- Rectal gonorrhoea or chlamydia diagnosis during the last 3 months or at screening;
- Methamphetamine use in the last 3 months.

**B. Medium risk - Consider prescribing daily PrEP if the client acknowledges:**

being likely to have multiple events of UAI with or without sharing IDU, in the next 3 months (indicating sustained risk)

AND

***Any of the following is reported:***

- More than one episode of anal intercourse in the last 3 months when proper condom use was not achieved (e.g., condoms slipped off or broke);
- If client is uncircumcised and reports more than one episode of insertive UAI in the last 3 months where the serostatus of partner was not known or was HIV positive and not on treatment.

**C. Low risk - PrEP is not recommended for individuals who:**

- Have no risk exposure other than UAI with a partner with documented sustained undetectable HIV viral load in the last 3 months;
- Are circumcised and report practicing exclusively insertive UAI in the last 3 months

***Note**: Homosexual men seeking PrEP for use within serodiscordant partnerships will be eligible for the PRELUDE Study until evidence is published that shows undetectable viral load is protective. If this criterion changes, the sponsor will notify each site in writing immediately, and will follow up with a protocol amendment.*

Appendix III Box B: Algorithms for identifying heterosexual men and women at risk for acquiring HIV infection

**BEHAVIOURAL ELIGIBILITY CRITERIA FOR PREP FOR HETEROSEXUAL WOMEN**

**A. High risk - recommend prescribing daily PrEP if the client acknowledges:**
being likely to have multiple events of unprotected vaginal or anal intercourse (UVI or UAI, respectively), with or without sharing IDU, in the next 3 months (indicating sustained risk)

AND

- Being a regular sexual partner of an HIV-infected man with whom condoms were not consistently used in the last 3 months (HIV positive partner is not on treatment and/or has detectable viral load);

**B. Medium risk - consider prescribing daily PrEP if:**

- a female client is in serodiscordant heterosexual relationship and is planning natural conception in the next 3 months

**C. Low risk - PrEP is not recommended for individuals who:**

- have no risk exposure other than UVI or UAI with a partner with documented sustained undetectable HIV viral load in the previous 3 months. However, PrEP may be considered for a female client during a period around attempted conception.

Appendix IV: Expected toxicities/adverse events associated with TRUVADA

**Common Adverse Events**

- In HIV-1–uninfected individuals in PrEP trials, adverse reactions that were reported by more than 2% of TRUVADA® subjects and more frequently than by placebo subjects were headache, abdominal pain, and weight decreased.

- The most common adverse events (incidence ≥10%) reported by HIV-1–infected subjects in clinical trials (in combination with efavirenz) are diarrhea, nausea, fatigue, headache, dizziness, depression, insomnia, abnormal dreams, and rash.

The following side effects have been associated with the use of **emtricitabine**:

- Headache
- Dizziness
- Tiredness
- Inability to sleep, unusual dreams
- Loose or watery stools
- Upset stomach (nausea) or vomiting
- Abdominal pain
- Rash, itching, which sometimes can be a sign of an allergic reaction
- Skin darkening of the palms and/or soles
- Increased cough
- Runny nose
- Abnormal liver function tests, which could mean liver damage
- Increases in pancreatic enzyme (substances in the blood), which could mean a problem with the pancreas
- Increased triglycerides
- Increased creatinine phosphokinase, which could mean muscle damage

The following side effects have been associated with the use of **tenofovir**:

- Upset stomach, vomiting, gas, loose or watery stools
- Generalized weakness
- Dizziness
- Depression
- Headache
- Abdominal pain
- Worsening or new kidney damage or failure
- Inflammation or swelling and possible damage to the pancreas and liver
- Shortness of breath
- Rash
- Allergic reaction: symptoms may include fever, rash, upset stomach, vomiting, loose or watery stools, abdominal pain, achiness, shortness of breath, a general feeling of illness or a potentially serious swelling of the face, lips, and/or tongue
- Bone pain and bone changes such as thinning and softening which may increase the risk of breakage
- Muscle pain and muscle weakness

**Serious side effects per TRUVADA prescribing information:**

- Lactic acidosis (elevated lactic acid levels in the blood) and severe hepatomegaly (enlarged liver) with steatosis (fatty liver) that may result in liver failure, other complications or death have been reported with the use of antiretroviral nucleoside analogues alone or in combination. The liver complications and death have been seen more often in women on these drug regimens. Some nonspecific symptoms that might indicate lactic acidosis include: unexplained weight loss, stomach discomfort/pain, nausea, vomiting, fatigue, cramps, muscle pain, weakness, dizziness or lightheadedness, shortness of breath, cold or blue hands and feet, and fast or abnormal heartbeats.

- **Severe liver problems.** In some cases these liver problems can lead to death. Symptoms include: jaundice, dark "tea-colored" urine, light-colored stools, loss of appetite for several days or longer, nausea, stomach-area pain.

- **Worsening of hepatitis B virus (HBV) infection if TRUVADA® is stopped.**

Appendix V: Study process flowchart

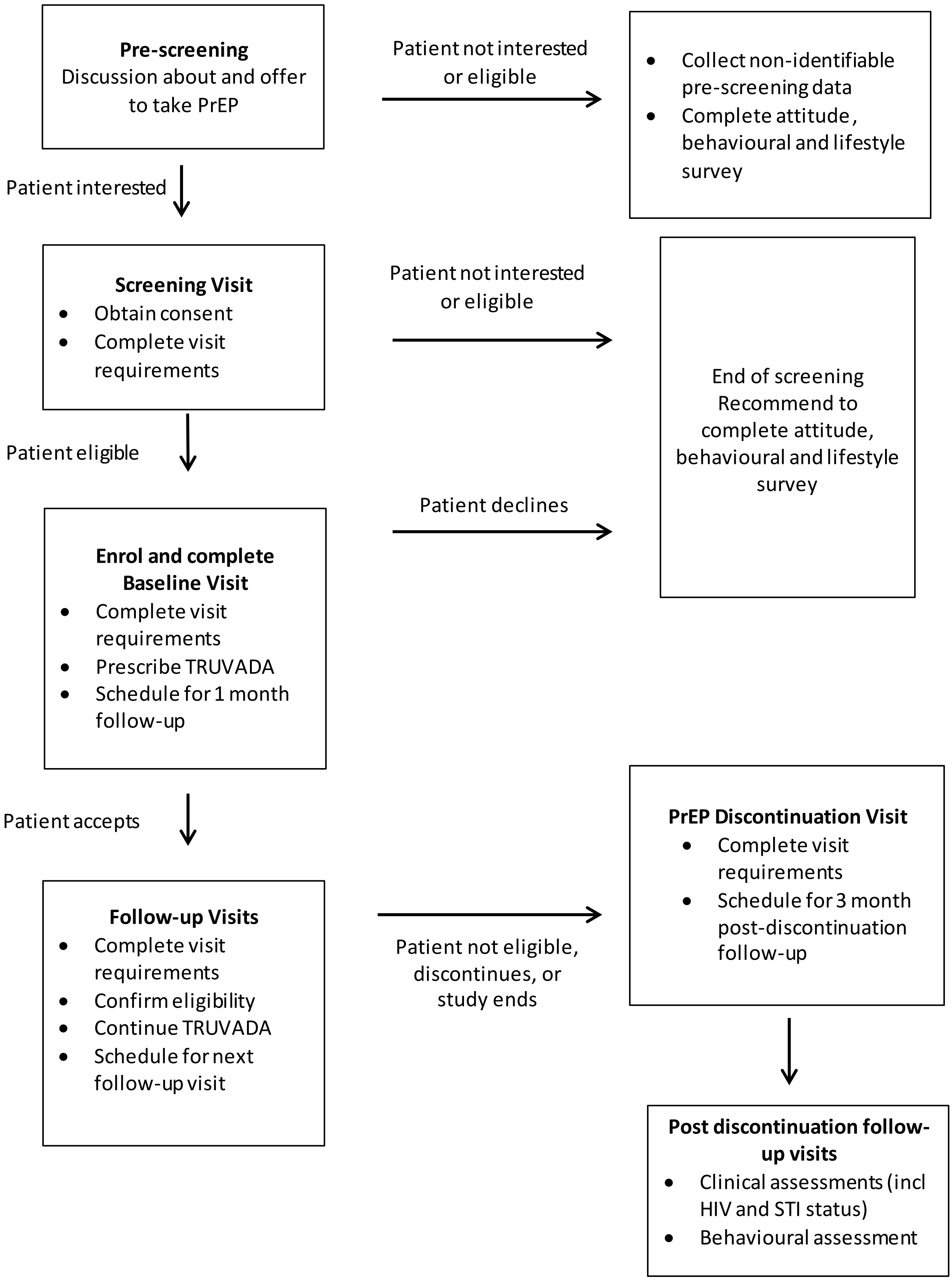

*Site: Insert Header with institution's name or institution's letterhead as required*

# Participant Information Sheet/Consent Form
# PRELUDE Study Pre-screening

*[Insert site name if required]*

| | |
|---|---|
| **Title** | Pre-screening for implementation of HIV preexposure prophylaxis with antiretroviral medications among people at high risk for HIV infection: A demonstration project |
| **Short Title** | The PRELUDE Study pre-screening |
| **Protocol Version** | 15 August 2014 |
| **Protocol Number** | HEPP 1403 |
| **Project Sponsor** | The Kirby Institute for Infection and Immunity in Society |
| **Coordinating Principal Investigator** | Dr. Iryna Zablotska, The Kirby Institute |
| **Associate Investigator(s)** | *[Site: insert if required by institution]* |

## Part 1: What does my participation involve?

## 1 Introduction

You are invited to provide pre-screening information to see if you may qualify for a research project called the PRELUDE Study. You are invited because you say you are HIV-negative and may be at increased risk of getting HIV (Human Immunodeficiency Virus). HIV is the virus that causes Acquired Immunodeficiency Syndrome, or AIDS. In the PRELUDE research project, a medicine that has been used to treat people who *have* HIV will be given to HIV-negative people to evaluate its use as the means to *lower* their chance of getting HIV. This is called preexposure prophylaxis or 'PrEP.' Prophylaxis means doing something to prevent an illness or infection.

This Participant Information Sheet/Consent Form (PICF) tells you about the pre-screening process for the PRELUDE Study. Knowing what is involved will help you decide if you want to pre-screen for the study. There is a separate Participant Information Sheet/Consent Form for the PRELUDE Study, which explains all of the procedures, risks, and benefits associated with PrEP and the PRELUDE Study research.

Please read this information carefully. Ask questions about anything that you don't understand or want to know more about. Before deciding whether or not to take part, you might want to talk about it with a friend, partner, family member or your doctor.

Participation in this pre-screening is voluntary. If you don't wish to take part, you don't have to. You will receive the same quality of care whether or not you take part.

If you decide you want to pre-screen for the PRELUDE research project, you will be asked to sign the consent section of this document. By signing it you will be telling us that you:

- Understand what you have read
- Consent to answer the pre-screening questions for the PRELUDE Study
- Consent to complete an on-line survey about attitudes, behavioural and lifestyle as described.
- Consent to the use of your personal and health information as described.

You will be given a copy of this Participant Information and Consent Form to keep.

## 2 What is the purpose of this pre-screening process?

The PRELUDE Study will evaluate a new additional way to lower people's chances of getting HIV and will enrol participants who meet certain criteria. The purpose of the pre-screening for the PRELUDE Study is to see whether you meet certain criteria as related to your risk of getting HIV through sex. The purpose of completing the on-line survey questions is to collect basic information about why you want to take PrEP, your attitudes regarding PrEP and HIV, and your risk behaviours.

This research study is being conducted by a group of investigators at the Kirby Institute, the University of New South Wales, and lead by Dr. Iryna Zablotska.

## 3 What does participation in this pre-screening involve?

The pre-screening process may be completed before or on the same day as the PRELUDE Study screening visit.

The pre-screening consent form must be signed by you before you are asked the HIV risk questions, and before you complete the on-line attitude, behaviour and lifestyle survey.

**At the clinic**

For this pre-screening process, doctors who are also researchers on this study will make sure you are a good candidate for PrEP. You will be asked approximately ten questions to see if you are at increased risk of getting HIV from sex. The doctor will then give you a unique identification number, a website address and a user name, for you to use to complete an on-line survey.

In order to qualify for the pre-screening, you must:

- Be at least 18 years old
- Live in NSW, or visit NSW enough to attend follow-up visits in the PRELUDE Study.
- Be willing to take a computer-based survey about your attitudes, behaviours and lifestyle
- Be willing and able to consent to pre-screening.

**After the pre-screening clinic visit**

You will be asked to use your unique identification number to complete a self-administered on-line survey in a location of your choice (e.g. at home), ideally within two and no more than seven calendar days of the clinic visit. If you qualify and start to screen for the PRELUDE Study, you will have to complete the pre-screening survey before you start taking the PrEP medication.

The survey will ask questions about why you want to take PrEP, and how you feel about PrEP. It should take about 30 minutes to complete the pre-screening survey.

**Additional costs and reimbursement**



You will not be paid and there will be no additional costs to you to participate in this pre-screening process.

This research project has been designed to make sure the researchers interpret the results fairly and appropriately. It has also been designed to keep study doctors or participants from jumping to conclusions.

# 4 Other relevant information about the research project

Participants in this pre-screening process will be adults who are HIV-negative and are at risk of getting HIV through sex. It is anticipated that between 300 and 600 people will complete this pre-screening process.

# 5 Do I have to take part in this pre-screening process?

Participation in any research project, including this pre-screening process, is voluntary. You do not have to be in this project if you do not want to. If you decide to pre-screen now, it is your right to change your mind later. You are free to withdraw from the pre-screening at any time. If you do decide to pre-screen, you will be given this Participant Information and Consent Form to sign and you will be given a copy to keep.

If you complete the screening process but either do not qualify for the study or decide not to start taking PrEP, you will be asked to complete an optional behavioural and lifestyle survey including questions about why you did not qualify/did not decide to start taking PrEP, so that we better understand how the screening and enrollment process works.

Your decision whether to take part or not to take part, or to take part and then withdraw, will not affect your routine treatment, your relationship with those treating you or your relationship with [insert Institution].

# 6 What are the alternatives to participation?

You do not have to be in the pre-screening process or the PRELUDE Study to receive treatment at this clinic. A separate PRELUDE Study information sheet and consent form reviews other alternatives and options to prevent HIV.

# 7 What are the possible benefits of taking part?

There is no guarantee or promise that you will receive any benefits from pre- screening for the PRELUDE Study. There is also no guarantee or promise that you will be enrolled in the PRELUDE study and receive PREP. However, people may like the idea that they are contributing to new knowledge about the prevention of HIV, and how people feel about PrEP and why they want to take PrEP.

# 8 What are the possible risks and disadvantages of taking part?

The questions we will ask you about your sexual behaviour may make you feel uneasy. However, you can stop answering the questions at any time and can change your mind about participating in this pre-screening at any time.

You may feel that some of the questions asked in the survey are stressful or upsetting. If you do not wish to answer a survey question, you may skip it and go to the next question, or you may stop immediately.

If you become upset or distressed as a result of your participation in the pre-screening, the research team will be able to arrange for counselling or other appropriate support. Any counselling or support will be provided by qualified staff who are not members of the research team. This counselling will be provided free of charge.

# 9 What if I withdraw from this pre-screening process?

If you decide not to complete the pre-screening process and have not yet completed the on-line survey, please let a member of the research team know.

If you do withdraw, you will be asked to complete and sign a 'Withdrawal of Consent' form; this will be provided to you by the research team.

If you do withdraw your consent from pre-screening, all the personal information that has already been collected for pre-screening will be kept so that the results of the research can be measured properly, and to comply with law. You should be aware that data collected up to the time you withdraw will form part of the research project results about pre-screening, but they will not contain any personal information about you, such as your name or address. If you do not want this to happen, you must tell the researcher before you pre-screen.

# Part 2 How is the pre-screening process being conducted?

# 10 What will happen to information about me?

**How information is used and stored**

By signing the consent form you consent to the research team collecting and using personal information about you for the pre-screening research project. Any information collected that can identify you will remain confidential. Your signed pre-screening consent form will be stored safety and kept confidential at the clinic.

Your de-identified answers to the HIV risk questions, along with your gender, age and sexual orientation will be shared with the researchers at the Kirby Institute. The Kirby researchers will not see your signed consent form and therefore will not know your identity.

Only the researchers at the Kirby Institute will have access to your internet-based survey information. This information will be stored in a password-protected database (SurveyGizmo), and will be treated as confidential and securely stored. All of your answers will be stored under your unique pre-screening code number, and the Kirby researchers will not know your identity. The Kirby researchers WILL be able to use your code number to link your survey answers with your HIV risk question answers. This will allow them to learn more about why people would like to take PrEP. The survey will be protected by passwords and University of New South Wales firewalls. **The researchers at the Kirby Institute will not share any of your survey answers with your clinic doctors, nurses or other staff.**

The paper based form with your HIV risk answers will be stored in a locked office, which only the members of the study team can access.

Your information will only be used for the purpose of this or other related research, and it will only be disclosed with your permission, except as required by law. Coded data may be used in future related research.

Any information obtained for the purpose of this pre-screening and for any future related research that can identify you will be treated as confidential and securely stored. It will be disclosed only with your permission, or as required by law.

**Publication and presentation of research findings**

The findings from the pre-screening part of the PRELUDE Study will be published and/or presented in a variety of places, most likely combined with everyone else's information. In any publication and/or presentation, information will be provided in such a way that you cannot be identified, except with your permission.

**Your rights to your information**

In accordance with relevant Australian and New South Wales privacy and other relevant laws, you have the right to request access to your information collected and stored by the research team. You also have the right to request that any information with which you disagree, be corrected. Please contact the study team member named at the end of this document if you would like to access your information.

**Information storage after the study**

After the end of the study, all study files associated with the PRELUDE Study will be archived in a locked storage facility and kept for at least 15 years after the end of the study. Coded data in electronic form will also be stored for this amount of time.

# 11 Complaints and compensation

If you suffer any distress or psychological injury as a result of this research project, you should contact the research team as soon as possible. You will be assisted with arranging appropriate treatment and support.

If you have any complaints about any aspect of pre-screening, the way it is being conducted or any questions about being a research participant in general, then you may contact the complaints person at the end of this information sheet.

# 12 Who is organising and funding the research?

The PRELUDE research project and this pre-screening is being conducted by the PRELUDE study team lead by the Chief Investigator, Dr Iryna Zablotska at the Kirby Institute, and is being funded by the NSW Ministry of Health. No financial benefits are expected for anyone as a result of this pre-screening process.

# 13 Who has reviewed the pre-screening procedure?

All research in Australia involving humans is reviewed by an independent group of people called a Human Research Ethics Committee (HREC). The ethical aspects of pre-screening and of the PRELUDE Study have been approved by the HREC of the St. Vincent's Hospital, Darlinghurst NSW. This project will be carried out according to the National Statement on Ethical Conduct in Human Research (2007). This statement has been developed to protect the interests of people who agree to participate in human research studies.

# 14 Further information and who to contact

The person you may need to contact will depend on the nature of your query. If you want any further information about this project, you can contact the study chief investigator Dr. Iryna Zablotska on 9385-0951 or any of the following people:

**Clinic contact person (for each study site to complete; may provide more than one if necessary)**

| | |
|---|---|
| Name | *[Name]* |
| Position | *[Position]* |
| Telephone | *[Phone number]* |
| Email | *[Email address]* |

For matters relating to research at the site at which you are participating, the details of the local site complaints person are:

**Complaints contact person (for each study site to complete)**

| | |
|---|---|
| Name | *[Name]* |
| Position | *[Position]* |
| Telephone | *[Phone number]* |
| Email | *[Email address]* |

If you have any complaints about any aspect of the project, the way it is being conducted or any questions about being a research participant in general, then you may contact:

**Reviewing HREC approving this research and HREC Executive Officer details**

| | |
|---|---|
| Reviewing HREC name | St. Vincent's Hospital Sydney HREC |
| HREC Executive Officer | Contact person - HREC Executive Officer |
| Telephone | 8382 2075 |
| Email | research@stvincents.com.au |

**Local HREC Office contact (Single Site -Research Governance Officer – for each study site to complete)**

| | |
|---|---|
| Name | *[Name]* |
| Position | *[Position]* |
| Telephone | *[Phone number]* |
| Email | *[Email address]* |

# Consent Form –PRELUDE Study Pre-screening

| | |
|---|---|
| **Title** | Pre-screening for: implementation of HIV preexposure prophylaxis with antiretroviral medications among people at high risk for HIV infection: A demonstration project |
| **Short Title** | The PRELUDE Study pre-screening |
| **Protocol Version** | 15 August 2014 |
| **Protocol Number** | HEPP 1403 |
| **Project Sponsor** | The Kirby Institute for Infection and Immunity in Society |
| **Coordinating Principal Investigator** | Dr. Iryna Zablotska, The Kirby Institute |
| **Associate Investigator(s)** | [insert if required by institution] |

## 1) Declaration by Participant

I have read the PRELUDE Study pre-screening Participant Information Sheet or someone has read it to me in a language that I understand.

I understand the purposes, procedures and risks of the pre-screening as described.

I have had an opportunity to ask questions and I am satisfied with the answers I have received.

I freely agree to participate in this pre-screening process as described and understand that I am free to withdraw at any time during the study without affecting my future health care.

I understand that I will be given a signed copy of this document to keep.

Name of Participant (please print) ______________________________

Signature ______________________ Date ______________________

Name of Witness* to
Participant's Signature (please print) ______________________________

Signature ______________________ Date ______________________

* Witness is not to be the investigator, a member of the study team or their delegate. In the event that an interpreter is used, the interpreter may not act as a witness to the consent process. Witness must be 18 years or older.

## Declaration by Study Doctor/Senior Researcher

I have given a verbal explanation of the pre-screening process, its procedures and risks and I believe that the participant has understood that explanation.

Name of Study Doctor/
Senior Researcher† (please print) ______________________________

Signature ______________________ Date ______________________

† A senior member of the research team must provide the explanation of, and information concerning, the research project.

Note: All parties signing the consent section must date their own signature

# Form for Withdrawal of Participation – PRELUDE Study Pre-screening

| | |
|---|---|
| **Title** | Pre-screening for implementation of HIV preexposure prophylaxis with antiretroviral medications among people at high risk for HIV infection: A demonstration project |
| **Short Title** | The PRELUDE Study pre-screening |
| **Protocol Version** | 15 August 2014 |
| **Protocol Number** | HEPP 1403 |
| **Project Sponsor** | The Kirby Institute for Infection and Immunity in Society |
| **Coordinating Principal Investigator** | Dr. Iryna Zablotska, The Kirby Institute |
| **Associate Investigator(s)** | [insert if required by institution] |

## Declaration by Participant

I wish to withdraw from participation in the above research project and understand that such withdrawal will not affect my routine treatment, my relationship with those treating me or my relationship with *[insert clinic/Institution]*.

Name of Participant (please print) ______________________________

Signature ______________________ Date ______________________

*In the event that the participant's decision to withdraw is communicated verbally, the Study Doctor/Senior Researcher will need to provide a description of the circumstances in the participant's source documentation.*

## Declaration by Study Doctor/Senior Researcher†

I have given a verbal explanation of the implications of withdrawal from the research project and I believe that the participant has understood that explanation.

Name of Study Doctor/
Senior Researcher† (please print) ______________________________

Signature ______________________ Date ______________________

† A senior member of the research team must provide the explanation of and information concerning withdrawal from the research project.

Note: All parties signing the consent section must date their own signature.

*Site: Insert Header with institution's name or institution's letterhead as required*

# Participant Information Sheet/Consent Form

**Interventional Study** - *Adult providing own consent*

*[Insert site name if required]*

| | |
|---|---|
| **Title** | Implementation of HIV preexposure prophylaxis with antiretroviral medications among people at high risk for HIV infection: A demonstration project |
| **Short Title** | The PRELUDE Study |
| **Protocol Version** | 30 September 2015 |
| **Protocol Number** | HEPP 1403 |
| **Project Sponsor** | The Kirby Institute for Infection and Immunity in Society |
| **Coordinating Principal Investigator** | Dr. Iryna Zablotska, The Kirby Institute |
| **Associate Investigator(s)** | *[Site: insert if required by institution]* |

## Part 1: What does my participation involve?

## 1 Introduction

You are invited to take part in a research project called PRELUDE because you are HIV-negative and may be at increased risk of getting HIV (Human Immunodeficiency Virus). HIV is the virus that causes Acquired Immunodeficiency Syndrome, or AIDS. In this research project, a medicine that has been used to treat people who *have* HIV is given to HIV-negative people to evaluate its use as the means to *lower* their chance of getting HIV. This is called preexposure prophylaxis or 'PrEP.' Prophylaxis means doing something to prevent an illness or infection.

This Participant Information Sheet/Consent Form (PICF) tells you about this research project. It explains the tests, treatments and the study requirements involved. Knowing what is involved will help you decide if you want to take part in the research.

Please read this information carefully. Ask questions about anything that you don't understand or want to know more about. Before deciding whether or not to take part, you might want to talk about it with a friend, partner, family member or your doctor.

Participation in this research is voluntary. If you don't wish to take part, you don't have to. You will receive the same quality of care whether or not you take part.

If you decide you want to take part in the PRELUDE research project, you will be asked to sign the consent section of this document. By signing it you will be telling us that you:

- Understand what you have read

- Consent to take part in the research project
- Consent to have the tests and treatments that are described
- Consent to the use of your personal and health information as described.

You will be given a copy of this Participant Information and Consent Form to keep.

# 2 What is the purpose of this research?

More and more people in Australia are getting HIV today, despite all prevention efforts, the availability of condoms and HIV testing. The NSW government would like to eliminate new HIV cases by the year 2020. This research will evaluate a new additional way to lower people's chances of getting HIV.

The drug being used in this project is called TRUVADA, and it is made by Gilead Sciences, Inc. TRUVADA is a single tablet made up of two different HIV medications: Viread (tenofovir disoproxil fumarate) and Emtriva (emtricitabine). Both these drugs have been widely used for many years to treat HIV. When used with other medicines in people who already ***have*** HIV, TRUVADA may help reduce the amount of HIV virus in the blood. It also may increase the number of cells in the blood that help fight off other infections. TRUVADA does not cure HIV or AIDS, and it is not an HIV vaccine.

In order for medicines to be used in Australia, they have to be approved by the Australian Federal Government. As a treatment for people who already have HIV, TRUVADA is approved for use in Australia, the United States, and other countries. As a medicine for PrEP, to lower chances of HIV in those who are not infected, TRUVADA has been approved in the US, but not in Australia. Therefore, in Australia, this study is considered experimental.

The purpose of the PRELUDE Study is to develop and evaluate a model of PrEP delivery in health services. It will see whether PrEP is an effective, safe and manageable option to help lower people's chances of getting HIV through sex. It will also study whether people in NSW find it easy and agreeable to take PrEP every day, why they take it, how it feels to take it, and whether it changes how they have sex. Finally, it will study how easy it is for doctors to use it with their patients, and what it costs the NSW government and clinics to provide PrEP.

This research study is being conducted by a group of investigators at the Kirby Institute, the University of New South Wales, and lead by Dr. Iryna Zablotska. It is funded by the NSW Ministry of Health. Gilead Sciences, who makes TRUVADA, is providing the study drug.

**How well does PrEP work?**

Research in other countries, including the U.S., has shown that the chances of getting HIV through sex is lowered for people who take PrEP ***<u>and</u>*** use condoms correctly during sex. How well PrEP works depends on how good people are at taking PrEP ***every day***.

Overall, studies in other countries have shown that taking PrEP lowers the risk of getting HIV by about 86 percent in homosexual, bisexual, and other men who have sex with men, and by about 70 percent in heterosexual adults. This means that some people on these research studies still got infected with HIV even though they were taking PrEP. However, when researchers looked specifically at people who were taking PrEP every day, they found that the risk of getting HIV was lowered even more. In one study of 2499 homosexual, bisexual and other men who have sex with men, those who took their PrEP pill every day had their risk of getting HIV lowered by 92 percent. In contrary, the risk of HIV infection was higher in people who did not take their tablets daily.

# 3 What does participation in this research involve?

Your involvement in the PRELUDE study will last up to about 36 months (3 years). This includes up to two weeks for screening, up to 30 months of taking PrEP (please note that the original period of up to 12 months is now extended to up to 30 months), and the completion of one follow-up visit and two follow-up questionnaires after you stop taking PrEP. The overall study will run for about five years.

By entering the study, you are not committing to being in the study and taking PrEP for the whole 30 months. For example, your participation in the study may end if you are no longer at risk of getting HIV through sex. You can also withdraw from the study at any time for your own reasons.

**Screening Visit**

The consent form must be signed by you before any study assessments are done.

First, doctors who are also researchers on this study will confirm that you are a good candidate for PrEP. . A number of tests will be conducted as a standard of care in New South Wales, Australia.

You will be tested for sexually transmitted infections, including HIV, chlamydia, gonorrhoea and syphilis. For gonorrhoea and chlamydia testing for men, this will be done by performing a swab of your rectum and collecting urine. Blood will be collected for syphilis, HIV testing and Hepatitis B testing.

For gonorrhoea and chlamydia testing for women, this will be done by performing [*sites to fill in method*: *a swab of your cervix, and a swab of your rectum if you have had anal sex in the last year AND/OR will be done by collecting urine]*. Blood will be collected for syphilis, HIV testing and Hepatitis B testing .

Your health and blood will be checked to make sure you are not at increased risk of possible side effects from TRUVADA, such as kidney or liver problems. You will also have a medical examination and will be asked about your medical history. If you are a woman who can get pregnant, you will have a pregnancy test before starting PrEP and while taking it, so that you and your doctor can together make an informed decision whether you start and continue taking PrEP.

Because both of the drugs in TRUVADA work against hepatitis B, you will be tested to know if you have active hepatitis B. This will allow doctors to recommend you an appropriate treatment. You may also be offered a vaccine against hepatitis B if the result of this test is negative. If the results show you were exposed earlier to hepatitis B and the infection has resolved, then you can be in the study. You may also be tested for Hepatitis C if this is considered standard at the clinic.

For all of these tests, [site to insert volume in millilitres and teaspoons or tablespoons, e.g. x tablespoons/y millilitres] of blood will be collected from a vein in your arm.

The samples collected for the standard of care tests will include: [site to insert accordingly.]

In order to qualify for the study, you must:

- Be at least 18 years old
- Have a negative HIV test within seven days of starting PrEP
- Not show possible signs of having HIV
- Be at high and ongoing risk for getting HIV
- Live in NSW, or visit NSW enough to attend all of your follow-up visits
- Be eligible for Medicare
- Be able to take TRUVADA once every day for at least 30 days
- Be able to attend clinic follow-up visits
- Be willing and able to have your blood collected at some of the follow-up visits as outlined below.
- Be willing to take a computer-based survey about your attitudes, behaviours and lifestyle at the beginning of the study, after each follow-up visit, and twice after you stop taking PrEP.
- Be willing and able to consent to this study.

You will not be allowed to be enrolled in this study if you have certain kidney or liver problems, if you have to take certain medications that might make PrEP unsafe, if you have known allergies to PrEP, or if you are breastfeeding. You also cannot be in this study if you have any conditions that would make it difficult for you to take PrEP every day, such as certain mental health issues, memory loss, difficulty thinking or some intellectual disabilities. If you will be in prison or if you plan to be away from NSW for a period of 3 months or longer during the study, you cannot be in this study because you will not be able to attend your follow-up visits.

In addition, the study doctor may ask you whether you have any symptoms consistent with a new HIV infection and you have experienced any known or suspected exposure to HIV during the last 30 days. If you have, you will receive additional HIV testing, and may be asked to delay starting PrEP for up to one month until the conclusive result of that testing. **It is very important for your safety during this study that you tell the doctor if you are experiencing any of the following at screening or before you start PrEP. It is also very important that you tell your doctor about any of these at your follow-up visits. If you do but it is not time for your regularly-scheduled follow-up visit, please contact your doctor or the study coordinator.**

- Fever
- Feeling tired (fatigue)
- Muscle aches (myalgia)
- Joint aches (arthralgia)
- Skin rash
- Headache
- Sore throat (pharyngitis)
- Night sweats
- Swelling of the lymph nodes around the head and neck (cervical adenopathy)

There may be a few days between the time you start screening for this study, and the time you can start taking PrEP medication. If you encounter a high-risk sexual event before you start taking TRUVADA, please tell your study doctor immediately, so that your study doctor can assess whether you need to receive medication for “Post-Exposure Prophylaxis,” or PEP before you start taking PrEP. This will not affect your ability to start taking PrEP once you finish with PEP if you are eligible.

When you first asked about this research, the clinic doctor collected some basic information about who is interested in PrEP and why (age, gender, sexual orientation, and answers to the HIV risk questions). You also completed an internet based survey on attitudes, behaviour and lifestyle. The pre-screening questionnaire did not contain any identifiable information. If you decide to be in this study, you will be asked to consent to allow that information to be linked with your PRELUDE study information. The study will also collect information about any PEP you may have taken right before starting PrEP.

**Starting PrEP**

After you have met all study criteria, you will be given a prescription for 30 days of TRUVADA. You will be asked to start taking TRUVADA as PrEP immediately. Your doctor will provide you with information to help you remember to take TRUVADA every day. You will also receive condoms, and counselling on condom use and safe sex.

It is important to know that:

- You will be expected to take TRUVADA every day, as per the instructions
- You will be encouraged to practice safe sex, including consistent and correct use of condoms
- You will have regular HIV and STI testing, at each quarterly follow-up visit and as needed between study visits.

**How do I take PrEP?**

A tablet of TRUVADA must be taken by mouth once a day, at about the same time every day, and it is recommended to be taken with food. For this study, you will be asked to take PrEP daily for at least 30 days. You may continue to take PrEP every day for up to 30 months total (please note that the original period on PrEP up to 12 months is now extended to up to 30 months).

If you miss a dose of TRUVADA, your doctor may advise you to take the missed pill as soon as you remember on that same day, but to not take more than one dose of TRUVADA in a 24 hour period. Your doctor may also advise that if you miss a dose, and it is almost time for your next dose, to wait and take the next dose at your regular time. Contact your medical provider as soon as possible if you take more than one pill of TRUVADA a day, to avoid overdosing.

TRUVADA should be stored at room temperature in its original container. The container should be kept tightly closed and out of the reach of children. Do not give TRUVADA prescribed to you to other people.

**Follow-up Visits while taking PrEP**

After you start the study, you will attend a follow-up visit at the clinic one month after you start taking PrEP, and then every three months after you start taking PrEP, for up to 30 months (that is the original period of taking PrEP up to 12 months is now extended to up to 30 months). At each of these visits, you will have an HIV test. It is very important that you take these HIV tests because TRUVADA cannot be taken alone by people who are HIV-positive (see HIV Infection section below).

At each follow-up visit you will be asked about your health, how well you remembered to take TRUVADA, and your sexual practices including safer sex practices. Each study visit will be about 15 minutes longer than your regular clinic visits.

At each of the quarterly visits (every three months), you will again be tested for HIV and sexually transmitted infections, and will be tested for your kidney and liver health. A total of about [site to insert volume in millilitres and teaspoons or tablespoons, e.g. x tablespoons/y millilitres] of blood will be collected from a vein in your arm for these tests. If you are a woman who can get pregnant, you will also be given a pregnancy test. You will be reminded about how to take PrEP, and will be given safe sex information, services, or referral for any services you might need.

You may also have an additional 25 millilitres (ml) or 1 and a half tablespoons of blood collected at some of the follow-up visits,, to measure the amount of TRUVADA that is present in your blood. You will have no more than three collections of this blood during the study.
If you are still eligible to continue taking PrEP, you will be given a new prescription for TRUVADA at these visits.

All of these follow-up procedures and discussions are considered standard care for people who are at risk for HIV infection and/or are taking anti-HIV medications. This means that even if PrEP wasn't considered research, these things would be done for your health and safety.

Within two and no more than within seven days of each clinic visit while you are taking PrEP, you will complete a self-administered, web-based, online questionnaire. The questionnaire will take approximately 30 minutes to complete. If you do not have a home computer that you are able to use, then you will be provided an alternate means of completing the questionnaire. The survey does not work well on mobile devices, so should be completed on a desktop or laptop computer. The questions will ask about things like your relationships, sexual practices and other related behaviour (in the preceding three months or since your last online questionnaire), your beliefs and attitudes about HIV and its prevention, and your experience with taking PrEP. It will also ask about how often you have taken your study medications since your previous online questionnaire. This includes information on when you have taken or missed doses of PrEP and when you have had and had not use condoms. The more accurate the information, the more useful it will be for the research and future use of PrEP.

The answers to the online questionnaire go directly to the researchers at the Kirby Institute. They will not share any of your survey answers with your clinic, doctors, nurses or other staff. The study researchers are using the answers from these surveys to see how PrEP changes peoples' lives in general, not at an individual level.

**Stopping PrEP**

It is your right as a research participant to decide to stop taking PrEP at any time. During the 30-month period, you may choose to stop the PrEP medication if you feel that you no longer need it. Your study doctor may also have you stop the PrEP medication if she or he feels it is not needed, or it is not safe for you stay on PrEP. For example, you will be stopped from the study if you become HIV positive, if you start to experience kidney, liver or other unacceptable health problems, or if you need medication that interacts with TRUVADA.

It is strongly recommended for your safety that you take PrEP for at least 30 days at a time. Your doctor will discuss with you how to plan to stop PrEP during the study period if you need to. This may also involve taking TRUVADA as “Post-Exposure Prophylaxis,” or PEP, if you and your doctor find out that you might have been exposed to HIV. If at any time during the study you stop taking PrEP and start taking PEP, the results of your HIV, STI and other tests taken because you are on PEP will be collected for the study as well. If you become HIV positive during this study, the results of any additional tests about your HIV infection, such as tests of resistance to different HIV treatments, will be collected for this study.

When you stop taking PrEP at any time during the study (including at 30 months), you will attend an end-of-study visit. At this visit, all quarterly follow-up visit procedures will be conducted.

If you change your mind about stopping TRUVADA and want to start taking it again, you can re-enter the study within the six months after you stopped, as long as you still qualify and the study is still open for re-enrolment. The option to re-enter will not be available during the last four months of the study or if the study has been filled.

**Follow-up Visits after you stop taking PrEP**

Three and six months after you stop taking PrEP, you will complete the on-line questionnaires for the researchers at Kirby, as already described. You will also be asked if the researchers can have access to your information from the NSW HIV Registry up to six months after you stop taking PrEP. That will allow the researchers to see if you test positive for HIV after you stop taking PrEP.

**Additional costs and reimbursement**

You will not be paid and there will be no additional costs to you to be in this project. All tests and medical care required as part of the research project will be provided to you as it normally would be through Medicare. During the 30 month period, the medication will be provided free-of-charge by Gilead Sciences. Because this study wants to know what it will be like to use PrEP in the real-world, you will not be reimbursed for your travel to the clinics, clinic parking, your time, or other expenses associated with the research project.

This research project has been designed to make sure the researchers interpret the results fairly and appropriately. It has also been designed to keep study doctors or participants from jumping to conclusions.

# 4 What do I have to do?

Participation in this study does not require any changes to your diet or to your participation in sports. It is important let the study doctor know any other medications you are already taking or start taking while on TRUVADA.

Even if this was not a research study, all people who take PrEP will need to do other things to prevent getting infected with HIV and other sexually transmitted infections.

People who take PrEP must:

- **Take PrEP every day**. Not taking PrEP every day can increase your risk of getting HIV. Because this is so important, your doctor will talk with you about the best ways to help you remember to take your pill.
- **Continue using condoms every time when having sex:** PrEP is not 100 percent effective in preventing HIV, and does not protect against getting other sexually transmitted infections, like herpes, gonorrhoea, and syphilis.
- **Let the study doctor know if they have not been taking PrEP every day and may have been exposed to HIV:** When taken every day, PrEP can still take at least seven days to build up the maximum protection. If you think you have been exposed to HIV, your doctor will discuss your risk with you, and whether you may need Post-Exposure Prophylaxis. Do not wait until your next follow-up visit to discuss this with your doctor. It is important to start Post-Exposure Prophylaxis within 72 hours after you were exposed to HIV.

It is also important to know that:

- Taking extra pills will not provide you with extra protection.
- PrEP is not a "morning after" pill; it will not work if only taken right before or right after exposure to HIV.
- If you decide to stop taking PrEP for any reason, you should make sure you have taken PrEP every day for four weeks after the last time you may have had sex that put you at risk of HIV.
- If you have been exposed to HIV, your doctor may recommend to stop PrEP and take a course of Post-Exposure Prophylaxis.

# 5 Other relevant information about the research project

Participants in this study will be adults who are HIV-negative and are at risk of getting HIV through sex. The study allows "up to 300 person-years" of PrEP. This means that if everyone who participates takes one year of TRUVADA, there will be 300 people in the study. However, there may be more people in the study if at least some participants take TRUVADA for less than one year.

Everyone in the study will take TRUVADA for PrEP, and everyone will have the same study visits. The PRELUDE Study is open to all adults regardless of sex, gender or sexual preference. There will be between four and 12 sites involved in the project. The coordinating principal investigator for this project is Dr Iryna Zablotska. The PRELUDE Study Management Team is responsible for the day-to-day management and coordination of the study. Each clinic in the study also has investigators who are part of the study.

# 6 Do I have to take part in this research project?

Participation in any research project is voluntary. You do not have to be in this project if you do not want to. If you decide to be in this project now, it is your right to change your mind later. You are free to withdraw from the project at any time. If you do decide to take part, you will be given this Participant Information and Consent Form to sign and you will be given a copy to keep.

Your decision whether to take part or not to take part, or to take part and then withdraw, will not affect your routine treatment, your relationship with those treating you or your relationship with [insert Institution].

# 7 What are the alternatives to participation?

You do not have to be in this research project to receive treatment at this clinic. Other options to prevent HIV include: advice and counselling on safe sex practices including the use of condoms, and regular HIV and STI testing. HIV post-exposure prophylaxis (PEP) is available at your clinic after any sexual or injecting exposures that may have put you at risk of getting HIV. Your study doctor will discuss these options with you before you decide whether or not to take part in this research project. You can also discuss these options with your local doctor.

Other anti-HIV medications are currently being studied for use as PrEP. However, no other medications have been approved for the prevention of HIV.

# 8 What are the possible benefits of taking part?

There is no guarantee or promise that you will receive any benefits from being in this research project. Research shows that taking TRUVADA every day, combined with safer sex practices including using condoms during sex, may decrease your risk of getting HIV. Some participants may experience direct health benefits due to testing for HIV and STIs more regularly than they would normally otherwise. Participants may also like the idea that they are contributing to new knowledge about the prevention of HIV.

# 9 What are the possible risks and disadvantages of taking part?

Medications often cause side effects. You may have none, some, or all of the effects known to be associated with the use of TRUVADA and listed below. They may be mild, moderate or severe. If you have any of these side effects, or are worried about them, talk with your study doctor. Your study doctor will also be looking out for side effects.

There may be side effects that the researchers do not expect or do not know about which may be serious. Tell your study doctor immediately about any new or unusual symptoms that you experience.

Many side effects go away shortly after treatment ends. However, sometimes side effects can be serious, long lasting or permanent. If a severe side effect or reaction occurs, your study doctor may need to stop your treatment. Your study doctor will discuss the best way of managing any side effects with you.

**Possible Side Effects of TRUVADA**

TRUVADA has not been used in HIV negative people as much as it has been used in people who are HIV positive. In people who are HIV negative, it has only been used by several thousands of participants in clinical trials of PrEP and by HIV negative people taking it as part of Post-Exposure Prophylaxis or PEP. The dose of TRUVADA used in PEP and PrEP is the same, and in both cases it is taken as a daily tablet. People who take TRUVADA for PrEP and PEP usually take TRUVADA for shorter periods of time than those who take TRUVADA for HIV treatment. As well, people who take TRUVADA for HIV treatment most often take more than one HIV medication at the same time.

**In people who did not have HIV and took TRUVADA for PrEP:** In previous research studies, between three and nine percent of such participants reported the following four side effects, and these levels were higher than among the participants who did not take TRUVADA (took a “sugar pill”):

- headache
- stomach pain
- involuntary weight loss
- nausea or upset stomach

In PrEP studies where these side effects were reported, these were modest, usually only occurred in the first one or two months of taking the drug and did not last.

**In people who *have* HIV who took TRUVADA:** the most common (reported in 10 percent and greater) side effects according to the company that makes TRUVADA are:

- diarrhoea
- nausea/upset stomach
- tiredness
- headache
- dizziness
- depression
- difficulty sleeping
- strange dreams
- rash

**Possible allergic reaction:** In clinical trials of TRUVADA, a small number of people have had an allergic reaction to tenofovir, one of the drugs in TRUVADA. Symptoms of an allergic reaction may include fever, rash, upset stomach, vomiting, loose or watery stools, stomach pain, achiness, shortness of breath, a general feeling of illness or a potentially serious swelling of the face, lips, and/or tongue.

The company that makes TRUVADA also warns that the following changes in laboratory tests are possible. This information is based mostly on experience with people who take TRUVADA for HIV treatment, and on other drugs that are like TRUVADA. Because HIV by itself can also cause these same problems, it is difficult to know how much TRUVADA alone contributes to kidney and bone disease:

- kidney problems
- decreases in the minerals in their bones
- build-up of lactic acid in the blood
- enlarged liver

Because the last two of these side effects are so serious, the symptoms of each are listed below. **Call your doctor immediately if you get these symptoms:**

| Symptoms of too much lactic acid in the blood | Symptoms of severe liver problems |
|---|---|
| • weakness or being more tired than usual<br>• unusual muscle pain<br>• being short of breath or fast breathing<br>• nausea, vomiting, and stomach-area pain<br>• cold or blue hands and feet<br>• feel dizzy or lightheaded<br>• fast or abnormal heartbeats | • your skin or the white part of your eyes turns yellow<br>• dark “tea-colored” urine<br>• light-colored stools<br>• loss of appetite for several days or longer<br>• nausea<br>• stomach-area pain |

**To manage kidney and liver health:** Your doctor will be taking blood tests to check you kidneys and liver before you start and while you are taking TRUVADA. Your study doctor may tell you to stop taking TRUVADA if you develop kidney or liver problems during the study.

**Bone health:**

People who have HIV infection have usually taken anti-HIV treatment for long periods of time and have taken more than one HIV medication including the type of drugs that are in TRUVADA. They have experienced a three- to four-percent decline in the minerals in their bones.

In HIV negative people on TRUVADA bone scans were taken only in two other PrEP research studies – one using TRUVADA and one using one out of the two drugs that make up TRUVADA. These scans found that less than one percent of study participants tested experienced a small decrease in the minerals in their bones during the first few months of PrEP. This decline either stopped getting worse or

returned to normal after the first few months. No increase in bone breaks was observed. Because of this, bone scans are not recommended for HIV negative people in this study. However, if you have a history of bone fractures/breaks or if you are at significant risk for osteoporosis, please tell the study doctor, who will help you manage this risk.

**HIV infection**

People with HIV need full HIV medication and treatment. You will have an HIV test at every follow-up visit. If you become infected with HIV, your study doctor will have you stop taking PrEP immediately. This is because taking TRUVADA by itself when you have HIV can make the virus resistant to some HIV drugs so that they may no longer work for you and limit your HIV treatment options over time.

**Hepatitis B infection**

If you become infected with Hepatitis B while you are taking TRUVADA, please tell your study doctor immediately, and do not stop taking TRUVADA on your own. If you stop TRUVADA while you have Hepatitis B, your hepatitis symptoms may get worse. You study doctor will help you stop taking TRUVADA and will monitor you for safety.

**Pregnancy and breastfeeding**

The effects of TRUVADA on an unborn child and on a newborn baby are not known. You may not participate in this study if you are breast-feeding at the start of the study. If you are female and you can possibly get pregnant, you must also take a pregnancy test at every follow-up visit. All participants are expected to continue use of safer sex practices [non-Catholic sites may add: including the use of condoms].

Any participants who are taking PrEP in conjunction with conception must take daily doses of TRUVADA beginning one month before a conception attempt. TRUVADA must continue to be taken daily until one month after the last attempt to conceive.

Women who become pregnant while in this study should make an informed decision about whether or not to keep taking PrEP while pregnant. If you become pregnant during this study, you should tell your study doctor immediately. Your study doctor will discuss with you your risks of HIV infection during pregnancy, the possible risks of TRUVADA on your pregnancy and child, and whether or not you should stay in the study.

If you become pregnant, we will ask for copies of or access to the parts of your medical records that discuss your pregnancy, delivery, and your infant's health.

**Other risks**

Having a blood sample taken may cause some discomfort, pain or bruising. Sometimes, the blood vessel may swell, or blood may clot in the blood vessel, or the spot from which blood is taken could become inflamed. Some people may feel dizzy when having blood taken, and may occasionally faint. Rarely, there could be a minor infection or bleeding. If this happens, it can be easily treated.

You may experience pain or discomfort from the rectal or cervical swab. In some cases, you may have some bleeding.

The questions we will ask you about your sexual behaviour may make you feel uneasy. However, you do not have to answer any question that you do not want to and you can stop answering the questions at any time.

Although your information will be kept confidential by study staff, it is possible that your friends, family or people in your community may find out that you are in this study. If that happens, you may experience stigma as a result of being involved in a study about HIV. If this or any other issue may make you become upset or distressed, the study doctor will be able to arrange for counselling or other appropriate support

Any counselling or support will be provided by qualified staff who are not members of the research project team. This counselling will be provided free of charge.

## 10 What will happen to my samples?

Your blood will be collected at the beginning of the study and at follow-up visits, to check your kidney and liver health, and to test for HIV, STI and hepatitis B infection. This is considered routine care for people who take TRUVADA for PrEP. The study team will know that these samples belong to you as they will be sent to the lab with your personal information, as they normally are for routine care.

If a test shows you have HIV or Hepatitis, you will have follow-up counselling and appropriate medical advice. If your test results are positive, the study doctors are required by law to notify government health authorities. Signing the consent form means that you agree to have this testing; it will not be done without your consent.

As mentioned before, 25 millilitres (ml) blood may be collected at some of the follow-up visits, to measure the amount of TRUVADA in your blood. This will tell the study researchers about how study participants took TRUVADA according to the instructions. Your doctor will not be given the results of your drug level blood tests because the study researchers are using this test to examine the use of PrEP in the study group as a whole, not to examine how often an individual uses the PrEP medication. Analyses of the cumulated samples will be conducted at the midpoint and/or the end of the study period.

For these tests, your study ID number, date of birth, and a 2 x 2 name code (first two letters of your first name – first two letters of your last name) will be placed on your sample tubes and the form the site uses to document your blood draw. The ID number can be linked back to you by using a decoding key, which will be stored separately and only known to an authorized member/s of the research study team. The study researchers will be able to link your sample results to your other data by using your study ID number. These samples of your blood will be transferred twice. First, they will be sent to the St Vincent's Centre for Applied Medical Research laboratory for processing. This laboratory will be able to see your ID number, your date of birth and your name code (2 x 2 code comprising first two letters of participant's first name – first two letters of subject's surname). This laboratory must comply with all confidentiality requirements. The samples will then be sent to the Clinical Pharmacology Analytical Lab (CPAL) at the Johns Hopkins University School of Medicine, Baltimore Maryland, USA, for analysis of the level of TRUVADA present. This laboratory will be able to see only your study ID number, but not your date of birth or name code.

You will be asked to provide additional consent for the collection and storage of your blood for banking purposes.

## 11 What if new information arises during this research project?

Sometimes during a research project, new information becomes available about the medication that is being studied. If this happens, your study doctor will tell you about it and will discuss with you whether you want to continue. If you decide to withdraw, your study doctor will make arrangements for your regular health care to continue. If you decide to continue in the research project you may be asked to sign an updated consent form that has the new information in it.

Also, on receiving new information, your study doctor might consider it to be in your best interest to withdraw you from the research project. If this happens, he/ she will explain the reasons and arrange for your regular health care to continue.

## 12 Can I have other treatments during this research project?

It is not anticipated that TRUVADA will affect any other medications you may be taking. However, it is important to let the study doctor know about any other medications or treatments you may be taking and/or using, including over-the-counter medications, vitamins or herbal remedies, acupuncture or other alternative treatments. You should also tell your study doctor about any changes to these during your time in the research project. Your study doctor will explain to you which treatments or medications cannot be used if you are involved in the PRELUDE study.

## 13 What if I withdraw from this research project?

If you decide to withdraw from this project, please let a member of the research team know before you do so. This will allow you to find out about any possible health risks or special requirements before you stop.

If you do withdraw your consent during this research project, the study team will ask you to continue to complete the attitude, behavioural and lifestyle questionnaires online. All the personal information that they have already collected for this study will be kept so that the results of the research can be measured properly, and to comply with law. You should be aware that data collected up to the time you withdraw will form part of the research project results. If you do not want this to happen, you must tell the researcher before you join the PRELUDE study.

If you choose to stop taking PrEP, you can still agree to complete the discontinuation and 3 months post-discontinuation follow-up visits, to complete discontinuation and post-discontinuation online surveys, and/or to allow the team to check your HIV status six months after you discontinue. If you agree to any of these activities after you stop PrEP, please do not complete the revocation of consent form until these follow-up activities are completed.

## 14 Could this research project be stopped unexpectedly?

This research project may be stopped unexpectedly for reasons which may include:

- Unacceptable side effects
- PrEP being shown not to be effective
- Decisions by local regulatory/health authorities.

## 15 What happens when the research project ends?

PrEP will be available to you for up till the end of the study. At the present time TRUVADA is not registered for use in Australia. At the end of this study Gilead Sciences may not agree to continue to supply TRUVADA, or may agree to provide an ongoing supply but only under certain conditions and for a limited time period. At the end of the study, if the study drug is still not registered in Australia AND subsidised on the PBS for your condition, then [*insert study site name*] will not be able to fund ongoing supplies of the drug. TRUVADA may never be registered in Australia for PrEP. This means that TRUVADA may not be available or may be expensive for you to buy. Your study doctor will discuss with you other options to help prevent HIV infection.

We estimate that all participants in the study will have completed the study by the end of 2018. After this time, the data will be analysed and a one-page summary of results will be prepared for all participants. Study staff will send copies of the summary to each participating clinic and you will be given the summary at your next routine clinic visit with your doctor.

# Part 2 How is the research project being conducted?

# 16 What will happen to information about me?

**How information is used and stored**

By signing the consent form you agree to let the study doctor and related research staff collect and use personal information about you for the research project. Any information collected for this research project that can identify you will remain confidential.

Information collected for this research will be kept in two different databases:

***Health information:*** Health information collected about you for this research will be entered into and stored in a study-specific password-protected database. This information will be coded, which means that your name won't be included. However, the study staff can link this information back to the rest of your personal information, such as your clinic medical records or Commonwealth and State agency health and disease-related registries, by using a decoding key containing your date of birth. Your research health information may also be recorded in your health records at the clinic. If at any time during the study your study doctor requests additional HIV testing beyond the standard screening test, copies of these additional test results will be collected so that the researchers can better understand the HIV testing processes involved with PrEP management. Your name and all identifying information will be blocked out and your study ID number will be written on any photocopies collected by the researcher.

***Behavioural survey information:*** Only the researchers at the Kirby Institute will have access to your internet-based survey information. This information will be stored in a password-protected database (SurveyGizmo), and will be treated as confidential and securely stored. All participants will be allocated a study number, and all electronic data will be stored under this code, so your name and identifying information will not be stored with the information collected about you during the study. The behavioural survey system will store your preferred email address so that the system can send you invitations to compete the surveys, and automatic reminders if you do not complete a survey within the prefered time frame. Your email address is stored separately from your clinical information. Your name is not stored in any database.

All electronic databases kept at the Kirby Institute will be protected by passwords and University of New South Wales firewalls. Identifying details (such as your name and contact details) and the fact that you are participating in this study will be known only to: your doctor; the research nurse/s at your clinic; and the project leader and research assistant based at the Kirby Institute, UNSW, Sydney. The project leader and research assistant at the Kirby Institute will need your name and contact details so as to assist with follow-up and provide you with information about the study as it progresses.

The researchers at the Kirby Institute will not share any of your survey answers with your clinic doctors, nurses or other staff. However, if you do not complete a behavioural survey within the preferred time frame nor after you have received two electronic reminders, the Kirby Institute researchers may ask the study staff to contact you to provide another reminder.

Any paper based forms used for this study will be stored in a locked office, which only the members of the study team can access.

Your information will only be used for the purpose of this or other related research, and it will only be disclosed with your permission, except as required by law. Coded data may be used in future related research.

Information about you may be obtained from your health records held at this and other health services for the purpose of this research. By signing the consent form you agree to the study team accessing health records if they are relevant to your participation in this research project.

Any information obtained for the purpose of this research project and for any future related research described in Part 16 that can identify you will be treated as confidential and securely stored. It will be disclosed only with your permission, or as required by law.

**Inspection of research records**

It is possible that your health research study records may be inspected in order to make sure that the study is being conducted appropriately and safely, and that the information is being reported truthfully. Possible inspectors include: representatives from the Kirby Institute, relevant authorities and authorised representatives of the Kirby Institute, the institution relevant to this Participant Information Sheet, *[Name of institution]*, or as required by law. By signing the Consent Form, you authorise release of, or access to, this confidential information to these relevant study personnel and regulatory authorities.

**Publication and presentation of research findings**

The findings from this research project will be published and/or presented in a variety of places, most likely combined with everyone else's information. In any publication and/or presentation, information will be provided in such a way that you cannot be identified, except with your permission.

**Your rights to your information**

In accordance with relevant Australian and New South Wales privacy and other relevant laws, you have the right to request access to your information collected and stored by the research team. You also have the right to request that any information with which you disagree, be corrected. Please contact the study team member named at the end of this document if you would like to access your information.

**Information storage after the study**

After the end of the study, your study files will be archived in a locked storage facility and kept for at least 15 years after the end of the study. Coded data in electronic form will also be stored for this amount of time.

# 17 Complaints and compensation

If you suffer any injuries or have complications as a result of this research project, you should contact the study team as soon as possible and you will be assisted with arranging appropriate medical treatment.

You may have a right to take legal action to obtain compensation for any injuries or complications resulting from your participation in the study. Compensation from the sponsor of this study, the University of New South Wales, may be available if your injury or complication is caused by the drugs or procedures, or by the negligence of any of the parties conducting the study. If you receive compensation that includes an amount for medical expenses, you will be required to pay for your medical treatment from those compensation monies.

If you are not eligible for compensation for your injury or complication under the law, but are eligible for Medicare, you can receive any medical treatment required to treat the injury or complication, free of charge, as a public patient in any Australian public hospital.

The people running this study agree to follow the Medicines Australia *Guidelines for Compensation for Injury Resulting from Participation in an Industry-Sponsored Clinical Trial*. These Guidelines allow for some claims for compensation to be settled without the need for legal action to be taken. The fact that the people running this study have agreed to abide by these guidelines in respect of the clinical trial does not affect your rights to pursue legal action in respect of any injury you may suffer as a result of participation. You can obtain a copy of these Guidelines from the Secretary of the Human Research Ethics Committee."

# 18 Who is organising and funding the research?

This research project is being conducted by the Chief Investigator, Dr Iryna Zablotska at the Kirby Institute, and is being funded by the NSW Ministry of Health. The manufacturer of TRUVADA, Gilead Sciences, is providing the study drug.

[*Insert site name*] will receive payments from the NSW Ministry of Health for time spent undertaking this research project. No member of the research team will receive personal financial benefit from your involvement in this research project (other than their ordinary wages).

Gilead Sciences may benefit financially from this research project if, for example, the project assists Gilead to obtain approval for TRUVADA to be used as PrEP in Australia.

In addition, if knowledge acquired through this research leads to discoveries that are of commercial value to Gilead Sciences, the study doctors or their institutions, there will be no financial benefit to you or your family from these discoveries.

# 19 Who has reviewed the research project?

All research in Australia involving humans is reviewed by an independent group of people called a Human Research Ethics Committee (HREC). The ethical aspects of this research project have been approved by the HREC of the St. Vincent's Hospital, Darlinghurst NSW. This project will be carried out according to the National Statement on Ethical Conduct in Human Research (2007). This statement has been developed to protect the interests of people who agree to participate in human research studies.

# 20 Further information and who to contact

The person you may need to contact will depend on the nature of your query. If you want any further information about this project or if you have any medical problems which may be related to your involvement in the project (for example, any side effects), you can contact the study chief investigator Dr. Iryna Zablotska on 9385-0951 or any of the following people:

**Clinical contact person (for each study site to complete; may provide more than one if necessary)**

| | |
|---|---|
| Name | *[Name]* |
| Position | *[Position]* |
| Telephone | *[Phone number]* |
| Email | *[Email address]* |

For matters relating to research at the site at which you are participating, the details of the local site complaints person are:

**Complaints contact person (for each study site to complete)**

| | |
|---|---|
| Name | *[Name]* |
| Position | *[Position]* |
| Telephone | *[Phone number]* |
| Email | *[Email address]* |

If you have any complaints about any aspect of the project, the way it is being conducted or any questions about being a research participant in general, then you may contact:

## Reviewing HREC approving this research and HREC Executive Officer details

| | |
|---|---|
| Reviewing HREC name | St. Vincent's Hospital Sydney HREC |
| HREC Reference number | HREC/14/SVH/130 |
| HREC Executive Officer | Contact person - HREC Executive Officer |
| Telephone | 8382 2075 |
| Email | research@stvincents.com.au |

## Local HREC Office contact (Single Site -Research Governance Officer – for each study site to complete)

| | |
|---|---|
| Name | *[Name]* |
| Position | *[Position]* |
| Telephone | *[Phone number]* |
| Email | *[Email address]* |

**Consent Form**

| | |
|---|---|
| **Title** | Implementation of HIV preexposure prophylaxis with antiretroviral medications among people at high risk for HIV infection: A demonstration project |
| **Short Title** | The PRELUDE Study |
| **Protocol Version** | 30 September 2015 |
| **Protocol Number** | HEPP 1403 |
| **Project Sponsor** | The Kirby Institute for Infection and Immunity in Society |
| **Coordinating Principal Investigator** | Dr. Iryna Zablotska, The Kirby Institute |
| **Associate Investigator(s)** | [insert if required by institution] |

**Declaration by Participant: Consent to be in the PRELUDE Study**

I have read the Participant Information Sheet or someone has read it to me in a language that I understand.

I understand the purposes, procedures and risks of the research described in the project.

I give permission for my doctors, other health professionals, hospitals or laboratories outside this hospital to release information to *[Insert site name here]* concerning my disease and treatment for the purposes of this project. I understand that such information will remain confidential.

I give permission to link my details using a unique identifying code with the Commonwealth and State agency health and disease-related registries. Such registries include, but are not limited to, registries of HIV, STIs and use of HIV post-exposure prophylaxis. This information will allow more accurate ascertainment of HIV and STI diagnoses, as well as the use of the antiretroviral medications outside of the demonstration project PRELUDE.

I have had an opportunity to ask questions and I am satisfied with the answers I have received.

I freely agree to participate in this research project as described and understand that I am free to withdraw at any time during the study without affecting my future health care.

I understand that I will be given a signed copy of this document to keep.

I understand that if I decide to discontinue the study treatment, I may be asked to attend follow-up visits to allow collection of information regarding my health status. Alternatively, the investigator/sponsor will request my permission to access my medical records for collection of follow-up information for research and analysis.

Please ***initial*** one option for the following (yes or no):

I agree that the Kirby Institute may contact me about future research projects that I may qualify for.

(initial one option) __________Yes __________No

Name of Participant (please print) ____________________

Signature ____________________ Date ____________________

| |
|---|
| Name of Witness* to<br>Participant's Signature (please print) ______________________________<br><br>Signature ________________________ Date ________________________ |

* Witness is not to be the investigator, a member of the study team or their delegate. In the event that an interpreter is used, the interpreter may not act as a witness to the consent process. Witness must be 18 years or older.

## Declaration by Study Doctor/Senior Researcher

I have given a verbal explanation of the research project, its procedures and risks and I believe that the participant has understood that explanation.

| |
|---|
| Name of Study Doctor/<br>Senior Researcher† (please print) ______________________________<br><br>Signature ________________________ Date ________________________ |

† A senior member of the research team must provide the explanation of, and information concerning, the research project.

Note: All parties signing the consent section must date their own signature.

# Form for Withdrawal of Participation

| | |
|---|---|
| **Title** | Implementation of HIV preexposure prophylaxis with antiretroviral medications among people at high risk for HIV infection: A demonstration project |
| **Short Title** | The PRELUDE Study |
| **Protocol Version** | 30 September 2015 |
| **Protocol Number** | HEPP 1403 |
| **Project Sponsor** | The Kirby Institute for Infection and Immunity in Society |
| **Coordinating Principal Investigator** | Dr. Iryna Zablotska, The Kirby Institute |
| **Associate Investigator(s)** | [insert if required by institution] |

## Declaration by Participant

I wish to withdraw from participation in the above research project and understand that such withdrawal will not affect my routine treatment, my relationship with those treating me or my relationship with *[insert clinic/Institution]*.

Name of Participant (please print) ______________________________

Signature ______________________ Date ______________________

*In the event that the participant's decision to withdraw is communicated verbally, the Study Doctor/Senior Researcher will need to provide a description of the circumstances in the participant's source documentation.*

## Declaration by Study Doctor/Senior Researcher†

I have given a verbal explanation of the implications of withdrawal from the research project and I believe that the participant has understood that explanation.

Name of Study Doctor/
Senior Researcher† (please print) ______________________________

Signature ______________________ Date ______________________

† A senior member of the research team must provide the explanation of and information concerning withdrawal from the research project.

Note: All parties signing the consent section must date their own signature.

*Site: Insert Header with institution's name or institution's letterhead as required*

# Participant Information Sheet/Consent Form

**Interventional Study** - *Adult providing own consent*

*[Insert site name if required]*

| | |
|---|---|
| **Title** | Implementation of HIV preexposure prophylaxis with antiretroviral medications among people at high risk for HIV infection: A demonstration project |
| **Short Title** | The PRELUDE Study |
| **Protocol Version** | 30 September 2015 |
| **Protocol Number** | HEPP 1403 |
| **Project Sponsor** | The Kirby Institute for Infection and Immunity in Society |
| **Coordinating Principal Investigator** | Dr. Iryna Zablotska, The Kirby Institute |
| **Associate Investigator(s)** | *[Site: insert if required by institution]* |

## Part 1: What does my participation involve?

### 1 Introduction

You are invited to take part in a research project called PRELUDE because you are HIV-negative and may be at increased risk of getting HIV (Human Immunodeficiency Virus). HIV is the virus that causes Acquired Immunodeficiency Syndrome, or AIDS. In this research project, a medicine that has been used to treat people who *have* HIV is given to HIV-negative people to evaluate its use as the means to *lower* their chance of getting HIV. This is called preexposure prophylaxis or 'PrEP.' Prophylaxis means doing something to prevent an illness or infection.

This Participant Information Sheet/Consent Form (PICF) tells you about this research project. It explains the tests, treatments and the study requirements involved. Knowing what is involved will help you decide if you want to take part in the research.

Please read this information carefully. Ask questions about anything that you don't understand or want to know more about. Before deciding whether or not to take part, you might want to talk about it with a friend, partner, family member or your doctor.

Participation in this research is voluntary. If you don't wish to take part, you don't have to. You will receive the same quality of care whether or not you take part.

If you decide you want to take part in the PRELUDE research project, you will be asked to sign the consent section of this document. By signing it you will be telling us that you:

- Understand what you have read

- Consent to take part in the research project
- Consent to have the tests and treatments that are described
- Consent to the use of your personal and health information as described.

You will be given a copy of this Participant Information and Consent Form to keep

# 2 What is the purpose of this research?

More and more people in Australia are getting HIV today, despite all prevention efforts, the availability of condoms and HIV testing. The NSW government would like to eliminate new HIV cases by the year 2020. This research will evaluate a new additional way to lower people's chances of getting HIV.

The drug being used in this project is called TRUVADA, and it is made by Gilead Sciences, Inc. TRUVADA is a single tablet made up of two different HIV medications: Viread (tenofovir disoproxil fumarate) and Emtriva (emtricitabine). Both these drugs have been widely used for many years to treat HIV. When used with other medicines in people who already ***have*** HIV, TRUVADA may help reduce the amount of HIV virus in the blood. It also may increase the number of cells in the blood that help fight off other infections. TRUVADA does not cure HIV or AIDS, and it is not an HIV vaccine.

In order for medicines to be used in Australia, they have to be approved by the Australian Federal Government. As a treatment for people who already have HIV, TRUVADA is approved for use in Australia, the United States, and other countries. As a medicine for PrEP, to lower chances of HIV in those who are not infected, TRUVADA has been approved in the US, but not in Australia. Therefore, in Australia, this study is considered experimental.

The purpose of the PRELUDE Study is to develop and evaluate a model of PrEP delivery in health services. It will see whether PrEP is an effective, safe and manageable option to help lower people's chances of getting HIV through sex. It will also study whether people in NSW find it easy and agreeable to take PrEP every day, why they take it, how it feels to take it, and whether it changes how they have sex. Finally, it will study how easy it is for doctors to use it with their patients, and what it costs the NSW government and clinics to provide PrEP.

This research study is being conducted by a group of investigators at the Kirby Institute, the University of New South Wales, and lead by Dr. Iryna Zablotska. It is funded by the NSW Ministry of Health. Gilead Sciences, who makes TRUVADA, is providing the study drug.

**How well does PrEP work?**

Research in other countries, including the U.S., has shown that the chances of getting HIV through sex is lowered for people who take PrEP ***and*** use condoms correctly during sex. How well PrEP works depends on how good people are at taking PrEP ***every day***.

Overall, studies in other countries have shown that taking PrEP lowers the risk of getting HIV by about 44 percent in homosexual, bisexual, and other men who have sex with men, and by about 70 percent in heterosexual adults. This means that some people on these research studies still got infected with HIV even though they were taking PrEP. However, when researchers looked specifically at people who were taking PrEP every day, they found that the risk of getting HIV was lowered even more. In one study of 2499 homosexual, bisexual and other men who have sex with men, those who took their PrEP pill every day had their risk of getting HIV lowered by 92 percent. In contrary, the risk of HIV infection was higher in people who did not take their tablets daily.

# 3 What does participation in this research involve?

Your involvement in the PRELUDE study will last up to about 36 months (3 years). This includes up to two weeks for screening, up to 30 months of taking PrEP(please note that the original period on PrEP up to 12 months is now extended to up to 30 months) and the completion of one follow-up visit and two follow-up questionnaires after you stop taking PrEP. The overall study will run for about five years.

By entering the study, you are not committing to being in the study and taking PrEP for the whole 30 months. For example, your participation in the study may end if you are no longer at risk of getting HIV through sex. You can also withdraw from the study at any time for your own reasons.

**Screening Visit**

The consent form must be signed by you before any study assessments are done.

First, doctors who are also researchers on this study will confirm that you are a good candidate for PrEP. . A number of tests will be conducted as a standard of care in New South Wales, Australia.

You will be tested for sexually transmitted infections, including HIV, chlamydia, gonorrhoea and syphilis. For gonorrhoea and chlamydia testing for men, this will be done by performing a swab of your rectum and collecting urine. Blood will be collected for syphilis, HIV testing and Hepatitis B testing.

For gonorrhoea and chlamydia testing for women, this will be done by performing [*sites to fill in method*: *a swab of your cervix, and a swab of your rectum if you have had anal sex in the last year AND/OR will be done by collecting urine]*. Blood will be collected for syphilis, HIV testing and Hepatitis B testing .

Your health and blood will be checked to make sure you are not at increased risk of possible side effects from TRUVADA, such as kidney or liver problems. You will also have a medical examination and will be asked about your medical history. If you are a woman who can get pregnant, you will have a pregnancy test before starting PrEP and while taking it, so that you and your doctor can together make an informed decision whether you start and continue taking PrEP.

Because both of the drugs in TRUVADA work against hepatitis B, you will be tested to know if you have active hepatitis B. This will allow doctors to recommend you an appropriate treatment. You may also be offered a vaccine against hepatitis B if the result of this test is negative. If the results show you were exposed earlier to hepatitis B and the infection has resolved, then you can be in the study. You may also be tested for Hepatitis C if this is considered standard at the clinic.

For all of these tests, [site to insert volume in millilitres and teaspoons or tablespoons, e.g. x tablespoons/y millilitres] of blood will be collected from a vein in your arm.

The samples collected for the standard of care tests will include: [site to insert accordingly.]

In order to qualify for the study, you must:

- Be at least 18 years old
- Have a negative HIV test within seven days of starting PrEP
- Not show possible signs of having HIV
- Be at high and ongoing risk for getting HIV
- Live in NSW, or visit NSW enough to attend all of your follow-up visits
- Be eligible for Medicare
- Be able to take TRUVADA once every day for at least 30 days
- Be able to attend clinic follow-up visits
- Be willing to take a computer-based survey about your attitudes, behaviours and lifestyle at the beginning of the study, after each follow-up visit, and twice after you stop taking PrEP.
- Be willing and able to consent to this study.

You will not be allowed to be enrolled in this study if you have certain kidney or liver problems, if you have to take certain medications that might make PrEP unsafe, if you have known allergies to PrEP, or if you are breastfeeding. You also cannot be in this study if you have any conditions that would make it difficult for you to take PrEP every day, such as certain mental health issues, memory loss, difficulty thinking or some intellectual disabilities. If you will be in prison or if you plan to be away from NSW for a period of 3 months or longer during the study, you cannot be in this study because you will not be able to attend your follow-up visits.

In addition, the study doctor may ask you whether you have any symptoms consistent with a new HIV infection and you have experienced any known or suspected exposure to HIV during the last 30 days. If you have, you will receive additional HIV testing, and may be asked to delay starting PrEP for up to one month until the conclusive result of that testing. **It is very important for your safety during this study that you tell the doctor if you are experiencing any of the following at screening or before you start PrEP. It is also very important that you tell your doctor about any of these at your follow-up visits. If you do but it is not time for your regularly-scheduled follow-up visit, please contact your doctor or the study coordinator.**

- Fever
- Feeling tired (fatigue)
- Muscle aches (myalgia)
- Joint aches (arthralgia)
- Skin rash
- Headache
- Sore throat (pharyngitis)
- Night sweats
- Swelling of the lymph nodes around the head and neck (cervical adenopathy)

There may be a few days between the time you start screening for this study, and the time you can start taking PrEP medication. If you encounter a high-risk sexual event before you start taking TRUVADA, please tell your study doctor immediately, so that your study doctor can assess whether you need to receive medication for “Post-Exposure Prophylaxis,” or PEP before you start taking PrEP. This will not affect your ability to start taking PrEP once you finish with PEP if you are eligible.

When you first asked about this research, the clinic doctor collected some basic information about who is interested in PrEP and why (age, gender, sexual orientation, and answers to the HIV risk questions). You also completed an internet based survey on attitudes, behaviour and lifestyle. The pre-screening questionnaire did not contain any identifiable information. If you decide to be in this study, you will be asked to consent to allow that information to be linked with your PRELUDE study information. The study will also collect information about any PEP you may have taken right before starting PrEP.

**Starting PrEP**

After you have met all study criteria, you will be given a prescription for 30 days of TRUVADA. You will be asked to start taking TRUVADA as PrEP immediately. Your doctor will provide you with information to help you remember to take TRUVADA every day. You will also receive condoms, and counselling on condom use and safe sex.

It is important to know that:

- You will be expected to take TRUVADA every day, as per the instructions
- You will be encouraged to practice safe sex, including consistent and correct use of condoms
- You will have regular HIV and STI testing, at each quarterly follow-up visit and as needed between study visits.

**How do I take PrEP?**

A tablet of TRUVADA must be taken by mouth once a day, at about the same time every day, and it is recommended to be taken with food. For this study, you will be asked to take PrEP daily for at least 30 days. You may continue to take PrEP every day for up to 30 months in total (please note that the original period on PrEP up to 12 months is now extended to up to 30 months)

If you miss a dose of TRUVADA, your doctor may advise you to take the missed pill as soon as you remember on that same day, but to not take more than one dose of TRUVADA in a 24 hour period. Your doctor may also advise that if you miss a dose, and it is almost time for your next dose, to wait and take the next dose at your regular time. Contact your medical provider as soon as possible if you take more than one pill of TRUVADA a day, to avoid overdosing.

TRUVADA should be stored at room temperature in its original container. The container should be kept tightly closed and out of the reach of children. Do not give TRUVADA prescribed to you to other people.

**Follow-up Visits while taking PrEP**

After you start the study, you will attend a follow-up visit at the clinic one month after you start taking PrEP, and then every three months after you start taking PrEP, for up to 30 months (that is the original period of taking PrEP up to 12 months is now extended to up to 30 months). At each of these visits, you will have an HIV test. It is very important that you take these HIV tests because TRUVADA cannot be taken alone by people who are HIV-positive (see HIV Infection section below).

At each follow-up visit you will be asked about your health, how well you remembered to take TRUVADA, and your sexual practices including safer sex practices. Each study visit will be about 15 minutes longer than your regular clinic visits.

At each of the quarterly visits (every three months), you will again be tested for HIV and sexually transmitted infections, and will be tested for your kidney and liver health. A total of about [site to insert volume in millilitres and teaspoons or tablespoons, e.g. x tablespoons/y millilitres] of blood will be collected from a vein in your arm for these tests. If you are a woman who can get pregnant, you will also be given a pregnancy test. You will be reminded about how to take PrEP, and will be given safe sex information, services, or referral for any services you might need.

If you are still eligible to continue taking PrEP, you will be given a new prescription for TRUVADA at these visits.

All of these follow-up procedures and discussions are considered standard care for people who are at risk for HIV infection and/or are taking anti-HIV medications. This means that even if PrEP wasn't considered research, these things would be done for your health and safety.

Within two and no more than within seven days of each clinic visit while you are taking PrEP, you will complete a self-administered, web-based, online questionnaire. The questionnaire will take approximately 30 minutes to complete. If you do not have a home computer that you are able to use, then you will be provided an alternate means of completing the questionnaire**.** The survey does not work well on mobile devices, so should be completed on a desktop or laptop computer. The questions will ask about things like your relationships, sexual practices and other related behaviour (in the preceding three months or since your last online questionnaire), your beliefs and attitudes about HIV and its prevention, and your experience with taking PrEP. It will also ask about how often you have taken your study medications since your previous online questionnaire. This includes information on when you have taken or missed doses of PrEP and when you have had and had not use condoms. The more accurate the information, the more useful it will be for the research and future use of PrEP.

The answers to the online questionnaire go directly to the researchers at the Kirby Institute. They will not share any of your survey answers with your clinic, doctors, nurses or other staff. The study researchers

are using the answers from these surveys to see how PrEP changes peoples' lives in general, not at an individual level.

**Stopping PrEP**

It is your right as a research participant to decide to stop taking PrEP at any time. During the 30-month period, you may choose to stop the PrEP medication if you feel that you no longer need it. Your study doctor may also have you stop the PrEP medication if she or he feels it is not needed, or it is not safe for you stay on PrEP. For example, you will be stopped from the study if you become HIV positive, if you start to experience kidney, liver or other unacceptable health problems, or if you need medication that interacts with TRUVADA.

It is strongly recommended for your safety that you take PrEP for at least 30 days at a time. Your doctor will discuss with you how to plan to stop PrEP during the study period if you need to. This may also involve taking TRUVADA as "Post-Exposure Prophylaxis," or PEP, if you and your doctor find out that you might have been exposed to HIV. If at any time during the study you stop taking PrEP and start taking PEP, the results of your HIV, STI and other tests taken because you are on PEP will be collected for the study as well. If you become HIV positive during this study, the results of any additional tests about your HIV infection, such as tests of resistance to different HIV treatments, will be collected for this study.

When you stop taking PrEP at any time during the study (including at 30 months), you will attend an end-of-study visit. At this visit, all quarterly follow-up visit procedures will be conducted.

If you change your mind about stopping TRUVADA and want to start taking it again, you can re-enter the study within the six months after you stopped, as long as you still qualify and the study is still open for re-enrolment. The option to re-enter will not be available during the last four months of the study or if the study has been filled.

**Follow-up Visits after you stop taking PrEP**

Three and six months after you stop taking PrEP, you will complete the on-line questionnaires for the researchers at Kirby, as already described. You will also be asked if the researchers can have access to your information from the NSW HIV Registry up to six months after you stop taking PrEP. That will allow the researchers to see if you test positive for HIV after you stop taking PrEP.

**Additional costs and reimbursement**

You will not be paid and there will be no additional costs to you to be in this project. All tests and medical care required as part of the research project will be provided to you as it normally would be through Medicare. During the 30 month period, the medication will be provided free-of-charge by Gilead Sciences. Because this study wants to know what it will be like to use PrEP in the real-world, you will not be reimbursed for your travel to the clinics, clinic parking, your time, or other expenses associated with the research project.

This research project has been designed to make sure the researchers interpret the results fairly and appropriately. It has also been designed to keep study doctors or participants from jumping to conclusions.

# 4 What do I have to do?

Participation in this study does not require any changes to your diet or to your participation in sports. It is important let the study doctor know any other medications you are already taking or start taking while on TRUVADA.

Even if this was not a research study, all people who take PrEP will need to do other things to prevent getting infected with HIV and other sexually transmitted infections.

People who take PrEP must:

- **Take PrEP every day**. Not taking PrEP every day can increase your risk of getting HIV. Because this is so important, your doctor will talk with you about the best ways to help you remember to take your pill.
- **Continue using condoms every time when having sex:** PrEP is not 100 percent effective in preventing HIV, and does not protect against getting other sexually transmitted infections, like herpes, gonorrhoea, and syphilis.
- **Let the study doctor know if they have not been taking PrEP every day and may have been exposed to HIV:** When taken every day, PrEP can still take at least seven days to build up the maximum protection. If you think you have been exposed to HIV, your doctor will discuss your risk with you, and whether you may need Post-Exposure Prophylaxis. Do not wait until your next follow-up visit to discuss this with your doctor. It is important to start Post-Exposure Prophylaxis within 72 hours after you were exposed to HIV.

It is also important to know that:

- Taking extra pills will not provide you with extra protection.
- PrEP is not a “morning after” pill; it will not work if only taken right before or right after exposure to HIV.
- If you decide to stop taking PrEP for any reason, you should make sure you have taken PrEP every day for four weeks after the last time you may have had sex that put you at risk of HIV.
- If you have been exposed to HIV, your doctor may recommend to stop PrEP and take a course of Post-Exposure Prophylaxis.

# 5 Other relevant information about the research project

Participants in this study will be adults who are HIV-negative and are at risk of getting HIV through sex. The study allows “up to 300 person-years” of PrEP. This means that if everyone who participates takes one year of TRUVADA, there will be 300 people in the study. However, there may be more people in the study if at least some participants take TRUVADA for less than one year.

Everyone in the study will take TRUVADA for PrEP, and everyone will have the same study visits. The PRELUDE Study is open to all adults regardless of sex, gender or sexual preference. There will be between four and 12 sites involved in the project. The coordinating principal investigator for this project is Dr Iryna Zablotska. The PRELUDE Study Management Team is responsible for the day-to-day management and coordination of the study. Each clinic in the study also has investigators who are part of the study.

# 6 Do I have to take part in this research project?

Participation in any research project is voluntary. You do not have to be in this project if you do not want to. If you decide to be in this project now, it is your right to change your mind later. You are free to withdraw from the project at any time. If you do decide to take part, you will be given this Participant Information and Consent Form to sign and you will be given a copy to keep.

Your decision whether to take part or not to take part, or to take part and then withdraw, will not affect your routine treatment, your relationship with those treating you or your relationship with [insert Institution].

# 7 What are the alternatives to participation?

You do not have to be in this research project to receive treatment at this clinic. Other options to prevent HIV include: advice and counselling on safe sex practices including the use of condoms, and regular HIV and STI testing. HIV post-exposure prophylaxis (PEP) is available at your clinic after any sexual or injecting exposures that may have put you at risk of getting HIV. Your study doctor will discuss these options with you before you decide whether or not to take part in this research project. You can also discuss these options with your local doctor.

Other anti-HIV medications are currently being studied for use as PrEP. However, no other medications have been approved for the prevention of HIV.

# 8 What are the possible benefits of taking part?

There is no guarantee or promise that you will receive any benefits from being in this research project. Research shows that taking TRUVADA every day, combined with safer sex practices including using condoms during sex, may decrease your risk of getting HIV. Some participants may experience direct health benefits due to testing for HIV and STIs more regularly than they would normally otherwise. Participants may also like the idea that they are contributing to new knowledge about the prevention of HIV.

# 9 What are the possible risks and disadvantages of taking part?

Medications often cause side effects. You may have none, some, or all of the effects known to be associated with the use of TRUVADA and listed below. They may be mild, moderate or severe. If you have any of these side effects, or are worried about them, talk with your study doctor. Your study doctor will also be looking out for side effects.

There may be side effects that the researchers do not expect or do not know about which may be serious. Tell your study doctor immediately about any new or unusual symptoms that you experience.

Many side effects go away shortly after treatment ends. However, sometimes side effects can be serious, long lasting or permanent. If a severe side effect or reaction occurs, your study doctor may need to stop your treatment. Your study doctor will discuss the best way of managing any side effects with you.

**Possible Side Effects of TRUVADA**

TRUVADA has not been used in HIV negative people as much as it has been used in people who are HIV positive. In people who are HIV negative, it has only been used by several thousands of participants in clinical trials of PrEP and by HIV negative people taking it as part of Post-Exposure Prophylaxis or PEP. The dose of TRUVADA used in PEP and PrEP is the same, and in both cases it is taken as a daily tablet. People who take TRUVADA for PrEP and PEP usually take TRUVADA for shorter periods of time than those who take TRUVADA for HIV treatment. As well, people who take TRUVADA for HIV treatment most often take more than one HIV medication at the same time.

I**n people who did not have HIV and took TRUVADA for PrEP:** In previous research studies, between three and nine percent of such participants reported the following four side effects, and these levels were higher than among the participants who did not take TRUVADA (took a "sugar pill"):

- headache
- stomach pain
- involuntary weight loss
- nausea or upset stomach

In PrEP studies where these side effects were reported, these were modest, usually only occurred in the first one or two months of taking the drug and did not last.

**In people who *have* HIV who took TRUVADA:** the most common (reported in 10 percent and greater) side effects according to the company that makes TRUVADA are:

- diarrhoea
- nausea/upset stomach
- tiredness
- headache
- dizziness
- depression
- difficulty sleeping
- strange dreams
- rash

**Possible allergic reaction:** In clinical trials of TRUVADA, a small number of people have had an allergic reaction to tenofovir, one of the drugs in TRUVADA. Symptoms of an allergic reaction may include fever, rash, upset stomach, vomiting, loose or watery stools, stomach pain, achiness, shortness of breath, a general feeling of illness or a potentially serious swelling of the face, lips, and/or tongue.

The company that makes TRUVADA also warns that the following changes in laboratory tests are possible. This information is based mostly on experience with people who take TRUVADA for HIV treatment, and on other drugs that are like TRUVADA. Because HIV by itself can also cause these same problems, it is difficult to know how much TRUVADA alone contributes to kidney and bone disease:

- kidney problems
- decreases in the minerals in their bones
- build-up of lactic acid in the blood
- enlarged liver

Because the last two of these side effects are so serious, the symptoms of each are listed below. **Call your doctor immediately if you get these symptoms:**

| Symptoms of too much lactic acid in the blood | Symptoms of severe liver problems |
|---|---|
| • weakness or being more tired than usual<br>• unusual muscle pain<br>• being short of breath or fast breathing<br>• nausea, vomiting, and stomach-area pain<br>• cold or blue hands and feet<br>• feel dizzy or lightheaded<br>• fast or abnormal heartbeats | • your skin or the white part of your eyes turns yellow<br>• dark “tea-colored” urine<br>• light-colored stools<br>• loss of appetite for several days or longer<br>• nausea<br>• stomach-area pain |

**To manage kidney and liver health:** Your doctor will be taking blood tests to check you kidneys and liver before you start and while you are taking TRUVADA. Your study doctor may tell you to stop taking TRUVADA if you develop kidney or liver problems during the study.

**Bone health:**

People who have HIV infection have usually taken anti-HIV treatment for long periods of time and have taken more than one HIV medication including the type of drugs that are in TRUVADA. They have experienced a three- to four-percent decline in the minerals in their bones.

In HIV negative people on TRUVADA bone scans were taken only in two other PrEP research studies – one using TRUVADA and one using one out of the two drugs that make up TRUVADA. These scans found that less than one percent of study participants tested experienced a small decrease in the minerals in their bones during the first few months of PrEP. This decline either stopped getting worse or returned to normal after the first few months. No increase in bone breaks was observed. Because of this, bone scans are not recommended for HIV negative people in this study. However, if you have a

history of bone fractures/breaks or if you are at significant risk for osteoporosis, please tell the study doctor, who will help you manage this risk.

**HIV infection**

People with HIV need full HIV medication and treatment. You will have an HIV test at every follow-up visit. If you become infected with HIV, your study doctor will have you stop taking PrEP immediately. This is because taking TRUVADA by itself when you have HIV can make the virus resistant to some HIV drugs so that they may no longer work for you and limit your HIV treatment options over time.

**Hepatitis B infection**

If you become infected with Hepatitis B while you are taking TRUVADA, please tell your study doctor immediately, and do not stop taking TRUVADA on your own. If you stop TRUVADA while you have Hepatitis B, your hepatitis symptoms may get worse. You study doctor will help you stop taking TRUVADA and will monitor you for safety.

**Pregnancy and breastfeeding**

The effects of TRUVADA on an unborn child and on a newborn baby are not known. You may not participate in this study if you are breast-feeding at the start of the study. If you are female and you can possibly get pregnant, you must also take a pregnancy test at every follow-up visit. All participants are expected to continue use of safer sex practices [non-Catholic sites may add: including the use of condoms].

Any participants who are taking PrEP in conjunction with conception must take daily doses of TRUVADA beginning one month before a conception attempt. TRUVADA must continue to be taken daily until one month after the last attempt to conceive.

Women who become pregnant while in this study should make an informed decision about whether or not to keep taking PrEP while pregnant. If you become pregnant during this study, you should tell your study doctor immediately. Your study doctor will discuss with you your risks of HIV infection during pregnancy, the possible risks of TRUVADA on your pregnancy and child, and whether or not you should stay in the study.

If you become pregnant, we will ask for copies of or access to the parts of your medical records that discuss your pregnancy, delivery, and your infant's health.

**Other risks**

Having a blood sample taken may cause some discomfort, pain or bruising. Sometimes, the blood vessel may swell, or blood may clot in the blood vessel, or the spot from which blood is taken could become inflamed. Some people may feel dizzy when having blood taken, and may occasionally faint. Rarely, there could be a minor infection or bleeding. If this happens, it can be easily treated.

You may experience pain or discomfort from the rectal or cervical swab. In some cases, you may have some bleeding.

The questions we will ask you about your sexual behaviour may make you feel uneasy. However, you do not have to answer any question that you do not want to and you can stop answering the questions at any time.

Although your information will be kept confidential by study staff, it is possible that your friends, family or people in your community may find out that you are in this study. If that happens, you may experience stigma as a result of being involved in a study about HIV. If this or any other issue may make you become upset or distressed, the study doctor will be able to arrange for counselling or other appropriate support. Any counselling or support will be provided by qualified staff who are not members of the research project team. This counselling will be provided free of charge.

## 10 What will happen to my samples?

Your blood will be collected at the beginning of the study and at follow-up visits, to check your kidney and liver health, and to test for HIV, STI and hepatitis B infection. This is considered routine care for people who take TRUVADA for PrEP. The study team will know that these samples belong to you as they will be sent to the lab with your personal information, as they normally are for routine care.

If a test shows you have HIV or Hepatitis, you will have follow-up counselling and appropriate medical advice. If your test results are positive, the study doctors are required by law to notify government health authorities. Signing the consent form means that you agree to have this testing; it will not be done without your consent.

## 11 What if new information arises during this research project?

Sometimes during a research project, new information becomes available about the medication that is being studied. If this happens, your study doctor will tell you about it and will discuss with you whether you want to continue. If you decide to withdraw, your study doctor will make arrangements for your regular health care to continue. If you decide to continue in the research project you may be asked to sign an updated consent form that has the new information in it.

Also, on receiving new information, your study doctor might consider it to be in your best interest to withdraw you from the research project. If this happens, he/ she will explain the reasons and arrange for your regular health care to continue.

## 12 Can I have other treatments during this research project?

It is not anticipated that TRUVADA will affect any other medications you may be taking. However, it is important to let the study doctor know about any other medications or treatments you may be taking and/or using, including over-the-counter medications, vitamins or herbal remedies, acupuncture or other alternative treatments. You should also tell your study doctor about any changes to these during your time in the research project. Your study doctor will explain to you which treatments or medications cannot be used if you are involved in the PRELUDE study.

## 13 What if I withdraw from this research project?

If you decide to withdraw from this project, please let a member of the research team know before you do so. This will allow you to find out about any possible health risks or special requirements before you stop.

If you do withdraw your consent during this research project, the study team will ask you to continue to complete the attitude, behavioural and lifestyle questionnaires online. All the personal information that they have already collected for this study will be kept so that the results of the research can be measured properly, and to comply with law. You should be aware that data collected up to the time you withdraw will form part of the research project results. If you do not want this to happen, you must tell the researcher before you join the PRELUDE study.

If you choose to stop taking PrEP, you can still agree to complete the discontinuation and 3 months post-discontinuation follow-up visits, to complete discontinuation and post-discontinuation online surveys, and/or to allow the team to check your HIV status six months after you discontinue. If you agree to any of these activities after you stop PrEP, please do not complete the revocation of consent form until these follow-up activities are completed.

## 14 Could this research project be stopped unexpectedly?

This research project may be stopped unexpectedly for reasons which may include:

- Unacceptable side effects
- PrEP being shown not to be effective
- Decisions by local regulatory/health authorities.

## 15 What happens when the research project ends?

PrEP will be available to you for up till the end of the study. At the present time TRUVADA is not registered for use in Australia. At the end of this study Gilead Sciences may not agree to continue to supply TRUVADA, or may agree to provide an ongoing supply but only under certain conditions and for a limited time period. At the end of the study, if the study drug is still not registered in Australia AND subsidised on the PBS for your condition, then [*insert study site name*] will not be able to fund ongoing supplies of the drug. TRUVADA may never be registered in Australia for PrEP. This means that TRUVADA may not be available or may be expensive for you to buy. Your study doctor will discuss with you other options to help prevent HIV infection.

We estimate that all participants in the study will have completed the study by the end of 2018. After this time, the data will be analysed and a one-page summary of results will be prepared for all participants. Study staff will send copies of the summary to each participating clinic and you will be given the summary at your next routine clinic visit with your doctor.

# Part 2 How is the research project being conducted?

## 16 What will happen to information about me?

### How information is used and stored

By signing the consent form you agree to let the study doctor and related research staff collect and use personal information about you for the research project. Any information collected for this research project that can identify you will remain confidential.

Information collected for this research will be kept in two different databases:

***Health information:*** Health information collected about you for this research will be entered into and stored in a study-specific password-protected database. This information will be coded, which means that your name won't be included. However, the study staff can link this information back to the rest of your personal information, such as your clinic medical records or Commonwealth and State agency health and disease-related registries, by using a decoding key containing your date of birth. Your research health information may also be recorded in your health records at the clinic. If at any time during the study your study doctor requests additional HIV testing beyond the standard screening test, copies of these additional test results will be collected so that the researchers can better understand the HIV testing processes involved with PrEP management. Your name and all identifying information will be blocked out and your study ID number will be written on any photocopies collected by the researcher.

***Behavioural survey information:*** Only the researchers at the Kirby Institute will have access to your internet-based survey information. This information will be stored in a password-protected database (SurveyGizmo), and will be treated as confidential and securely stored. All participants will be allocated a study number, and all electronic data will be stored under this code, so your name and identifying information will not be stored with the information collected about you during the study. The behavioural

survey system will store your preferred email address so that the system can send you invitations to compete the surveys, and automatic reminders if you do not complete a survey within the prefered time frame. Your email address is stored separately from your clinical information. Your name is not stored in any database. Only one member of the research team at the Kirby Institute, who is responsible for managing the behavioural survey system, will be able to access the database; s/he has signed the confidentiality agreement and s/he does not have access to any other personal identifying information or to your clinical records. Furthermore, because your email address is stored only for follow-up purposes, it will be erased from the system as soon as you complete your final follow-up survey or when you withdraw your consent to participate in the study, whichever comes first.

All electronic databases kept at the Kirby Institute will be protected by passwords and University of New South Wales firewalls. Identifying details (such as your name and contact details) and the fact that you are participating in this study will be known only to: your doctor; the research nurse/s at your clinic; and the project leader and research assistant based at the Kirby Institute, UNSW, Sydney. The project leader and research assistant at the Kirby Institute will need your name and contact details so as to assist with follow-up and provide you with information about the study as it progresses.

The researchers at the Kirby Institute will not share any of your survey answers with your clinic doctors, nurses or other staff. However, if you do not complete a behavioural survey within the preferred time frame nor after you have received two electronic reminders, the Kirby Institute researchers may ask the study staff to contact you to provide another reminder. If you do not respond to these reminders, we will consider that you are not willing to take a computer-based survey. Please note that in order to qualify for the study, you must, among other criteria, be willing to take a computer-based survey about your attitudes, behaviours and lifestyle at the beginning of the study, after each follow-up visit, and twice after you stop taking PrEP. You may, therefore, no longer receive an extension for access to PrEP within the study. However, you will be able to receive a prescription for PrEP and access it outside the study.

Any paper based forms used for this study will be stored in a locked office, which only the members of the study team can access.

Your information will only be used for the purpose of this or other related research, and it will only be disclosed with your permission, except as required by law. Coded data may be used in future related research.

Information about you may be obtained from your health records held at this and other health services for the purpose of this research. By signing the consent form you agree to the study team accessing health records if they are relevant to your participation in this research project.

Any information obtained for the purpose of this research project and for any future related research described in Part 16 that can identify you will be treated as confidential and securely stored. It will be disclosed only with your permission, or as required by law.

**Inspection of research records**

It is possible that your health research study records may be inspected in order to make sure that the study is being conducted appropriately and safely, and that the information is being reported truthfully. Possible inspectors include: representatives from the Kirby Institute, relevant authorities and authorised representatives of the Kirby Institute, the institution relevant to this Participant Information Sheet, *[Name of institution]*, or as required by law. By signing the Consent Form, you authorise release of, or access to, this confidential information to these relevant study personnel and regulatory authorities.

**Publication and presentation of research findings**

The findings from this research project will be published and/or presented in a variety of places, most likely combined with everyone else's information. In any publication and/or presentation, information will be provided in such a way that you cannot be identified, except with your permission.

**Your rights to your information**

In accordance with relevant Australian and New South Wales privacy and other relevant laws, you have the right to request access to your information collected and stored by the research team. You also have the right to request that any information with which you disagree, be corrected. Please contact the study team member named at the end of this document if you would like to access your information.

**Information storage after the study**

After the end of the study, your study files will be archived in a locked storage facility and kept for at least 15 years after the end of the study. Coded data in electronic form will also be stored for this amount of time.

# 17 Complaints and compensation

If you suffer any injuries or have complications as a result of this research project, you should contact the study team as soon as possible and you will be assisted with arranging appropriate medical treatment.

You may have a right to take legal action to obtain compensation for any injuries or complications resulting from your participation in the study. Compensation from the sponsor of this study, the University of New South Wales, may be available if your injury or complication is caused by the drugs or procedures, or by the negligence of any of the parties conducting the study. If you receive compensation that includes an amount for medical expenses, you will be required to pay for your medical treatment from those compensation monies.

If you are not eligible for compensation for your injury or complication under the law, but are eligible for Medicare, you can receive any medical treatment required to treat the injury or complication, free of charge, as a public patient in any Australian public hospital.

The people running this study agree to follow the Medicines Australia *Guidelines for Compensation for Injury Resulting from Participation in an Industry-Sponsored Clinical Trial*. These Guidelines allow for some claims for compensation to be settled without the need for legal action to be taken. The fact that the people running this study have agreed to abide by these guidelines in respect of the clinical trial does not affect your rights to pursue legal action in respect of any injury you may suffer as a result of participation. You can obtain a copy of these Guidelines from the Secretary of the Human Research Ethics Committee."

# 18 Who is organising and funding the research?

This research project is being conducted by the Chief Investigator, Dr Iryna Zablotska at the Kirby Institute, and is being funded by the NSW Ministry of Health. The manufacturer of TRUVADA, Gilead Sciences, is providing the study drug.

[*Insert site name*] will receive payments from the NSW Ministry of Health for time spent undertaking this research project. No member of the research team will receive personal financial benefit from your involvement in this research project (other than their ordinary wages).

Gilead Sciences may benefit financially from this research project if, for example, the project assists Gilead to obtain approval for TRUVADA to be used as PrEP in Australia.

In addition, if knowledge acquired through this research leads to discoveries that are of commercial value to Gilead Sciences, the study doctors or their institutions, there will be no financial benefit to you or your family from these discoveries.

# 19 Who has reviewed the research project?

All research in Australia involving humans is reviewed by an independent group of people called a Human Research Ethics Committee (HREC). The ethical aspects of this research project have been approved by the HREC of the St. Vincent's Hospital, Darlinghurst NSW. This project will be carried out according to the National Statement on Ethical Conduct in Human Research (2007). This statement has been developed to protect the interests of people who agree to participate in human research studies.

# 20 Further information and who to contact

The person you may need to contact will depend on the nature of your query. If you want any further information about this project or if you have any medical problems which may be related to your involvement in the project (for example, any side effects), you can contact the study chief investigator Dr. Iryna Zablotska on 9385-0951 or any of the following people:

**Clinical contact person (for each study site to complete; may provide more than one if necessary)**

| | |
|---|---|
| Name | *[Name]* |
| Position | *[Position]* |
| Telephone | *[Phone number]* |
| Email | *[Email address]* |

For matters relating to research at the site at which you are participating, the details of the local site complaints person are:

**Complaints contact person (for each study site to complete)**

| | |
|---|---|
| Name | *[Name]* |
| Position | *[Position]* |
| Telephone | *[Phone number]* |
| Email | *[Email address]* |

If you have any complaints about any aspect of the project, the way it is being conducted or any questions about being a research participant in general, then you may contact:

**Reviewing HREC approving this research and HREC Executive Officer details**

| | |
|---|---|
| Reviewing HREC name | St. Vincent's Hospital Sydney HREC |
| HREC Reference number | HREC/14/SVH/130 |
| HREC Executive Officer | Contact person - HREC Executive Officer |
| Telephone | 8382 2075 |
| Email | research@stvincents.com.au |

**Local HREC Office contact (Single Site -Research Governance Officer – for each study site to complete)**

| | |
|---|---|
| Name | *[Name]* |
| Position | *[Position]* |
| Telephone | *[Phone number]* |
| Email | *[Email address]* |

# Consent Form

| | |
|---|---|
| **Title** | Implementation of HIV preexposure prophylaxis with antiretroviral medications among people at high risk for HIV infection: A demonstration project |
| **Short Title** | The PRELUDE Study |
| **Protocol Version** | 30 September 2015 |
| **Protocol Number** | HEPP 1403 |
| **Project Sponsor** | The Kirby Institute for Infection and Immunity in Society |
| **Coordinating Principal Investigator** | Dr. Iryna Zablotska, The Kirby Institute |
| **Associate Investigator(s)** | [insert if required by institution] |

## Declaration by Participant: Consent to be in the PRELUDE Study

I have read the Participant Information Sheet or someone has read it to me in a language that I understand.

I understand the purposes, procedures and risks of the research described in the project.

I give permission for my doctors, other health professionals, hospitals or laboratories outside this hospital to release information to *[Insert site name here]* concerning my disease and treatment for the purposes of this project. I understand that such information will remain confidential.

I give permission to link my details using a unique identifying code with the Commonwealth and State agency health and disease-related registries. Such registries include, but are not limited to, registries of HIV, STIs and use of HIV post-exposure prophylaxis. This information will allow more accurate ascertainment of HIV and STI diagnoses, as well as the use of antiretroviral medications outside of the demonstration project PRELUDE.

I have had an opportunity to ask questions and I am satisfied with the answers I have received.

I freely agree to participate in this research project as described and understand that I am free to withdraw at any time during the study without affecting my future health care.

I understand that I will be given a signed copy of this document to keep.

I understand that if I decide to discontinue the study treatment, I may be asked to attend follow-up visits to allow collection of information regarding my health status. Alternatively, the investigator/sponsor will request my permission to access my medical records for collection of follow-up information for research and analysis.

Please ***initial*** one option for the following (yes or no):

I agree that the Kirby Institute may contact me about future research projects that I may qualify for.

(initial one option) __________Yes __________No

| | |
|---|---|
| Name of Participant (please print) | ______________________________ |
| Signature ______________________ | Date ______________________ |

| | |
|---|---|
| Name of Witness* to Participant's Signature (please print) | ______________________________ |
| Signature ______________________ | Date ______________________ |

* Witness is not to be the investigator, a member of the study team or their delegate. In the event that an interpreter is used, the interpreter may not act as a witness to the consent process. Witness must be 18 years or older.

**Declaration by Study Doctor/Senior Researcher**

I have given a verbal explanation of the research project, its procedures and risks and I believe that the participant has understood that explanation.

| | |
|---|---|
| Name of Study Doctor/ Senior Researcher† (please print) | ______________________________ |
| Signature ______________________ | Date ______________________ |

† A senior member of the research team must provide the explanation of, and information concerning, the research project.

Note: All parties signing the consent section must date their own signature

PRELUDE Participant Consent Form 30 September 2015 v5.0 Page 2 of 3

# Form for Withdrawal of Participation

| | |
|---|---|
| **Title** | Implementation of HIV preexposure prophylaxis with antiretroviral medications among people at high risk for HIV infection: A demonstration project |
| **Short Title** | The PRELUDE Study |
| **Protocol Version** | 02 October 2015 |
| **Protocol Number** | HEPP 1403 |
| **Project Sponsor** | The Kirby Institute for Infection and Immunity in Society |
| **Coordinating Principal Investigator** | Dr. Iryna Zablotska, The Kirby Institute |
| **Associate Investigator(s)** | [insert if required by institution] |

## Declaration by Participant

I wish to withdraw from participation in the above research project and understand that such withdrawal will not affect my routine treatment, my relationship with those treating me or my relationship with *[insert clinic/Institution]*.

| |
|---|
| Name of Participant (please print) ______________________________ |
| Signature ______________________ Date ______________________ |

*In the event that the participant's decision to withdraw is communicated verbally, the Study Doctor/Senior Researcher will need to provide a description of the circumstances in the participant's source documentation.*

## Declaration by Study Doctor/Senior Researcher†

I have given a verbal explanation of the implications of withdrawal from the research project and I believe that the participant has understood that explanation.

| |
|---|
| Name of Study Doctor/<br>Senior Researcher† (please print) ______________________________ |
| Signature ______________________ Date ______________________ |

† A senior member of the research team must provide the explanation of and information concerning withdrawal from the research project.

Note: All parties signing the consent section must date their own signature.

[Clinic Logo]

# PARTICIPANT INFORMATION SHEET AND CONSENT FORM

# BLOOD SAMPLE COLLECTION AND STORAGE

### St Vincent's Centre for Applied Medical Research Biobank

| | |
|---|---|
| **Title** | Implementation of HIV preexposure prophylaxis with antiretroviral medications among people at high risk for HIV infection: A demonstration project |
| **Short Title** | The PRELUDE Study |
| **Project Sponsor** | The Kirby Institute for Infection and Immunity in Society |
| **Coordinating Principal Investigator** | Dr. Iryna Zablotska, The Kirby Institute |
| **Associate Investigator(s)** | *[Site: insert if required by institution]* |

## Request

You are invited to take part in the optional collection and storage of extra blood samples because you are a participant in the PRELUDE Study.

We ask that you consider giving your permission for storage of a sample of your blood at the St Vincent's Centre for Applied Medical Research laboratory for possible use in future research.

Please read this information carefully. Ask questions about anything that you don't understand or want to know more about. Before deciding whether or not to take part, you might want to talk about it with a friend, partner, family member or your doctor.

Participation in this optional 'blood banking' is voluntary. If you don't wish to take part, you don't have to. Should you choose not to participate in this optional "blood banking" component, this will not affect your participation in the main study.

This form provides you with information to help you decide whether you will allow this.

*St Vincent's Centre for Applied Medical Research Biobank*
*21 October 2014 version 3.0 Tissue Banking Patient Information Sheet*

**What kind of sample will be taken, and how?’**

As part of the main PRELUDE Study, you will have an extra sample of blood taken for storage at the one-month follow-up visit, and later at months 6, and 12 (about ten millilitres or two teaspoons). This will be collected by a trained nurse from a vein in your arm. If you take a break from taking TRUVADA® for any reason, this schedule may change to accommodate that break. However, you will have no more than three collections of this blood during the study,

The blood will be processed in a laboratory. The remaining substance, called serum, will then be stored for possible future research use.

If you agree to participate in this blood sample storage sub-study, you will be consenting to allowing the blood samples to be stored and analysed beyond the end of the PRELUDE Study. The samples will be stored indefinitely.

**2. ‘Will the sample be identifiable as mine after it is stored?’**

Your personal identifiers, such as your full name and date of birth, will be removed, and this will be replaced with a unique code on all sample labels. However, these stored tissue samples could be re-identifiable as yours.

Any identifiable information that is collected about you in connection with this study will remain confidential and will be disclosed only with your permission, or except as required by law.

All participants will be allocated a study number, and all electronic data will be stored under this code, so your name and identifying information will not be stored with the information collected about you during the study. All electronic databases kept at the Kirby Institute will be protected by passwords and University of New South Wales firewalls. Identifying details (such as your name and contact details) and the fact that you are participating in this study will be known only to: your doctor; the research nurse/s at your clinic; and the project leader and research assistant based at the Kirby Institute, UNSW.

**3. ‘What will happen to my sample?’**

Your sample will be stored at the St Vincent’s Centre for Applied Medical Research Biobank, Sydney, indefinitely.

We wish to store (or ‘bank’) the sample for potential, and as yet unspecified, research in the future. Not all potentially beneficial future research can be known at any one time, as the need for future research is determined by ongoing developments in the field. If you agree to your sample being stored, you will be asked to sign a specific consent form to store your sample in this way.

*St Vincent’s Centre for Applied Medical Research Biobank*
*21 October 2014 version 3.0 Tissue Banking Patient Information Sheet*

**4. 'How will I know if my samples are being used in the future?'**

If you agree to your sample/s being stored for future research, they may be used for research projects in the future with the approval of a Human Research Ethics Committee. The information obtained from future tests will not reveal anything about your health status. Therefore, neither you nor your doctors will be contacted by the Researchers in connection with the research or any information about the results of tests performed on the sample that you donate for this sub-study.

It may be possible to provide you with feedback about the findings of potential future research, should you agree to be contacted by the Kirby Institute when the study is completed.

**5. 'Who will have access to my tissue sample once it has been stored?'**

The research team responsible for ensuring appropriate standards are met in storing and managing your specimens will have access to your sample/s. Researchers involved in research approved by a Human Research Ethics Committee may also have access to your sample.

**6. 'Will drug or biotechnology companies be able to use my sample for profit in the future?'**

There is the possibility that research involving your blood sample may result in commercially viable technology or treatments. You will not however be able to claim financial benefit from any discoveries arising from the use of your sample.

**7. 'How long will my tissue sample be stored?'**

Your sample will be stored indefinitely.

**8. 'Will I be able to get my sample back if I change my mind once it has been stored in the 'tissue bank'?'**

You may contact your study doctor at any time and request that your sample be destroyed, where this can be done.

**9. 'Who should I contact if I have concerns about the conduct of this study?'**

This study has been approved by St Vincent's Hospital HREC. Any person with concerns or complaints about the conduct of this study should contact the

Research Office who is nominated to receive complaints from research participants. You should contact them on 02 8382 2075 and quote HREC/14/SVH/130.

The conduct of this study at the [*name of site*] has been authorised by the [*name of organisation*]. Any person with concerns or complaints about the conduct of this study may also contact the [*Research Governance Officer* or *other officer*] on [*telephone number*] and quote reference number [*insert SSA reference number*]

**Thank you for taking the time to consider this study. If you wish to take part in it, please sign the attached consent form. This information sheet is for you to keep.**

[Clinic Logo

# BLOOD SAMPLE COLLECTION AND STORAGE
## [To be used in conjunction with a Participant Information Sheet]

### St Vincent's Centre for Applied Medical Research Biobank

| | |
|---|---|
| **Title** | Implementation of HIV preexposure prophylaxis with antiretroviral medications among people at high risk for HIV infection: A demonstration project |
| **Short Title** | The PRELUDE Study |
| **Project Sponsor** | The Kirby Institute for Infection and Immunity in Society |
| **Coordinating Principal Investigator** | Dr. Iryna Zablotska, The Kirby Institute |
| **Associate Investigator(s)** | *[Site: insert if required by institution]* |

1. I,..........................................................................................................
of..........................................................................................................
agree to donate my blood/tissue as described in the Participant Information Sheet set out above***.***

2. I acknowledge that I have read the Participant Information Sheet, which explains why I have been asked to donate blood/tissue. The nature and risks of this blood sample storage bank have been explained to me to my satisfaction.

3. Before signing this consent form, I have been given the opportunity of asking any questions relating to any possible physical and mental harm I might suffer as a result of my participation and I have received satisfactory answers.

4. I understand that I ***may*** be able to request my stored samples be destroyed, and this will not prejudice to my relationship to the Kirby Institute, the University of New South Wales, [insert site name], St Vincent's Centre for Applied Medical Research Biobank, or my doctor.

5. I agree that research data gathered relating to my samples may be published, provided that I cannot be identified.

6. I understand that if I have any questions relating to my participation in this tissue bank, I may contact [Clinician's name].on telephone [insert telephone number]., who will be happy to answer them.

7. I acknowledge receipt of a copy of this Consent Form and the Participant Information Sheet.

Please ***initial*** one option for the following (yes or no):

I wish to be contacted with feedback about the findings of potential future research that is done using my banked samples.

(initial one option) __________Yes __________No

Complaints may be directed to the, Research Office on 02 83822075

**Signature of participant** **Please PRINT name** **Date**

---

**Signature of witness** **Please PRINT name** **Date**

---

**Signature of investigator** **Please PRINT name** **Date**

---

*St Vincent's Centre for Applied Medical Research Biobank*
*21 October 2014 version 3.0 Tissue Banking Patient Information Sheet*

[Clinic Logo]

# BLOOD SAMPLE COLLECTION AND STORAGE

## St Vincent's Centre for Applied Medical Research Biobank

| | |
|---|---|
| **Title** | Implementation of HIV preexposure prophylaxis with antiretroviral medications among people at high risk for HIV infection: A demonstration project |
| **Short Title** | The PRELUDE Study |
| **Project Sponsor** | The Kirby Institute for Infection and Immunity in Society |
| **Coordinating Principal Investigator** | Dr. Iryna Zablotska, The Kirby Institute |
| **Associate Investigator(s)** | *[Site: insert if required by institution]* |

## REVOCATION OF CONSENT

I hereby wish to **WITHDRAW** my consent to participate in the blood sample storage bank described above and understand that such withdrawal **WILL NOT** jeopardise any treatment or my relationship with the Kirby Institute, the University of New South Wales, [insert site name], St Vincent's Centre for Applied Medical Research Biobank, or my doctor.

Signature Date

Please PRINT Name

The section for Revocation of Consent should be forwarded to **(INSERT name and full address of Principal Investigator).**
