# Supplementary material for: Validation of participant eligibility for pre-exposure prophylaxis: Baseline data from the PRELUDE demonstration project
Source: PLoS One. 2017 Sep 26;12(9):e0185398. doi: 10.1371/journal.pone.0185398 (PMC5614574; doi:10.1371/journal.pone.0185398)
Supplement: S3 File — (DOCX) [file pone.0185398.s004.docx]

# S3 File: *PRELUDE* baseline behavioural survey

## WELCOME

Thank you for participating in the PRELUDE study. Your time and participation are highly valued.

You are asked to complete this survey as you have been invited or enroled to participate in the PRELUDE study. The aim of this survey is to gather information about the characteristics, beliefs, attitudes and behaviour of people who have been enrolled into the study to take a daily anti-HIV medication as pre-exposure prophylaxis of HIV (PrEP).

This questionnaire will record your study ID number only and all answers will remain confidential at all times. Please note that this questionnaire collects information through a totally separate system to that used in clinics. Therefore, nurses, doctors and any other staff of the clinics participating in this study will not have access to any information you provide below.

This questionnaire should be completed within 48 hours of your baseline visit to the study clinic (that is the visit when you received your first study medication prescription). If necessary you may save your responses and continue at another time. The save button can be found at the bottom of each page in the survey.

Thank you for participating in the PRELUDE study and completing this questionnaire.

#### Have you been enrolled to participate in the PRELUDE study?*

( ) Yes

( ) No

### What is your study identification code?*

Your **study identification code** is the unique ID number that was given to you at your enrolment visit for the PRELUDE Study. This is a twelve digit number which you received at your first study visit to your clinic (you can also find this on your email invitation).

_________________________________________________

## Part I. DEMOGRAPHIC INFORMATION

#### Are you of Aboriginal or Torres Strait Islander background?

( ) No

( ) Yes, Aboriginal

( ) Yes, Torres Strait Islander

( ) Yes, Aboriginal and Torres Strait Islander

#### What is your cultural and ethnic background? Please select the option that you feel represents the group to which you primarily identify.

( ) Anglo-Celtic

( ) Pacific Islander

( ) British/Irish

( ) Western European

( ) Northern European

( ) Southern European

( ) South Eastern European

( ) Eastern European

( ) Arab

( ) Jewish

( ) Peoples of the Sudan

( ) Other North African and Middle Eastern

( ) Mainland South-East Asian

( ) Maritime South-East Asian

( ) Chinese Asian

( ) Other North-East Asian

( ) Southern Asian

( ) Central Asian

( ) North American

( ) South American

( ) Central American

( ) Caribbean Islander

( ) Central and West African

( ) Southern and East African

#### Where were you born?

( ) Australia

( ) New Zealand

( ) United Kingdom

( ) China

( ) Afghanistan

( ) Albania

( ) Algeria

( ) Andorra

( ) Angola

( ) Antigua and Barbuda

( ) Argentina

( ) Armenia

( ) Austria

( ) Azerbaijan

( ) Bahamas, The

( ) Bahrain

( ) Bangladesh

( ) Barbados

( ) Belarus

( ) Belgium

( ) Belize

( ) Benin

( ) Bhutan

( ) Bolivia

( ) Bosnia and Herzegovina

( ) Botswana

( ) Brazil

( ) Brunei

( ) Bulgaria

( ) Burkina Faso

( ) Burma

( ) Burundi

( ) Cambodia

( ) Cameroon

( ) Canada

( ) Cape Verde

( ) Central African Republic

( ) Chad

( ) Chile

( ) Colombia

( ) Comoros

( ) Congo, Democratic Republic of the

( ) Congo, Republic of the

( ) Costa Rica

( ) Cote d'Ivoire

( ) Croatia

( ) Cuba

( ) Curacao

( ) Cyprus

( ) Czech Republic

( ) Denmark

( ) Djibouti

( ) Dominica

( ) Dominican Republic

( ) East Timor (see Timor-Leste)

( ) Ecuador

( ) Egypt

( ) El Salvador

( ) Equatorial Guinea

( ) Eritrea

( ) Estonia

( ) Ethiopia

( ) Fiji

( ) Finland

( ) France

( ) Gabon

( ) Gambia, The

( ) Georgia

( ) Germany

( ) Ghana

( ) Greece

( ) Grenada

( ) Guatemala

( ) Guinea

( ) Guinea-Bissau

( ) Guyana

( ) Haiti

( ) Holy See

( ) Honduras

( ) Hong Kong

( ) Hungary

( ) Iceland

( ) India

( ) Indonesia

( ) Iran

( ) Iraq

( ) Ireland

( ) Israel

( ) Italy

( ) Jamaica

( ) Japan

( ) Jordan

( ) Kazakhstan

( ) Kenya

( ) Kiribati

( ) Kosovo

( ) Kuwait

( ) Kyrgyzstan

( ) Laos

( ) Latvia

( ) Lebanon

( ) Lesotho

( ) Liberia

( ) Libya

( ) Liechtenstein

( ) Lithuania

( ) Luxembourg

( ) Macau

( ) Macedonia

( ) Madagascar

( ) Malawi

( ) Malaysia

( ) Maldives

( ) Mali

( ) Malta

( ) Marshall Islands

( ) Mauritania

( ) Mauritius

( ) Mexico

( ) Micronesia

( ) Moldova

( ) Monaco

( ) Mongolia

( ) Montenegro

( ) Morocco

( ) Mozambique

( ) Namibia

( ) Nauru

( ) Nepal

( ) Netherlands

( ) Netherlands Antilles

( ) Nicaragua

( ) Niger

( ) Nigeria

( ) North Korea

( ) Norway

( ) Oman

( ) Pakistan

( ) Palau

( ) Palestinian Territories

( ) Panama

( ) Papua New Guinea

( ) Paraguay

( ) Peru

( ) Philippines

( ) Poland

( ) Portugal

( ) Qatar

( ) Romania

( ) Russia

( ) Rwanda

( ) Saint Kitts and Nevis

( ) Saint Lucia

( ) Saint Vincent and the Grenadines

( ) Samoa

( ) San Marino

( ) Sao Tome and Principe

( ) Saudi Arabia

( ) Senegal

( ) Serbia

( ) Seychelles

( ) Sierra Leone

( ) Singapore

( ) Slovakia

( ) Slovenia

( ) Solomon Islands

( ) Somalia

( ) South Africa

( ) South Korea

( ) South Sudan

( ) Spain

( ) Sri Lanka

( ) Sudan

( ) Suriname

( ) Swaziland

( ) Sweden

( ) Switzerland

( ) Syria

( ) Taiwan

( ) Tajikistan

( ) Tanzania

( ) Thailand

( ) Timor-Leste

( ) Togo

( ) Tonga

( ) Trinidad and Tobago

( ) Tunisia

( ) Turkey

( ) Turkmenistan

( ) Tuvalu

( ) Uganda

( ) Ukraine

( ) United Arab Emirates

( ) United States

( ) Uruguay

( ) Uzbekistan

( ) Vanuatu

( ) Venezuela

( ) Vietnam

( ) Yemen

( ) Zambia

( ) Zimbabwe

#### What is your current work situation? Are you...

( ) Employed - full time

( ) Employed - part time

( ) Unemployed

( ) Student

( ) On a pension or social security benefits

( ) Other (please specify): _________________________________________________

#### What is the highest level of education that you have completed?

( ) Less than year 12 high school

( ) Completed high school (year 12)

( ) Trade certificate

( ) Undergraduate degree

( ) Postgraduate degree

## SEXUAL IDENTITY

#### What is your sex?*

( ) Male

( ) Female

( ) Transgender, male-to-female

( ) Transgender, female-to-male

( ) Other (please specify): _________________________________________________

#### Are you circumcised?

( ) Yes

( ) No

( ) I don't know (please explain): _________________________________________________

#### Do you think of yourself as:*

( ) Gay/Homosexual

( ) Bisexual

( ) Heterosexual

( ) Other (please specify): _________________________________________________

## PATH 1 FOR GAY, HOMOSEXUAL AND BISEXUAL MALES, AND TRANSGENDER INDIVIDUALS

## Part II. HIV AND STI TESTING, STI DIAGNOSES

#### Prior to enrolling in this study, have you ****EVER**** had an HIV test?

Please note: This does not include the HIV test you just had as part of the screening for the PRELUDE Study.

( ) Yes

( ) No

#### Prior to enrolling in this study, how long ago were you last tested for HIV?

( ) Less than a week ago

( ) 1 - 4 weeks ago

( ) 1 - 3 months ago

( ) 4 - 6 months ago

( ) 7 - 12 months ago

( ) 1 - 2 years ago

( ) 2 - 4 years ago

( ) More than 4 years ago

#### At any time in the last three months, were you diagnosed with any of the following infections?   ****Syphilis****

( ) Yes

( ) No

#### ****Gonorrhoea****

( ) Yes

( ) No

#### ****Chlamydia****

( ) Yes

( ) No

## PART III. SEXUAL RELATIONSHIPS AND BEHAVIOUR

**In this survey you will be asked only about your male partners and we distinguish between three types of partners:**

- **A MAIN REGULAR partner (boyfriend/lover),**
- **OTHER REGULAR partners, including FRIENDS (with benefits) or FUCKBUDDIES, and**
- **CASUAL partners (those with whom a person had not had sex with before).**

#### Do you have a MAIN REGULAR partner such as a boyfriend or lover?*

( ) Yes

( ) No

#### What is the HIV status of your MAIN REGULAR partner?*

( ) HIV positive

( ) HIV negative

( ) My MAIN REGULAR partner has not been tested for HIV

( ) I do not know my MAIN REGULAR partner’s HIV status

#### In the last three months, have you had anal sex with your MAIN REGULAR partner?

( ) Yes

( ) No

**The following questions are ONLY about the sex you had with your MAIN REGULAR partner ... if you do not remember the exact number of times for each instance, then please choose the category that would be closest.**

**In the past three months, how many times did you do the following with your MAIN REGULAR partner?**

|  | **Never** | **Once** | **Twice** | **3-5 times** | **6-10 times** | **11-30 times** | **31-50 times** | **Over 50 times** |
| --- | --- | --- | --- | --- | --- | --- | --- | --- |
| I fucked him **with** a condom on | ( ) | ( ) | ( ) | ( ) | ( ) | ( ) | ( ) | ( ) |
| He fucked me **with** a condom on | ( ) | ( ) | ( ) | ( ) | ( ) | ( ) | ( ) | ( ) |
| I fucked him **without** a condom and did not cum inside him | ( ) | ( ) | ( ) | ( ) | ( ) | ( ) | ( ) | ( ) |
| He fucked me **without** a condom and did not cum inside me | ( ) | ( ) | ( ) | ( ) | ( ) | ( ) | ( ) | ( ) |
| I fucked him **without** a condom and did cum inside him | ( ) | ( ) | ( ) | ( ) | ( ) | ( ) | ( ) | ( ) |
| He fucked me **without** a condom and did cum inside me | ( ) | ( ) | ( ) | ( ) | ( ) | ( ) | ( ) | ( ) |

## SEXUAL BEHAVIOUR: OTHER REGULAR partners, Including FRIENDS (with benefits) or FUCKBUDDIES

#### In the last three months, apart from your MAIN REGULAR partner, have you had OTHER REGULAR partners, including FRIENDS (with benefits) or FUCKBUDDIES?*

( ) Yes

( ) No

## Anal Sex - All OTHER REGULAR - HIV Status Unknown

#### In the last three months, have you had any anal sex (fucking or being fucked) with any FRIENDS (with benefits) or FUCKBUDDIES whose HIV status you didn't know?

( ) Yes

( ) No

### In the last three months, how many FRIENDS (with benefits) or FUCKBUDDIES whose HIV status you did not know did you have anal sex (fucking or being fucked) with?

_________________________________________________

#### Thinking about those partners whose HIV status you did not know, how many times did you do the following things? (Please tick an answer for each row)

|  | **Never** | **Once** | **Twice** | **3-5 times** | **6-10 times** | **11-30 times** | **31-50 times** | **Over 50 times** |
| --- | --- | --- | --- | --- | --- | --- | --- | --- |
| I fucked him **with** a condom on | ( ) | ( ) | ( ) | ( ) | ( ) | ( ) | ( ) | ( ) |
| He fucked me **with** a condom on | ( ) | ( ) | ( ) | ( ) | ( ) | ( ) | ( ) | ( ) |
| I fucked him **without** a condom and did not cum inside him | ( ) | ( ) | ( ) | ( ) | ( ) | ( ) | ( ) | ( ) |
| He fucked me **without** a condom and did not cum inside me | ( ) | ( ) | ( ) | ( ) | ( ) | ( ) | ( ) | ( ) |
| I fucked him **without** a condom and did cum inside him | ( ) | ( ) | ( ) | ( ) | ( ) | ( ) | ( ) | ( ) |
| He fucked me **without** a condom and did cum inside me | ( ) | ( ) | ( ) | ( ) | ( ) | ( ) | ( ) | ( ) |

## Anal Sex - All OTHER REGULAR partners - HIV Negative

#### In the last three months, have you had any anal sex (fucking or being fucked) with any FRIENDS (with benefits) or FUCKBUDDIES who you knew/believed to be HIV negative?

( ) Yes

( ) No

### In the last three months, how many HIV negative FRIENDS (with benefits) or FUCKBUDDIES did you have anal sex with?

_________________________________________________

#### Thinking about those HIV negative partners, how many times did you do the following things?

|  | **Never** | **Once** | **Twice** | **3-5 times** | **6-10 times** | **11-30 times** | **31-50 times** | **Over 50 times** |
| --- | --- | --- | --- | --- | --- | --- | --- | --- |
| I fucked him **with** a condom on | ( ) | ( ) | ( ) | ( ) | ( ) | ( ) | ( ) | ( ) |
| He fucked me **with** a condom on | ( ) | ( ) | ( ) | ( ) | ( ) | ( ) | ( ) | ( ) |
| I fucked him **without** a condom and did not cum inside him | ( ) | ( ) | ( ) | ( ) | ( ) | ( ) | ( ) | ( ) |
| He fucked me **without** a condom and did not cum inside me | ( ) | ( ) | ( ) | ( ) | ( ) | ( ) | ( ) | ( ) |
| I fucked him **without** a condom and did cum inside him | ( ) | ( ) | ( ) | ( ) | ( ) | ( ) | ( ) | ( ) |
| He fucked me **without** a condom and did cum inside me | ( ) | ( ) | ( ) | ( ) | ( ) | ( ) | ( ) | ( ) |

## Anal Sex - All OTHER REGULAR partners - HIV Positive

#### In the last three months, have you had any anal sex (fucking or being fucked) with any OTHER REGULAR partners, including FRIENDS (with benefits) or FUCKBUDDIES who you knew/believed to be HIV positive?

( ) Yes

( ) No

### In the last three months, how many FRIENDS (with benefits) or FUCKBUDDIES who you knew/believed to be HIV positive did you have anal sex with?

_________________________________________________

#### Thinking about those HIV positive partners, how many times did you do the following things in the last three months? (Please tick an answer for each row)

|  | **Never** | **Once** | **Twice** | **3-5 times** | **6-10 times** | **11-30 times** | **31-50 times** | **Over 50 times** |
| --- | --- | --- | --- | --- | --- | --- | --- | --- |
| I fucked him **with** a condom on | ( ) | ( ) | ( ) | ( ) | ( ) | ( ) | ( ) | ( ) |
| He fucked me **with** a condom on | ( ) | ( ) | ( ) | ( ) | ( ) | ( ) | ( ) | ( ) |
| I fucked him **without** a condom and did not cum inside him | ( ) | ( ) | ( ) | ( ) | ( ) | ( ) | ( ) | ( ) |
| He fucked me **without** a condom and did not cum inside me | ( ) | ( ) | ( ) | ( ) | ( ) | ( ) | ( ) | ( ) |
| I fucked him **without** a condom and did cum inside him | ( ) | ( ) | ( ) | ( ) | ( ) | ( ) | ( ) | ( ) |
| He fucked me **without** a condom and did cum inside me | ( ) | ( ) | ( ) | ( ) | ( ) | ( ) | ( ) | ( ) |

## SEXUAL BEHAVIOUR: CASUAL partners

#### In the last three months, have you had any partners whom you can describe as CASUAL?*

( ) Yes

( ) No

## Anal Sex - CASUAL partners - HIV Status Unknown

#### In the last three months, have you had any anal sex (fucking or being fucked) with any CASUAL partners whose HIV status you didn't know?

( ) Yes

( ) No

### In the last three months, how many CASUAL partners whose HIV status you did not know did you have anal sex (fucking or being fucked) with?

_________________________________________________

#### Thinking about CASUAL partners whose HIV status you did not know, how many times did you do the following things? (Please tick an answer for each row)

|  | **Never** | **Once** | **Twice** | **3-5 times** | **6-10 times** | **11-30 times** | **31-50 times** | **Over 50 times** |
| --- | --- | --- | --- | --- | --- | --- | --- | --- |
| I fucked him **with** a condom on | ( ) | ( ) | ( ) | ( ) | ( ) | ( ) | ( ) | ( ) |
| He fucked me **with** a condom on | ( ) | ( ) | ( ) | ( ) | ( ) | ( ) | ( ) | ( ) |
| I fucked him **without** a condom and did not cum inside him | ( ) | ( ) | ( ) | ( ) | ( ) | ( ) | ( ) | ( ) |
| He fucked me **without** a condom and did not cum inside me | ( ) | ( ) | ( ) | ( ) | ( ) | ( ) | ( ) | ( ) |
| I fucked him **without** a condom and did cum inside him | ( ) | ( ) | ( ) | ( ) | ( ) | ( ) | ( ) | ( ) |
| He fucked me **without** a condom and did cum inside me | ( ) | ( ) | ( ) | ( ) | ( ) | ( ) | ( ) | ( ) |

## Anal Sex – CASUAL partners - HIV Negative

#### In the last three months, have you had any anal sex (fucking or being fucked) with any CASUAL partners who you knew/believed to be HIV negative?

( ) Yes

( ) No

### In the last three months, how many HIV negative CASUAL partners did you have anal sex with?

_________________________________________________

#### Thinking about those HIV negative CASUAL partners, how many times did you do the following things? (Please tick an answer for each row)

|  | **Never** | **Once** | **Twice** | **3-5 times** | **6-10 times** | **11-30 times** | **31-50 times** | **Over 50 times** |
| --- | --- | --- | --- | --- | --- | --- | --- | --- |
| I fucked him **with** a condom on | ( ) | ( ) | ( ) | ( ) | ( ) | ( ) | ( ) | ( ) |
| He fucked me **with** a condom on | ( ) | ( ) | ( ) | ( ) | ( ) | ( ) | ( ) | ( ) |
| I fucked him **without** a condom and did not cum inside him | ( ) | ( ) | ( ) | ( ) | ( ) | ( ) | ( ) | ( ) |
| He fucked me **without** a condom and did not cum inside me | ( ) | ( ) | ( ) | ( ) | ( ) | ( ) | ( ) | ( ) |
| I fucked him **without** a condom and did cum inside him | ( ) | ( ) | ( ) | ( ) | ( ) | ( ) | ( ) | ( ) |
| He fucked me **without** a condom and did cum inside me | ( ) | ( ) | ( ) | ( ) | ( ) | ( ) | ( ) | ( ) |

## Anal Sex - CASUAL partners - HIV Positive

#### In the last three months, have you had any anal sex (fucking or being fucked) with any CASUAL partners who you knew/believed to be HIV positive?

( ) Yes

( ) No

### In the last three months, how many CASUAL partners who you knew/believed to be HIV positive did you have anal sex with?

_________________________________________________

#### Thinking about those HIV positive CASUAL partners, how many times did you do the following things in the last three months? (Please tick an answer for each row)

|  | **Never** | **Once** | **Twice** | **3-5 times** | **6-10 times** | **11-30 times** | **31-50 times** | **Over 50 times** |
| --- | --- | --- | --- | --- | --- | --- | --- | --- |
| I fucked him **with** a condom on | ( ) | ( ) | ( ) | ( ) | ( ) | ( ) | ( ) | ( ) |
| He fucked me **with** a condom on | ( ) | ( ) | ( ) | ( ) | ( ) | ( ) | ( ) | ( ) |
| I fucked him **without** a condom and did not cum inside him | ( ) | ( ) | ( ) | ( ) | ( ) | ( ) | ( ) | ( ) |
| He fucked me **without** a condom and did not cum inside me | ( ) | ( ) | ( ) | ( ) | ( ) | ( ) | ( ) | ( ) |
| I fucked him **without** a condom and did cum inside him | ( ) | ( ) | ( ) | ( ) | ( ) | ( ) | ( ) | ( ) |
| He fucked me **without** a condom and did cum inside me | ( ) | ( ) | ( ) | ( ) | ( ) | ( ) | ( ) | ( ) |

## DRUGS, ALCOHOL AND GROUP SEX

#### In the last three months, how often have you taken any of the following drugs? (Please tick an answer for each row)

|  | **Daily** | **Nearly every day** | **3-4 times a week** | **1-2 times a week** | **2-3 times a month** | **Once a month** | **Never** |
| --- | --- | --- | --- | --- | --- | --- | --- |
| Amyl/poppers | ( ) | ( ) | ( ) | ( ) | ( ) | ( ) | ( ) |
| Viagra/Cialis etc | ( ) | ( ) | ( ) | ( ) | ( ) | ( ) | ( ) |
| Ecstasy | ( ) | ( ) | ( ) | ( ) | ( ) | ( ) | ( ) |
| Speed | ( ) | ( ) | ( ) | ( ) | ( ) | ( ) | ( ) |
| Crystal meth | ( ) | ( ) | ( ) | ( ) | ( ) | ( ) | ( ) |
| GHB | ( ) | ( ) | ( ) | ( ) | ( ) | ( ) | ( ) |
| Steroids | ( ) | ( ) | ( ) | ( ) | ( ) | ( ) | ( ) |
| Any other drug | ( ) | ( ) | ( ) | ( ) | ( ) | ( ) | ( ) |

#### In the last three months, how often have you injected any drugs?

( ) Never

( ) Once/twice

( ) At least monthly

( ) Every week

#### In the last three months, how often have you used party drugs for the purpose of sex?

( ) Never

( ) Once

( ) Twice

( ) 3 - 5 times

( ) 6 - 10 times

( ) 11 - 30 times

( ) 31 - 50 times

( ) Over 50 times

#### In the last three months, how often have you had sex involving three or more men (group sex)?

( ) Never

( ) Once

( ) Twice

( ) 3 - 5 times

( ) 6 - 10 times

( ) 11 - 30 times

( ) 31 - 50 times

( ) Over 50 times

#### In the last three months, how often have you had group sex after or while using party drugs?

( ) Never

( ) Once

( ) Twice

( ) 3 - 5 times

( ) 6 - 10 times

( ) 11 - 30 times

( ) 31 - 50 times

( ) Over 50 times

#### In the last 30 days, how often have you had a drink containing alcohol? By one drink we mean a can or glass of beer, a glass of wine, a shot of liquor, or a mixed drink with liquor?*

( ) Never

( ) Daily

( ) Nearly every day

( ) 3-4 times a week

( ) 1-2 times a week

( ) 2-3 times a month

( ) Once a month

#### On days when you drank any alcoholic beverages in the last 30 days, how many drinks did you usually have altogether? (Check one)

( ) 1-2 drinks per day

( ) 3-4 drinks per day

( ) 5-6 drinks per day

( ) 7-8 drinks per day

( ) 9-11 drinks per day

( ) 12 or more drinks per day

## PART IV: PEP (POST-EXPOSURE PROPHYLAXIS)

#### Prior to your involvement with this study, had you ever heard of PEP (Post-Exposure Prophylaxis)?

( ) Yes

( ) No

#### How many times have you ****EVER**** taken PEP in the past?

( ) Never

( ) Once

( ) Twice

( ) Three times

( ) Four times

( ) More than four times

#### In the past three months, how many times have you taken PEP?

( ) Never

( ) Once

( ) Twice

( ) Three times

## PART V: PREP (PRE-EXPOSURE PROPHYLAXIS)

#### When did you first hear about Pre-Exposure Prophylaxis (PrEP)?

( ) This is the first time I've heard of it when I was invited to take part in this study

( ) In the last 12 months before this study

( ) More than 12 months before this study

#### Have you ****EVER**** taken anti-HIV pills as PrEP in the past?*

( ) Yes

( ) No

( ) I don't know

#### Have you taken anti-HIV pills as PrEP in the last three months?

( ) Yes

( ) No

## WILLINGNESS TO TAKE PrEP

#### What would be the ideal way you would want to take anti-HIV pills as PrEP to protect you from getting HIV?

( ) Everyday

( ) For periods of time when I feel I am at high risk of getting HIV through unsafe sex

( ) Only on specific occasions when I am at high risk of getting HIV through unsafe sex

( ) For periods whilst trying to conceive

#### How long would you be willing to take anti-HIV pills daily without a break?

( ) Up to 1 month

( ) 1 to 3 months

( ) 4 to 6 months

( ) 7 to 9 months

( ) 10 to 12 months

( ) More than 12 months

### If you have any comments about this study, please write them in the text box provided below:

_______________________________________________________________________________________________________________________________________________________________________________________________________________________________________

## Thank You!
